# Protein-Structure Assisted Optimization of 4,5-Dihydroxypyrimidine-6-Carboxamide Inhibitors of Influenza Virus Endonuclease

**DOI:** 10.1038/s41598-017-17419-6

**Published:** 2017-12-07

**Authors:** Diane Beylkin, Gyanendra Kumar, Wei Zhou, Jaehyeon Park, Trushar Jeevan, Chandraiah Lagisetti, Rhodri Harfoot, Richard J. Webby, Stephen W. White, Thomas R. Webb

**Affiliations:** 1 Bioscience Division, SRI International, Menlo Park, CA 94025 USA; 2 Department of Structural Biology, St. Jude Children’s Research Hospital, Memphis, TN 38105 USA; 3 Department of Infectious Diseases, St. Jude Children’s Research Hospital, Memphis, TN 38105 USA

## Abstract

Influenza is a serious hazard to human health that causes hundreds of thousands of deaths annually. Though vaccines and current therapeutics can blunt some of the perilous impact of this viral infection, new treatments are needed due to the constantly evolving nature of this virus. Recently, our growing understanding of an essential influenza viral protein, PA, has led to the development of focused libraries of new small molecules that specifically target the active site of the PA influenza endonuclease, which we report here. Our overarching approach has been to proactively develop lead inhibitors that are less likely to rapidly develop clinical resistance by optimizing inhibitors that retain activity against induced resistant mutants. Here, we report details behind the discovery of new potent inhibitors of wild type and resistant mutant endonucleases along with their high-resolution co-crystal structure-activity relationships. These results add to our understanding of nuclease protein targets and potentially serve as starting points for a new therapeutic approach to the treatment of influenza.

## Introduction

Influenza is an infectious disease associated with 500,000 deaths and 3–4 million severe illnesses annually worldwide, despite the availability of vaccines and antiviral drugs. During the 1918 ‘Spanish flu’ pandemic, it is estimated that between 20 and 40 million people died in just eight months^1^. Other major influenza pandemics in the 20^th^ century include the 1957 ‘Asian flu,’ and the 1968 ‘Hong Kong flu’, with each resulting in approximately 1 million deaths^2–4^. In each case, 20–30% of the global population was infected within a year of the outbreak. One approach to rapidly addressing pandemics is to develop small molecule antivirals that have broad activity against all strains of influenza.

Most small molecule anti-influenza drugs currently on the market act as neuraminidase inhibitors (zanamivir, oseltamivir, peramivir) or target the M2-ion channel (amantadine, rimantadine)^5^. However, these targets, particularly the latter, are prone to rapid mutations that can confer antiviral resistance, due to the inability of the viral RNA dependent RNA polymerase (RdRp) to proofread during RNA replication. In fact, the World Health Organization’s Global Influenza Program reported that >99% of seasonal influenza A strains are now resistant to amantadine and rimantadine^6^. This has led to the search for new antiviral compounds that target other essential viral processes^7^. The influenza RdRp is itself an attractive drug target, because it is relatively slow to develop drug resistance, is conserved across genotypes, and is essential for viral replication. The influenza virus RdRp is a heterotrimer that includes the polymerase catalytic subunit (PB1), the ‘cap-binding’ subunit (PB2), and the endonuclease-containing (PA) subunit. The ‘cap-binding’ and endonuclease functionalities of RdRp work in concert to perform the essential ‘cap snatching’ of host mRNAs to generate primers for viral transcription^8,9^. In the last decade, our understanding of influenza viral RdRp has dramatically expanded through the elucidation of the high-resolution architecture of influenza endonuclease^8,10^ and, most recently, the unveiling of the complete RdRp heterotrimer structure by Cusack and coworkers^11^.

The importance of the RdRp to influenza virus viability has spawned a number of recent drug discovery efforts. Favipiravir, a broad-spectrum drug that targets numerous viral RdRps including influenza RdRp, was approved in Japan in 2014 for emergency use in the event of influenza pandemics, despite some significant concerns regarding this drug’s toxicity^12^. Also, a promising drug (VX-787)^13^ that targets the ‘cap-binding’ site of influenza RdRp is currently in advanced clinical trials^14^. However, no new influenza drug is currently available to the general population^7^.

**Figure 1:**
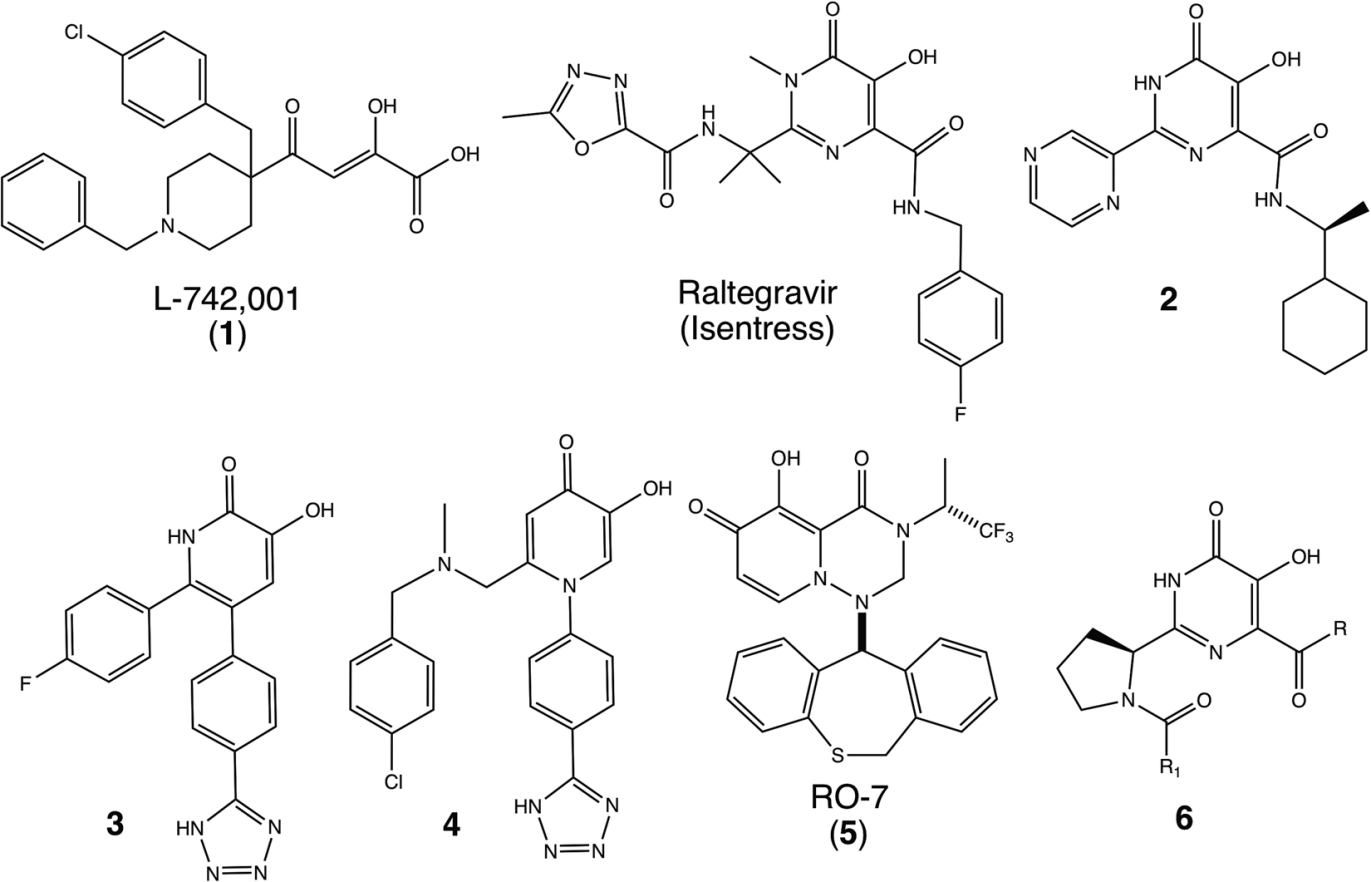
Compounds targeting the two-metal nuclease active site, including the FDA approved HIV integrase inhibitor raltegravir (Isentress)^35^, along with the first influenza lead compound in this class, L-742,001 (**1**)^46^, and several recently developed experimental influenza endonuclease inhibitors (**2** ^17^, **3** ^21^, **4** ^47^, and **5** ^48^). The general structure **6** shows the structure of the active inhibitors reported in this publication.

The essential endonuclease domain within the PA subunit is a particularly attractive drug target. It has no eukaryotic homolog, so the potential for toxicity due to off-target effects is reduced for small molecules that target its active site^9^. Using the structure of the domain determined in isolation^10,15^ and in the context of the trimeric complex^8,11^ a number of groups, including ours, have successfully used structure-assisted approaches to develop potent inhibitors^16–23^. Our efforts build off of the foundational work of Tomassini and coworkers at Merck and their early report of 2,4-dioxobutanoic acid inhibitors containing a two-metal binding pharmacophore^24–26^. We have structurally characterized the binding mode of L-742,001 (**1**, Fig. 1), the most potent inhibitor in this class,^17^ and shown that it engages highly conserved active site residues. Our recent demonstration that L-742,001 does not readily generate resistance mutations in the RdRp^27^ has provided fresh impetus to the development of endonuclease inhibitors and this effort has recently been exhaustively reviewed^28^. Figure 1 shows five endonuclease inhibitors (**1**, **2**, **3**, **4**, and **5**) that have been reported to show antiviral activity *in vitro*.

Our overarching approach has been to apply structure-based design, while optimizing inhibitors that retain activity against induced resistant mutants, in order to proactively develop lead inhibitors that are less likely to rapidly develop clinical resistance^27^. An important aspect of this work is the discovery of inhibitors that are able to potently engage previously identified sites that are conserved in resistant mutations. Here, we describe the further optimization of such a series of new endonuclease inhibitors that are an extension of our earlier published studies^17,29^. The chemical scaffold for our studies is 4,5-dihydroxypyrimidine-6-carboxamide (DHPC), which was originally developed by Summa and coworkers (Merck, Italy) into the first-in-class HIV two-metal integrase inhibitor raltegravir (Isentriss)^30^ (Fig. 1). We have previously reported active influenza endonuclease inhibitory compounds based on this DHPC scaffold^17,31^, such as **2**, and report here the identification of more potent inhibitors through iterative cycles of library synthesis, FP binding assays, FRET nuclease assays, cell-based plaque assays and inspection of inhibitor/endonuclease co-crystal structures. An important feature in the discovery of new antivirals is the ability to show potency in the face of resistance mutations. It has previously been reported that T20A mutations in influenza virus endonuclease can result from viral selection^32^ using the Merck endonuclease inhibitor^33^ (L-742,001, compound **1**, Fig. 1) and we have recently reported the mutagenesis and selection of novel compound **1** resistant mutations that also contain the T20A background^27^. For our approach, we utilized the sequence background of a wild type strain (A/California/04/2009 H1N1) that has the T20A sequence. This construct was used in our biochemical assays (FP and FRET) and in our crystal structures, so in the following discussion we refer to this as the ‘wild type’ strain. Thus, our approach starts with endonucleases that incorporate single point mutation or two point mutations so that we can identify inhibitors that can retain significant activity even when these two known resistant mutations are combined in the influenza viral endonuclease construct. We focused our attention on the E119D resistance mutation, since this mutation showed the greatest resistance in our previous studies^27^. In the following sections, we discuss our exploration and optimization chemistry in the context of a wide range of inhibitor/protein co-crystal structures which provides insight into the structure-activity relationships.

## Results

### First Generation Analogs

**Figure 2:**
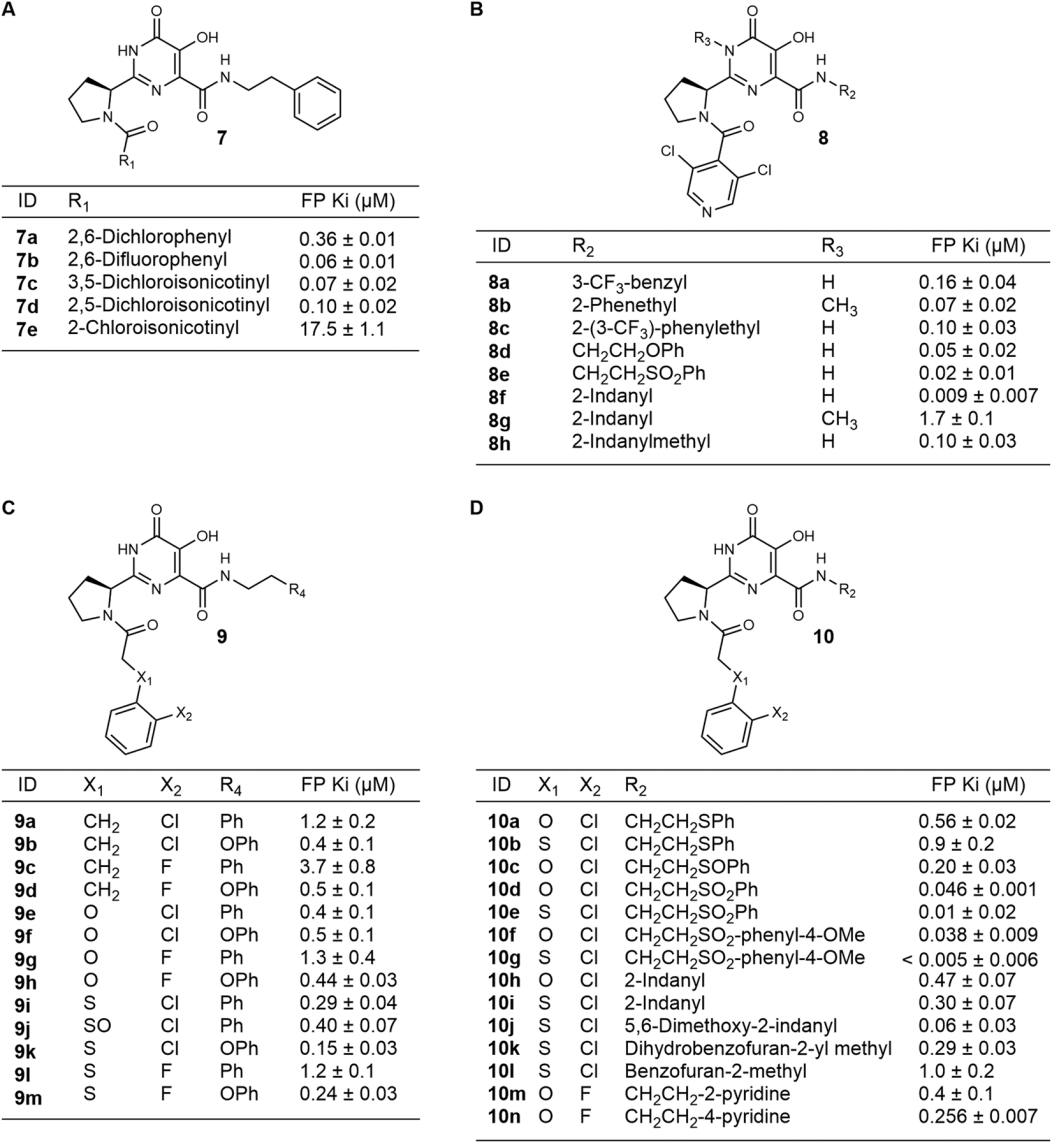
Tables A–D. Fluorescent polarization structure-activity relationships of inhibitors for wild type PA organized by small molecule 2D structural type. Table A. Initial diamides. Table B. 3,5-Dichloroisonicotinyl and N-methyl derivatives. Table C. Linker heteroatom exploration. Table D. R_2_ optimization. The fluorescent polarization assay conditions have previously been reported^17,27^.

Subsequent to our previous reports^17,34^ we prepared and screened focused libraries that included a diverse set of optically pure carboxylate analogs. Some of these carboxylates (see Supplementary Scheme 6) for example **6a**: R_1_ = 2-chlorophenoxymethyl; R = OH) showed potent PA N-terminal construct (PA_N_) binding (K_i_ < 10 nM) in our fluorescent polarization (FP) assay^17^ (which is near the lower limit of this assay) and good activity in the FRET nuclease assay (IC_50_ = 45 nM) For comparison, L-742,001 shows a K_i_ of 346 nM in the FP assay under the same assay conditions. For carboxylate analogs (e.g. general structure **6**R = OH: **6b**: R_1_ = naphthalen-1-oxymethyl, **6c**: R_1_ = 4-chorobenzyl, and **6d**: R_1_ = phenoxymethyl), we generally observed the DHPC warhead engaging the two-metal center, as expected, and the R_1_ group stacking onto Tyr24 in the crystal structures of the inhibitors in the PA_N_ binding site (Supplementary Results, Supplementary Fig. S1). This mimics the binding of a nucleotide base in the native RNA substrate, which may account for the potency of these inhibitors^20,27^. As anticipated for a carboxylate, no significant activity was observed in the viral plaque inhibition assay at 50 μM. These observations led us to initiate the synthesis of a series of diamide derivatives using previously published chemistry^29,35–37^ as shown in Supplementary Scheme 1–5. Such derivatives allowed us to introduce a second functional group at the R_2_ position to survey other binding opportunities within the endonuclease active site cavity. The structures and fluorescent binding data of a selection of the 1^st^ generation diamide analogs are shown in Fig. 2: Table A. In addition to the FP data in Table A, the biochemical inhibition of nuclease activity was confirmed for the most important compounds using the previously reported FRET assay^27^ (see Supplementary Table S1).

Initial diamide compounds such as **6e** (Fig. 1, general structure **6**: R_1_ = phenoxyacetyl, R_2_ = 2-methoxy ethylamine) showed relatively weak activity in the FP assay (K_i_ = 1.6 μM), but compounds such as **6f** (Fig. 1, general structure **6**: R_1_ = 2,6-dichlorobenzoyl, R_2_ = 2-phenethylamine) showed improved binding (K_i_ = 0.36 μM) for the amide series, although the potency was generally not as high as the corresponding carboxylate analogs. In order to determine the optimal R_1_ functionality for the pyrrolidine amide, a variety of compounds with different side chains were synthesized, while the R_2_ phenethyl side chain was held constant. Based on the potency of **6f**, a number of similar R_1_ aryl groups, **7b-7e**, were tested (see Fig. 2: Table A).

**Figure 3:**
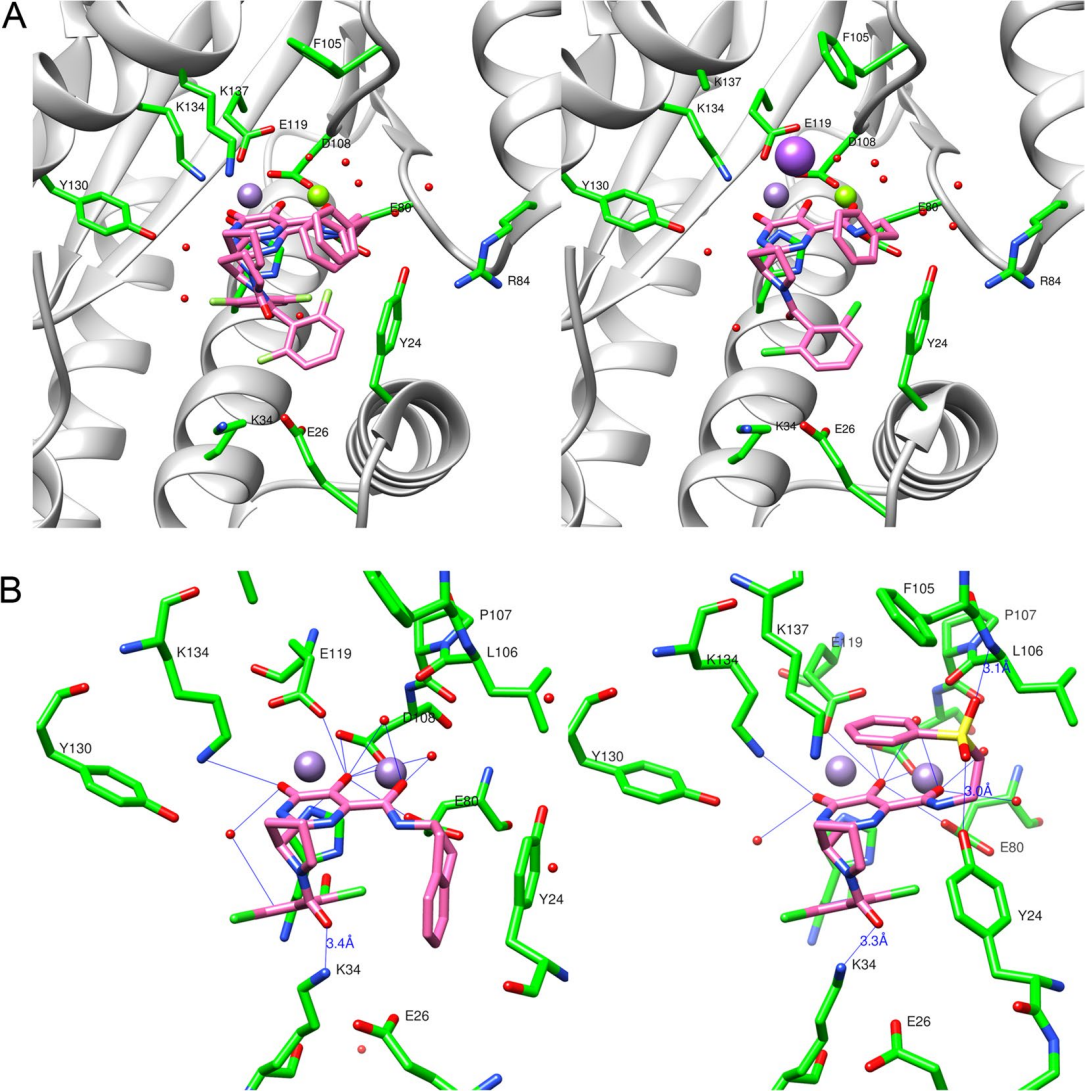
The wild type PA_N_ high affinity bound conformations of inhibitors from high-resolution co-crystal structures. (**A**) The PA_N_ inhibitor co-crystal structures of **7b** (left panel, which shows the cis (44%) and trans (56%) bound conformations) and **7a** (right panel, which shows the trans bound conformation). (**B**) The bound conformation of **8f** (left panel) in the PA_N_ active site and the bound conformation of **8e** (right panel) in the wild type PA_N_ construct showing potential hydrogen-bond interactions with crystallographic water molecules, active-site side chains, and a main chain interaction. Both structures feature H-bond donation from Lys34 to the pyrrolidine amide carbonyl (3.4 Å and 3.3 Å, respectively), and numerous interactions near the two-metal site, which are common to all DHPC inhibitors. In **8e**, note the main chain (Leu106) H-bond (3.1 Å) to one sulfone oxygen and the H-bond (3.0 Å) donation from Tyr24 the other sulfone oxygen. Mn^2++^ ions are shown as violet spheres and Mg^2++^ as green spheres. See Supporting Figure S1b for electron density maps for examples of these and other relevant structures.

In this series, the identity and locations of the halogens were changed and a heteroatom was added to help improve solubility. Overall, the presence of a nitrogen heterocycle improved potency in the FP assay and the position and number of halogens was found to be extremely important for potency (as seen in **7d** vs. **7e**). The difluoro compound (**7b**) was observed to be significantly more potent than the initial dichloro compound (**7a**), so the crystal structures of these compounds in the PA_N_ active site were obtained to investigate their modes of binding (see Fig. 3A, Fig. S1b). Although the protein structures, including the two metal binding sites, are nearly identical, the R_1_ group of **7b** adopts both cis (44%) and trans (56%) amide bond conformations; while **7a** only adopts the trans pyrrolidine amide conformation. For simplicity we will refer to these conformations in the following discussion simply as cis and trans, respectively. In the cis conformation of **7b** the difluorophenyl group stacks against the metal-chelating heterocylic core, while in the trans conformations of **7a** and **7b**, the R_1_ aryl group extends into a pocket created by Tyr24, Glu26, and Lys34. Regardless of the cis or trans conformation, the R_2_ 2-phenethyl stacks against Tyr24. While this interaction is clearly important to the potency of these inhibitors, we wanted to see if we could improve the tightness of this interaction or pick up any new interactions in this binding pocket, by modification of the R_2_ group.

### Second Generation Diamide Analogs

We next chose to explore alternatives to the R_2_ phenethyl group by preparing the analogs shown in Fig. 2: Table B, while holding the R_1_ group constant as 3,5-dichloroisonicotinyl to give compounds of the general structure **8**. The goal of this stage of exploration was to identify higher affinity compounds by the introduction of conformational constraints and groups that could enhance aqueous solubility. First, we explored conformational constraints of the phenethyl group, which led to the synthesis of indanyl derivatives **8f** and **8h**, which were found to be potent endonuclease binders. **8f** was one of our most potent inhibitors (K_i_ = 9 nM) and so we obtained a crystal structure of it in complex with the wild type PA_N_ complex (Fig. 3B). **8f** adopts the cis conformation observed in **7b**, with a few key differences in the R_1_ positioning: the pyrrolidine is now positioned in a way that places the R_1_ carbonyl close enough to Lys34 (3.4 Å) to pick up a hydrogen-bond. Additionally, due to the conformational constraint, the R_2_ phenyl ring is now in a position to stack directly underneath Tyr24, creating a tight binding interaction.

We also explored the alkylation of the 5,6-dihydroxypyrimidine amide nitrogen, since another consideration in the development of orally active compounds is the reduction of the number of hydrogen-bond donors (as part of the rule of five)^38^. As shown by compounds **8b** and **8g**, the presence of the methyl group severely impacts the potency of these compounds, resulting in a dramatic loss of binding affinity as measured by FP when compared to the corresponding unmethylated analogs **7c** and **8f**. This is consistent with our observation of the co-crystal structures in which the amide NH group interacts with the OH group of Tyr130.

Notably, the R_2_ sulfone derivative **8e** (Fig. 3B and Fig. S1b), which was introduced to improve solubility, showed very potent binding with K_i_ = 19 nM. The co-crystal structure of **8e** revealed a remarkable stacked ‘sandwich’ structure in which the R_1_ and R_2_ groups stack on opposite surfaces of the warhead. This bound conformation may be driven, in part, by the energetic benefit of the two sulfone oxygens, which accept hydrogen bonds from the OH group of Tyr24 (3.0 Å) and the backbone amide of Leu106 (3.1 Å). In order to make the hydrogen bond with the sulfone oxygen, Tyr24 moves closer to the warhead than in similar structures such as **7a** or **8f**. Additionally, the cis conformation of this inhibitor places the pyrrolidine amide in a position to pick up a hydrogen bond between the carbonyl and Lys34 (3.3 Å).

In order to evaluate the antiviral activity relationships of these inhibitors on viral replication in living cells, we also performed plaque inhibition assays on selected FP active compounds from Fig. 2: Tables A and B (also see Supplementary Fig. S2A) against wild type A/Puerto Rico/8/34 (H1N1)^27^. Inhibition of wild type virus growth in the micromolar range was seen for almost all of these active inhibitors apart from **8e**, which showed no effect. With an FP K_i_ value in the low nanomolar range but no inhibition in plaque assay, it is likely that **8e** has particularly poor transport into cells. However, we were not discouraged from using the sulfone group as we hoped to improve the cell permeability through the use of more polar R_1_ groups. The results up to this point, particularly the success of the indanyl and sulfonyl R_2_ groups, were used to guide the design of the next iteration of endonuclease inhibitors.

### Aryl Linker Exploration

In order to expand the diversity of options for lead optimization, we next sought to explore R_1_ groups with similarities to our initial carboxylate hits, such as **6d**. For this series we chose to alter the aryl linker atom, X_1_, and the halogen substituent X2 (Fig. 2: Table C). We also hoped that the addition of an electron donor to the R_2_ phenyl ring would increase the affinity of this group for Tyr24, based on the interactions that we observed with compounds such as **7a**, so we tested both R_4_ = Ph and R_4_ = OPh. The relevant compound library synthesized and their FP binding affinities are summarized in Fig. 2: Table C. The following general trends were observed: the optimal X_1_ linker atom is sulfur, chlorine is preferred over fluorine as an X_2_ substituent, and a phenoxy group is preferred over a phenyl group in the R_2_ substituent. Thus compound **9k**, which combines all of these features, shows the most potent binding for this series with a K_i_ = 151 nM.

In regard to the binding conformations of similar analogs, we found that the co-crystal structures of inhibitors that differ only by a single atom *type* (Cl vs F, or CH_2_ vs O vs S) show very similar bound conformations to each other. For example, the phenoxy analogs with X_2_ = Cl and different X_1_ groups, **9b** (X_1_ = CH_2_), **9f** (X_1_ = O), and **9k** (X_1_ = S) all have a cis pyrrolidine amide bond, which orients the R_1_ group beneath the heterocyclic core, and also have a similar tight stacking interaction of the phenoxy group with Tyr24 (see Supplementary Fig. S3). Similarly, the change from X_2_ = Cl to X_2_ = F does not seem to have any impact on the binding of these compounds, as seen in the similar binding conformations of **9f** (X_1_ = O, X_2_ = Cl) and **9h** (X_1_ = O, X_2_ = F). In contrast, the inhibitor-protein crystal structure of the less potent phenethyl analog **9e** (X_1_ = O, X_2_ = Cl) shows a very different protein-bound conformation when compared to the phenoxy analogs (see Supplementary Fig. S3). In this case, the trans conformation is adopted and the phenyl groups from R_1_ and R_2_ both interact with Tyr24.

### Aryl Linker Optimization

The ultimate goal in the development of an endonuclease inhibitor is the identification of a potent, selective *and* orally active agent; we therefore focused on the introduction of functionality that should improve the aqueous solubility of our compounds while maintaining potent target (PA_N_) binding. As shown in Fig. 2: Table C, we prepared a set of compounds that combine the features found in our most potent compounds from Fig. 2: Table C, while incorporating strategic modifications to the R_2_ group designed to enhance solubility. Due to the potency of **9f** and **9k**, we first looked at making compounds with groups that might have similar tight stacking interactions with Tyr24. This led us to replace the oxygen with sulfur, which decreased the potency. However, a single ‘oxidation’ of **10a** yielded the sulfoxide **10c**, which is significantly more potent in FP binding and should improve solubility. Further ‘oxidation’ produced the corresponding sulfone derivative, **10d**, which showed additional improvement in binding potency (see Fig. 2: Table C). When a methoxy group was added to the phenyl ring of the sulfone to give **10f**, the potency continued to improve. A similar trend was also observed for the X_1_ = S derivatives (i.e. **9k**, **10b**, and **10e**) which led to the discovery of **10g**, which reached the lower limit of our FP assay (~5 nM). This ‘oxidation’ also had a significant impact on the bound conformation of the inhibitor (see Fig. 3A and Fig. S1c). Instead of stacking with Tyr24 as in **9k**, the phenyl ring in **10e** stacks above the warhead, in a similar position to our previous sulfone compound **8e**. The two bound conformations of **10e**, which are present in equal amounts, have nearly identical placement of the R_2_ group and the warhead with the same new hydrogen bonds from Tyr24 and the backbone of Leu106 to the sulfone oxygens. However, in the cis conformation, the R_1_ aryl group stacks beneath the warhead, while in the trans conformation, it stacks with Tyr24. This allows the cis conformation to pick up a hydrogen bond with Lys34 and the R_1_ carbonyl, and the trans conformation to obtain a favorable interaction between the aryl chloride and Glu26.

**Figure 4:**
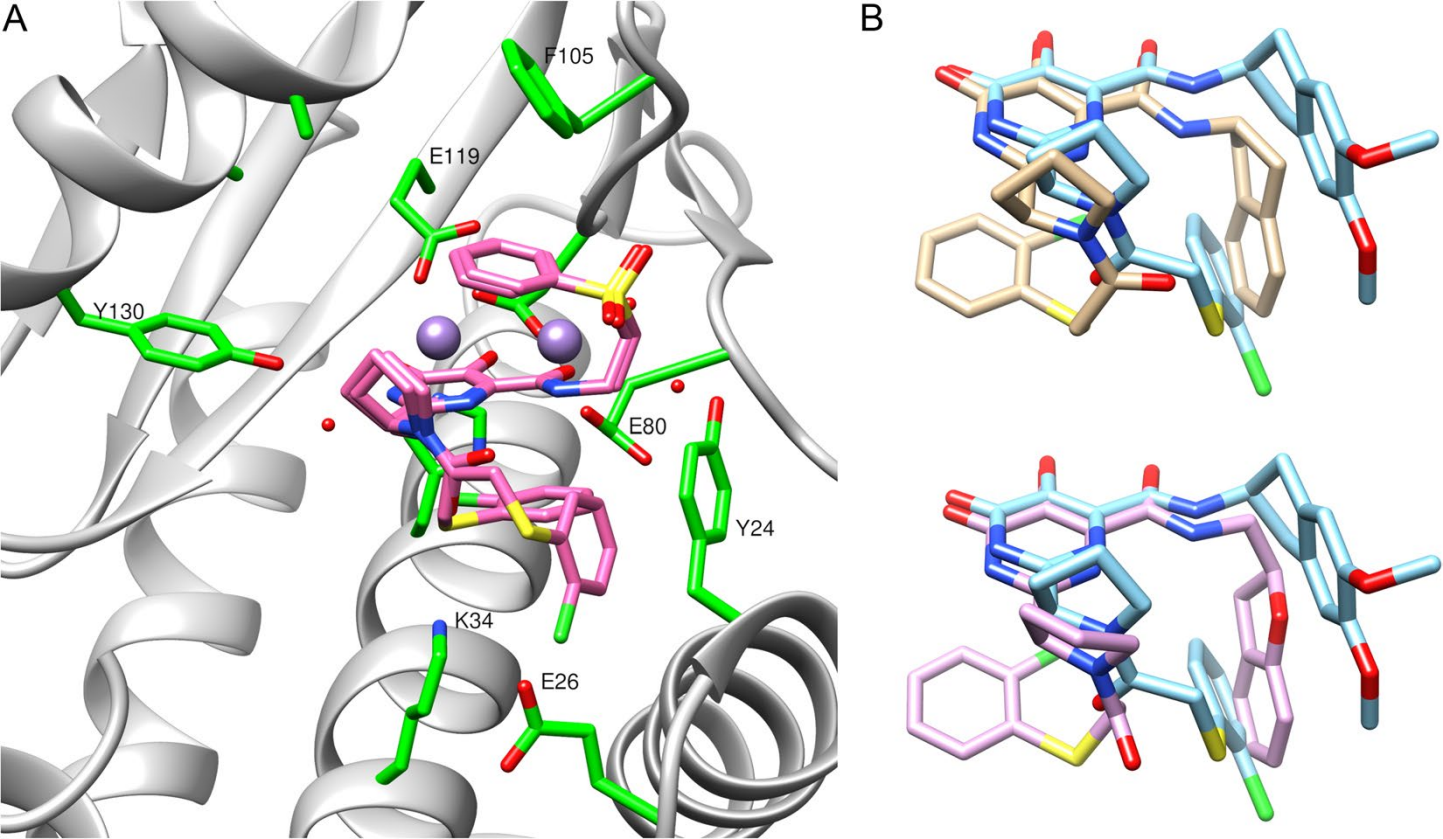
Examples of some aryl linker bound conformations with wild type PA. (**A**) A depiction of the co-crystal structure showing two binding modes of **10e** with comparable binding energies in the wild type PA_N_; showing active-site side-chains Tyr111 on the left and Tyr24 on the right. (B) Top Panel: Overlay of the bound conformation of conformationally constrained phenethylamine analogs **10i** (tan) and **10j** (cyan). Bottom Panel: Overlay of **10j** (cyan) with **10k** (purple).

We next tested compounds with conformational constraints, due to our success with these groups when R_1_ = 3,5-dichloroisonicotinyl (e.g. **8f**). In this case, the 2-indanyl analogs **10h** and **10i** were not as potent as the conformationally unconstrained phenethyl analogs. However, when two methoxy groups were added to the indane (**10j**) the binding activity improved significantly. Because of the dramatic improvement in potency, we obtained the crystal structures to see if there was any change in the binding modes of the inhibitors. We found that **10j** (Fig. 4B) binds the endonuclease in a very similar fashion to **9e**, with a trans configuration and the R_1_ and R_2_ groups sharing the interaction with Tyr24. In this structure, the R_1_ carbonyl has a close interaction with Lys34 (2.7 Å) and the R_1_ chlorine has an interaction with Glu26. In contrast, the less potent inhibitors with dihydrobenzofuran (**10k**) or indane (**10i**) substituents in R_2_, adopt a cis conformation with the R_1_ aryl chloride stacking underneath the warhead and the R_2_ group stacking against Tyr24, in a similar position to the R_1_ aryl chloride in **10j**. When compared to **8f**, these two inhibitors have identical positioning of the warhead and the R_2_ indanyl groups, but they differ in the positioning of the pyrrolidine ring, causing the loss of the carbonyl-Lys34 interaction that was found in **8f**. As a part of our effort to improve solubility, we also tested pyridine analogs **10m** and **10n**, which showed improved potency over the corresponding phenyl analog **9g**.

### Structure-activity Relationships with the Point Mutant E119D and Comparison of Wild Type and E119D Mutant Inhibitor Co-crystal Structures

**Figure 5:**
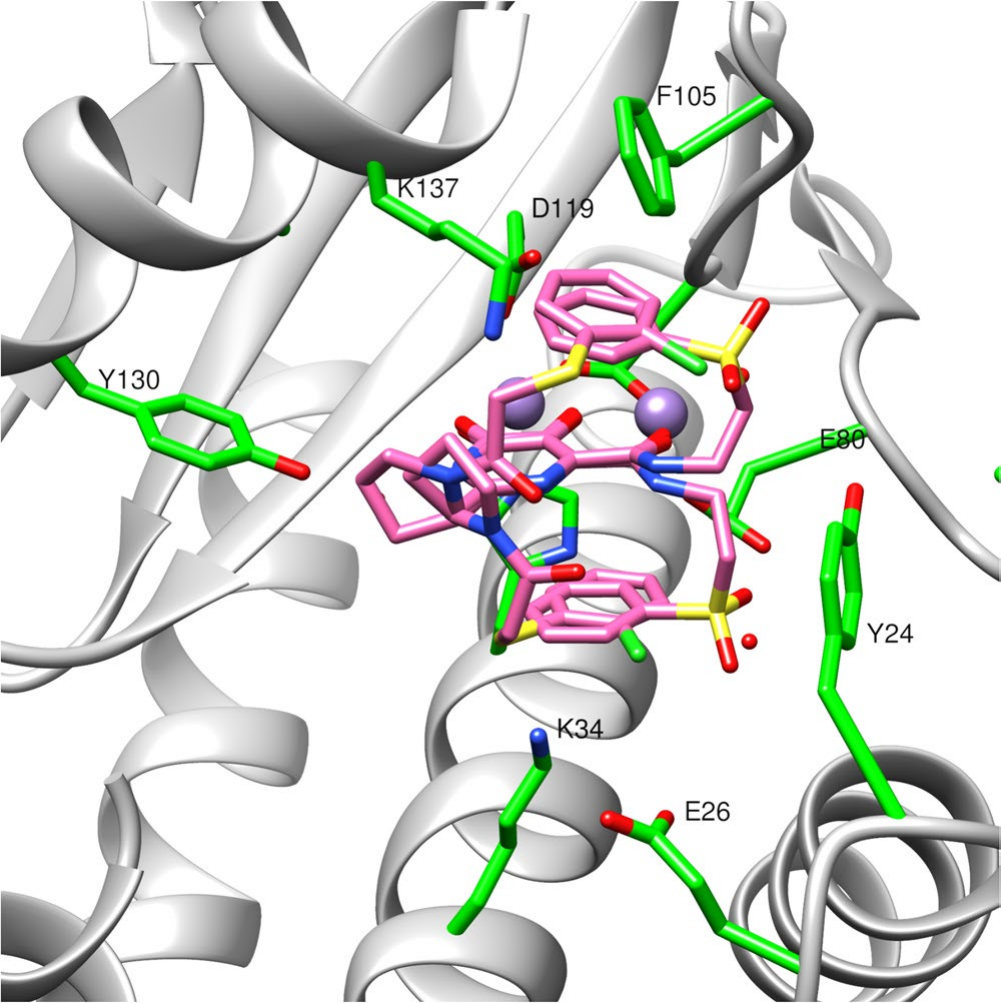
The bound conformations (cis = 49%, inverted = 51%) of **10e** in complex with the E119D mutant of PA_N_ construct. The cis mutant conformation includes a chloro carbonyl intramolecular interaction that is not present in the wild type structure with the same inhibitor.

We also chose to examine the crystal structures of various inhibitors in complex with the E119D mutant. Overall, the impact of this point mutation on the bound conformations of our inhibitors is highly variable. For example, two of our sulfone inhibitors (**8e** and **10e**) respond in different ways to the mutation. In the case of **8e** (Supplementary Fig. S4), which has a cis “sandwich” structure, only a small change in the pyrrolidine ring was observed in the complex with the E119D mutant, while the R_1_ aryl ring and the R_2_ phenyl sulfone remained in the same position. For **10e** (Fig. 5 and Fig S1d), which has a more flexible R_1_ group, the mutant has two conformations that both differ from the wild type and are present in nearly equal amounts (cis = 49%, inverted = 51%). In the cis wild type and mutant conformations, the R_2_ group maintains its position and the “sandwich” structure is adopted, but the 2-chloro aryl group in the mutant is flipped so that the chlorine points towards the R_1_ carbonyl, picking up a favorable intramolecular interaction. This cis conformation with the chlorine-carbonyl interaction is not found in any of the wild type structures we obtained. The other conformation adopted by the **10e** mutant complex is very unusual, with the R_1_ and R_2_ groups in inverted positions: the R_2_ sulfone stacks underneath the warhead and the R_1_ aryl chloride stacks above the warhead.

**Figure 6:**
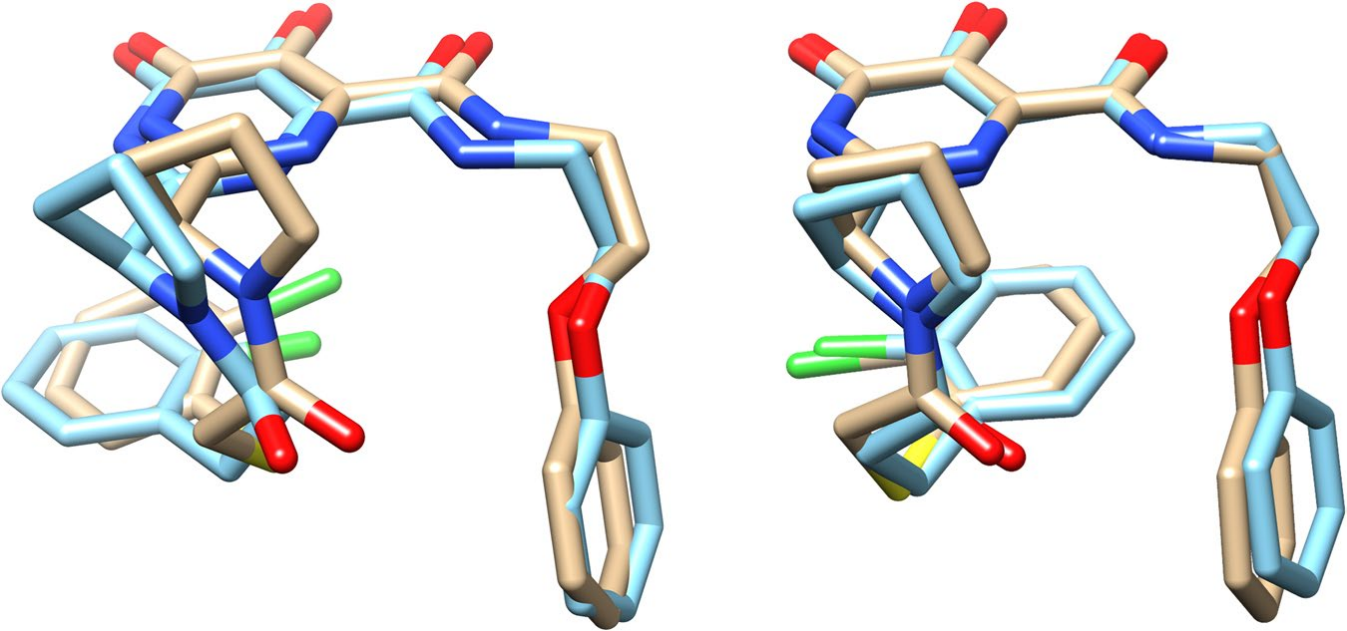
A comparison of bound high affinity wild type versus E119D inhibitor conformations. Left Panel: The co-crystal structure of **9k** (tan) overlaid with the conformation **9b** (cyan) in the wild type PA_N_ construct. Right Panel: The conformation of **9b** (cyan) overlaid with the conformation of **9k** (tan) in the E119D mutant. Note that the amide bond with the 2-Cl phenyl group is cis in both the wild type and mutant structures.

Another set of compounds that undergo significant changes in the mutant structures are **9k** and **9b (**Fig. 6 and Fig. S1e). In the wild type and the mutant structures for these compounds, the R_2_ phenoxy group maintains the same position, stacking with Tyr24, and the R_1_ 2-chloro aryl group adopts the cis conformation and stacks underneath the warhead. However, for the wild type structures, the 2-chloro group is pointed towards the R_2_ substituent, while in the mutant structures the 2-chloro group is directed away from R_2._ For one of our most potent inhibitors, **10j** (Fig. 2: Table D), the mutant and the wild type structures both have the R_2_ dimethoxy indane group and the R_1_ 2-chloro aryl group in similar positions, interacting with Tyr24 (see Supplementary Fig. 5). The most significant difference between these structures lies in the position of Lys34: only the wild type inhibitor is in a position to pick up a key interaction between the R_1_ carbonyl and Lys34.

### Comparison of Wild Type Versus E119D Mutant Endonuclease FP Binding and Cell-based Activity of Inhibitors

We also obtained FP binding data for many of the inhibitors against the E119D mutant (see Supplementary Table S2). As seen by inspection of this table, the affinity of these inhibitors is somewhat reduced, as expected^27^, but the activity ranking of FP potency is very similar for both wild type and mutant. In the mutant FP binding assay, our most potent compounds from the wild type assay including sulfone compounds **8e**, **10e**, and **9k** and indane **8f** retain their potency. In contrast, indanyl derivatives **10i** and **10k** and phenyl derivative **9e** see the largest drops in potency. We also tested many of the compounds from Fig. 2: Table C and Fig. 2: Table D in the plaque inhibition assay against both wild type A/Puerto Rico/8/34 (H1N1) and the endonuclease mutant bearing an E119D PA substitution^27^ (Supplementary Fig. S2B). In the wild type assay, most of the compounds showed IC_50_’s in the low micromolar range (IC_50_ = 6.6–38.0 µM). Notably, some compounds such as **9k** and **9b** with relatively high FP K_i_ values (0.151 µM and 0.376 µM respectively) show similar plaque IC_50_ values (7.2 µM and 6.6 µM respectively) to compounds with lower FP Ki values, such as **10g** (FP K_i_ = < 0.005 µM, plaque IC_50_ = 9.1 µM). This may indicate that **9k** and **9b** have better cell permeability than **10g**. These results emphasize the importance of the early integration of cell-based assays into lead optimization, rather than the sole reliance on cell-free biochemical assays for this purpose^39^. Overall linker optimization targeting increased solubility generally led to improvements in inhibition of viral growth, especially for compounds like **9k** and **10j**, in line with the results of the FP data and crystal structures. Generally, activity against the E119D mutant was reduced in comparison to the wild type, but at comparable levels to L-742,001.

## Discussion

### First and Second Generation Endonuclease inhibitors

The initial diamide inhibitor FP binding data (Fig. 2: Table A) showed the importance of the aryl ortho halogens and, independently, the benefit of having heteroatoms in the aryl ring for tight binding. Aryl heterocycles may also improve aqueous solubility, which is one of our key design considerations since water solubility is an important property that is associated with good oral activity in drug leads^40^.

For this type of inhibitor, with R_1_ = aryl and R_2_ = 2-phenethyl, we observed that the two bound conformations of **7a** and **7b** (Fig. 3A) appear to be representative of the most common binding modes for the series. However, it is not clear what causes an inhibitor to adopt the cis conformation versus the trans conformation, and in general, there does not appear to be a significant advantage to binding in one conformation over the other. These results starkly demonstrate that a modest change (e.g. from chlorine to fluorine) can have a dramatic impact on the small molecule bound conformation and that there is a need for caution when assuming that an analog series share a similar and common binding mode, which is the usual assumption in medicinal chemistry when structural data are not available.

When we moved to other R_2_ groups, we observed that the length of the linker to the phenyl ring from the amide is very important; since shorter (e.g. **8a**) chains generally showed lower potency than their counterparts with 2–3 atom linkers. This suggests that a 2–3 atom linker is likely the ideal range to orient the phenyl ring so that it can interact favorably with Tyr24. The sulfone R_2_ group was an interesting discovery due to its atypical ‘sandwich’ structure and its high potency. This increase in potency is likely due to the new hydrogen bonding interactions the sulfone oxygens are able to achieve in the binding site with the backbone amide of Leu106 and with the OH of Tyr24. It should be noted that the sulfone is analogous to a phosphate group and its mode of binding could therefore reveal how the phosphate of the upstream nucleotide of the mRNA substrate binds adjacent to the active site.

### Aryl Linker Optimization

Examining a selection of crystal structures from Fig. 2: Tables C and D, we found that most of the inhibitors with this type of R_1_ group, with the exception of the sulfones, adopt binding modes similar to either **9b** or **9e** (Supplementary Fig. S2). For compounds in which the R_2_ phenyl group interacts weakly with Tyr24, such as **9e** and **10j**, the R_1_ amide bond adopts a trans conformation, which allows the R_1_ phenyl group to interact with Tyr24 as well. In contrast to this observation we noted that if the stacking interaction with R_2_ is tight, as for **9b** or **10i**, the R_1_ group will adopt a cis conformation and the R_1_ phenyl ring will stack beneath the warhead. The exception to this is the sulfone in this series, **10e**, which adopts two significantly different binding modes (Fig. 5A) that are more similar to **8e**, with the sulfone stacked above the warhead. For the trans conformation, the R_1_ group is still in an identical position to **9e**; but for the cis conformation, the R_1_ group is much farther underneath the warhead than in **9b**, almost directly underneath the R_2_ phenyl sulfone.

As previously discussed, we discovered that the most potent R_1_ linker was one with X_1_ = S and X_2_ = Cl, even though there is often no significant difference between crystal structures with this R_1_ group and other similar R_1_ groups (see **9b** vs **9f** vs **9h** vs **9k**, Supplementary Fig. S2). The most potent binding inhibitors in this compound series, **10e**, **10 g** and **10j** (Fig. 2: Table D), all contain sulfur and chlorine atoms at the X_1_ and X_2_ positions of the R_1_ group, respectively. Also, the sulfone group once again seems to be extremely favorable in terms of binding affinity.

### Structure-activity Relationships with the Point Mutant E119D and Comparison of Wild Type and E119D Mutant Inhibitor Co-crystal Structures

Previous studies identified an active-site residue mutation (T20A) in PA that was induced under pressure of an inhibitor^32^ and is also observed in some wild type influenza strains^27^. We reported that L-742,001 pressure combined with random mutagenesis was required to generate the novel resistant mutation E119D, which displayed significant resistance to L-742,001^27^. Though we found it possible to induce this mutation in the active site of the RdRp, less than 1% of sequenced influenza viruses contain the resistant residue naturally^27^. The E119D mutation is directly adjacent to the two-metal site of the endonuclease and we expected a large effect on the binding modes of inhibitors since this mutation has been shown to affect the binding of mononucleotides^27^. In order to design inhibitors that can regain activity against influenza strains with this mutation, we focused on exploiting the pocket created by the highly conserved Tyr24, Glu26, and Lys34 residues, which form key interactions with the bound mononucleotide and are therefore may be less likely to mutate under clinical drug pressure.

In summary, we have synthesized and tested a number of focused 2-(pyrrolidin-2-yl)-5,6-dihydroxypyrimidine-4-carboxamide libraries in a structure-guided fashion and obtained compounds with potent binding activity that also show strong biochemical nuclease inhibition and antiviral activity in cells *in vitro*. Several inhibitors have been identified that show >40 fold increase in the binding K_i_ when compared to the well-studied Merck inhibitor, L-742,001^24^. The approach we followed has been to proactively develop lead inhibitors that are less likely to quickly develop clinical resistance by selecting compounds that retain good activity against artificially induced resistant mutant proteins and mutant virus *in vitro* ^27^. We have also developed an improved understanding of the molecular determinants of inhibition for both the wild type and E119D mutant inhibitor resistant influenza endonucleases while we explored several lead chemotypes and pharmacophores that interact with conserved residues in the active site of PA. In the course of this work we have also observed unique binding conformations of small molecule inhibitors bound to the endonuclease constructs and fortuitously discovered several new types of tight-binding interactions. We believe that the results presented in these studies will be useful for other members of the scientific community involved in the discovery of improved inhibitors of endonuclease and other homologous targets. Additionally, these new inhibitors together with the associated atomic resolution 3D structure-activity data, may dramatically facilitate the development of an important new therapeutic approach to the treatment of influenza.

## Methods

Methods, including statements of data availability and any associated accession codes and references, are available in the online version of the paper.

### Fluorescence Polarization Binding Assay

Wild type PA_N_ protein and E119D-PA_N_^ΔLoop^ were used to obtain the dissociation constants of the compounds. The reagents, experimental protocol and data analysis were performed as previously reported^17,27^.

### PA_N_ nuclease activity assay

The nuclease activity assay was slightly modified from previously reported methods^17,27^. The fluorescently-labeled synthetic oligonucleotide 5′-Cy5-GAATACTCAAGCTATGCATC-3IAbRQSp was obtained from Integrated DNA Technologies (Coralville, IA) and dissolved in 10 mM Tris-HCl, pH 7.0. 2 μl of 4 µM substrates (10x with 400 nM final concentration) were added to a 384-well, black, low volume microplate (Greiner #784076). Subsequently, 2 µl compounds in DMSO at 10x final concentrations were added to the plate. The reactions were started by adding 31.25 nM wild type PA_N_^ΔLoop^ protein (1.25x with 25 nM final concentration) in 16 μl per well master mix. The master mix contained 10 mM Tris-HCl, pH 8.0, 100 mM NaCl, 10 mM β-mercaptoethanol, 2.5 mM MnCl_2_, and 0.25 mg/mL bovine serum albumin. Plates were covered with clear adhesive film and the fluorescence was monitored using an EnVision Multilabel reader (Perkin-Elmer, Waltham, MA) at 37 °C by exciting Fluorescein at 485 ± 7 nm (Perkin-Elmer filter#102), and monitoring fluorescence intensity at 535 ± 12.5 nm (Perkin Elmer filter#206). The plate was read every minute for 60 minutes and signal gain was determined by scanning the plate for the strongest signal. All assays were carried out in triplicate. Relative fluorescence units (RFU) between 10 to 60 minutes were fitted with linear regression to obtain the reaction rate. Reaction rates were averaged and plotted against the corresponding compound concentration and fitted with four parameter logistic curve to obtain IC_50_.

### Cloning, expression and purification of PA_N_ endonuclease domain

PA_N_ endonuclease domain from A/California/04/2009 H1N1 (wild type PA_N_), PA_N_ domain with a loop deletion (PA_N_^ΔLoop^, residues 51–72 replaced with a GGS linker), and E119D-PA_N_^ΔLoop^ were cloned in pET52b vector with a cleavable C-Terminal His-tag and transformed into BL21(DE3). The protein was expressed in LB medium overnight at 18 °C after induction at an OD_600_ ~0.8 with 0.2 mM isopropyl-β-thiogalactopyranoside (IPTG). The protein was purified from cell lysates by HisTrap affinity chromatography and the His-tag was removed by digestion with thrombin. The protein was further purified by gel filtration using a Superdex 75 size-exclusion chromatography column in 20 mM Tris pH 8.0, 150 mM NaCl and 1 mM TCEP. The same domain was also cloned in pET28a vector with a N-Terminal His-tag, purified similarly in a buffer containing 20 mM HEPES pH 7.8, 200 mM NaCl, 2 mM TCEP and 1 mM EDTA, but without cleaving the His-tag and used for co-crystallization of the protein in complex with various inhibitors.

### Crystallization of PA_N_ endonuclease domain with inhibitors

The proteins from either construct were concentrated to ~10 mg/ml for crystallization. For crystal structure determination, the proteins were either pre-incubated with 1.2 mM inhibitor or crystals of the holo-enzyme were soaked in the crystallization solution containing 3 mM inhibitor overnight. Crystals were obtained by using hanging drop vapor diffusion method. 2 μl drops were set up by mixing protein and crystallization solution in 1:1 or 2:1 ratio on a cover slide that hangs over 500 μl well solution. Crystals appeared in 1–3 days and grew to maximum in 4–6 days. For the X-ray diffraction experiment, crystals were cryo-protected in crystallization solution containing 25% ethylene glycol (PEG conditions) and 30% glycerol (Ammonium sulfate condition), or a mixture of crystallization solution, 3.4 M Sodium malonate (pH 7.0) and glycerol in 1:1:1 ratio (Succinic acid condition), and flash frozen in liquid nitrogen. Detailed information on crystallization solution for each protein-inhibitor complex is provided in Supplementary Table S3.

### X-Ray data collection and refinement

The X-ray diffraction data were collected at a wavelength of 1.000, 0.97903 or 0.9789 Å at a temperature of 100 Kelvin at the 22-ID and 22-BM beam lines maintained by SERCAT (Southeast Regional Collaborative Access Team) at the Advanced Photon Source, Argonne National Laboratory, USA. The data were indexed, integrated and scaled using the HKL2000 suite of programs^41^. After molecular replacement with Phaser^42^, refinement and model building was completed using Refmac^43^ or Phenix^44^ and Coot^45^. The details of the data collection, refinement statistics and the PDB IDs of the crystal structures deposited at the wwPDB are given in Supplementary Tables S4–S12.

### Compound Library Synthesis

Compounds were prepared as previously reported^29^. Detailed experimental procedures and characterization data can be found in the Supplementary Materials file.

### Plaque reduction assay

Egg propagated stocks of influenza virus A/Puerto Rico/8/34 (H1N1), or a reverse genetics version containing the E119D PA mutant, were diluted to 100 pfu/ml in infection medium (minimum essential medium (MEM; Invitrogen), 4% BSA, 100 units/ml penicillin, 100 units/ml streptomycin, 0.25 µg/ml Amphotericin B, 1x MEM vitamins, 2 mM l-glutamine) and 1.0 ml inoculated onto confluent MDCK (ATCC; CCL-34) cells in 6-well plates. Inoculations were performed for 1 hour at 37 °C/5% CO_2_, then wells washed with PBS twice before overlaying with infection medium containing: drug, 1% low melting point agarose (Lonza), and 1 µg/ml TPCK-trypsin. Agarose overlays were set at room temperature for 15 min and incubated at 37 °C/5% CO_2_ for 72 hours. Plaques were visualized by removing overlay and staining with a 1% crystal violet/2% formalin solution in PBS for 1 hour. Plaques were then gently washed in RO water and allowed to dry. Plaques were counted manually and percent reduction calculated compared to infected only control. Statistical analyses were performed using GraphPad Prism using a two-way ANOVA (α = 0.05) with Sidak post-hoc tests to compare wild type and E119D for each drug concentration.

### Cytotoxicity assay

Toxicity of drugs was assessed using a MTT based *in vitro* toxicology assay kit (Sigma-Aldrich) using MDCK cells incubated with the drug at the indicated concentrations for 72 hours at 37 °C/5% CO_2_ with infection medium to mimic infection conditions. After 72 hours MTT was added and incubated for a further 3 hours. Formazan crystals were dissolved and absorbance read at 570 nm (background 690 nm) on a Biotek Synergy 2 plate reader. Cytotoxicity was assumed when a statistical difference was seen between a drug concentration and untreated controls using a two-way ANOVA (α = 0.05) with Sidak post-hoc tests. Drug concentrations that showed cytotoxicity were excluded from further analysis.

### Data accessibility statement

All data generated or analyzed during this study are included in this published article, the Protein Data Bank (PDB) (and the associated Supplementary Information files).

## Electronic supplementary material

Supplementary Information
