# Supplementary material for: Protein-Structure Assisted Optimization of 4,5-Dihydroxypyrimidine-6-Carboxamide Inhibitors of Influenza Virus Endonuclease
Source: Sci Rep. 2017 Dec 7;7:17139. doi: 10.1038/s41598-017-17419-6 (PMC5719402; doi:10.1038/s41598-017-17419-6)

Supplementary Results

Protein-Structure Assisted Optimization of 4,5-Dihydroxypyrimidine-6-Carboxamide Inhibitors of Influenza Virus Endonuclease

Diane Beylkin,[1,§] Gyanendra Kumar,[2,§] Wei Zhou,[1,§] Jaehyeon Park,[1] Trushar Jeevan,[3] Chandraiah Lagisetti,[1] Rhodri Harfoot,[3] Richard J. Webby,[3] Stephen W. White,[2] and Thomas R. Webb[1,*]

[1]Bioscience Division, SRI International, Menlo Park, CA 94025; [2]Department of Structural Biology and [3]Department of Infectious Diseases, St. Jude Children's Research Hospital, Memphis, TN 38105

[§]These authors contributed equally to this work

Supplementary Figure S1a. Crystal structures of selected carboxylate analogs in complex with the wild type PA$_N$ construct.

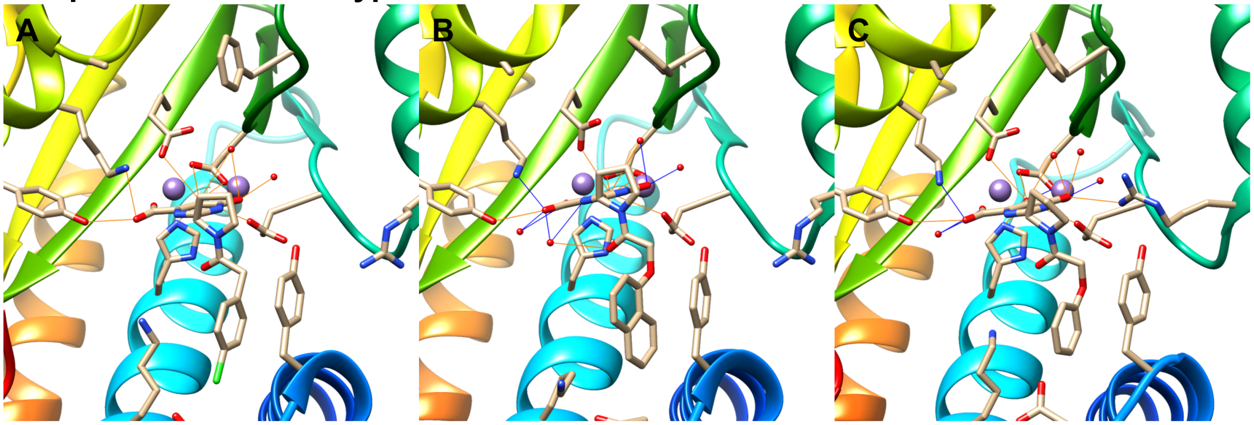

Carboxylate analogs A) **6a**, B) **6b**, and C) **6c** in complex with the wild type PA$_N$ construct. Blue lines show hydrogen-bond interactions and orange lines show interactions outside of standard distances.

Supplementary Figure S1b. Electron density maps for the inhibitors shown in the main text Figure 2.

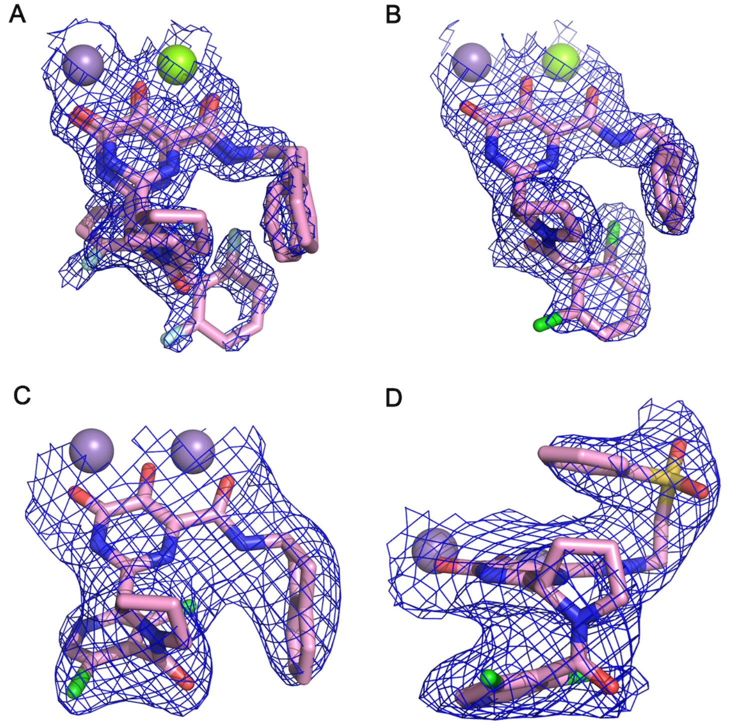

Electron density maps (2Fo-Fc) of the inhibitors bound at the active site of PA_N. (A) Compound 7b at 0.8σ, (B) Compound 7a at 1σ, (C) Compound 8f at 1σ and (D) Compound 8e at 1σ. Mn^{2++} ions are shown as violet balls and Mg^{2++} as green balls, inhibitors are shown as sticks and electron density maps as blue mesh. In case of 7b, the compound adopts cis and trans conformations which explains the week electron density for difluoro-benzene moiety.

Supplementary Figure S1c: Electron density maps for the inhibitors shown in the main text Figure 3.

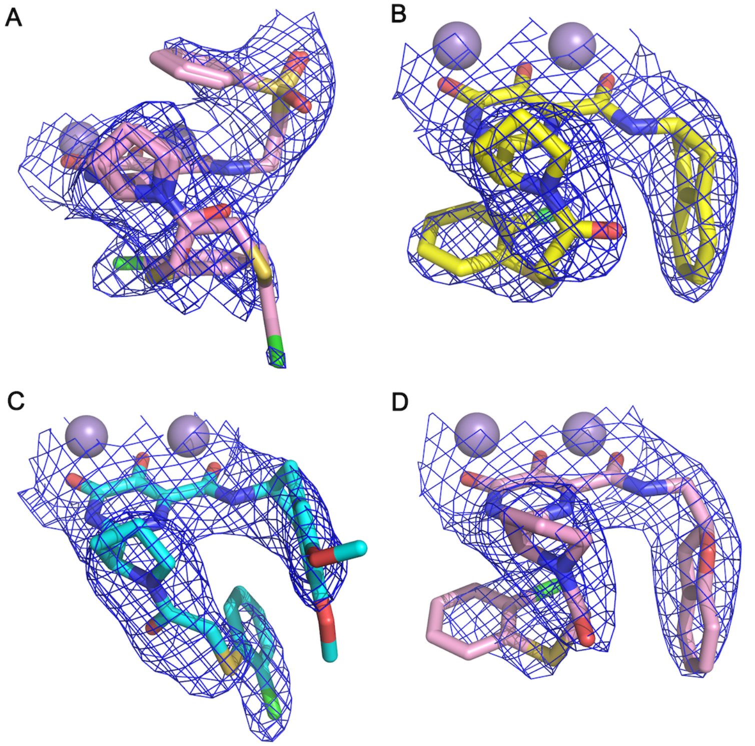

Electron density maps (2Fo-Fc) of the inhibitors bound at the active site of PA_N. (A) Compound 10e at 0.7σ, (B) Compound 10i at 1σ, (C) Compound 10j at 1σ and (D) Compound 10k at 0.9σ. Mn^{2++} ions are shown as violet balls, inhibitors are shown as sticks and electron density maps as blue mesh. In case of 10e, there are two alternative conformations.

Supplementary Figure S1d: Electron density map for the inhibitor 10e shown in the main text Figure 4.

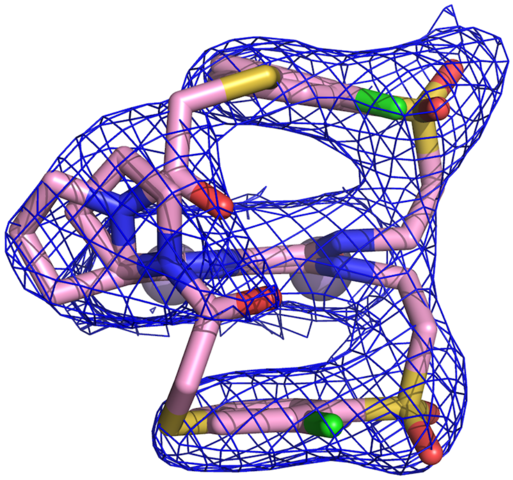

Electron density maps (2Fo-Fc) of the inhibitor 10e bound at the active site of PA_N. Electron density is contoured at 0.9σ. Mn^{2++} ions are shown as violet balls, inhibitors are shown as sticks and electron density maps as blue mesh. There are two alternative conformations of the bound inhibitor.

Supplementary Figure S1e: Electron density map for the inhibitor 10e shown in the main text Figure 5.

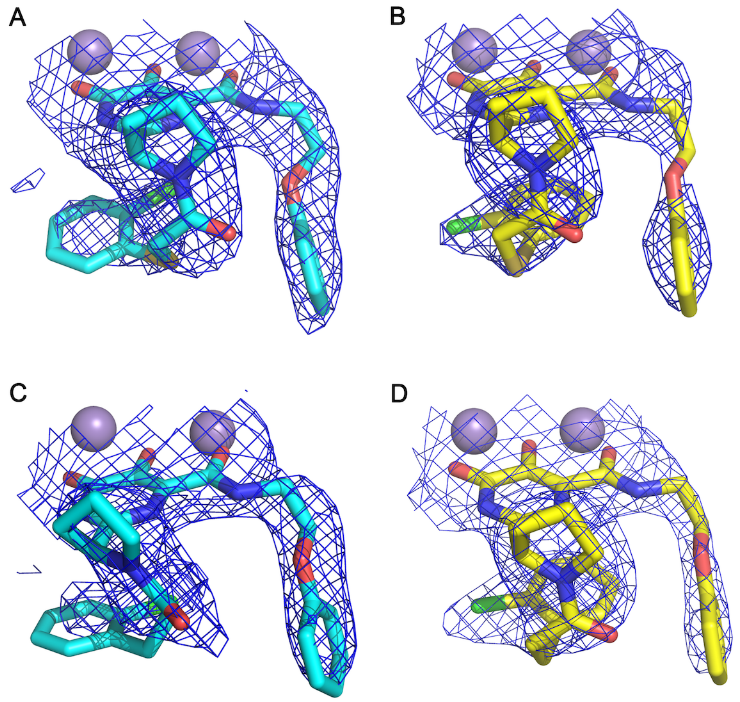

Electron density maps (2Fo-Fc) of the inhibitors bound at the active site of PA_N. (A) Compound 9k with wt (B) Compound 9k with E119D, (C) Compound 9b with wt and (D) Compound 9b with E119D. Mn^{2++} ions are shown as violet balls, inhibitors are shown as sticks and electron density maps as blue mesh contoured at 0.9σ.

Supplementary Figure S2. Plaque Inhibition and Cytotoxicity Data.

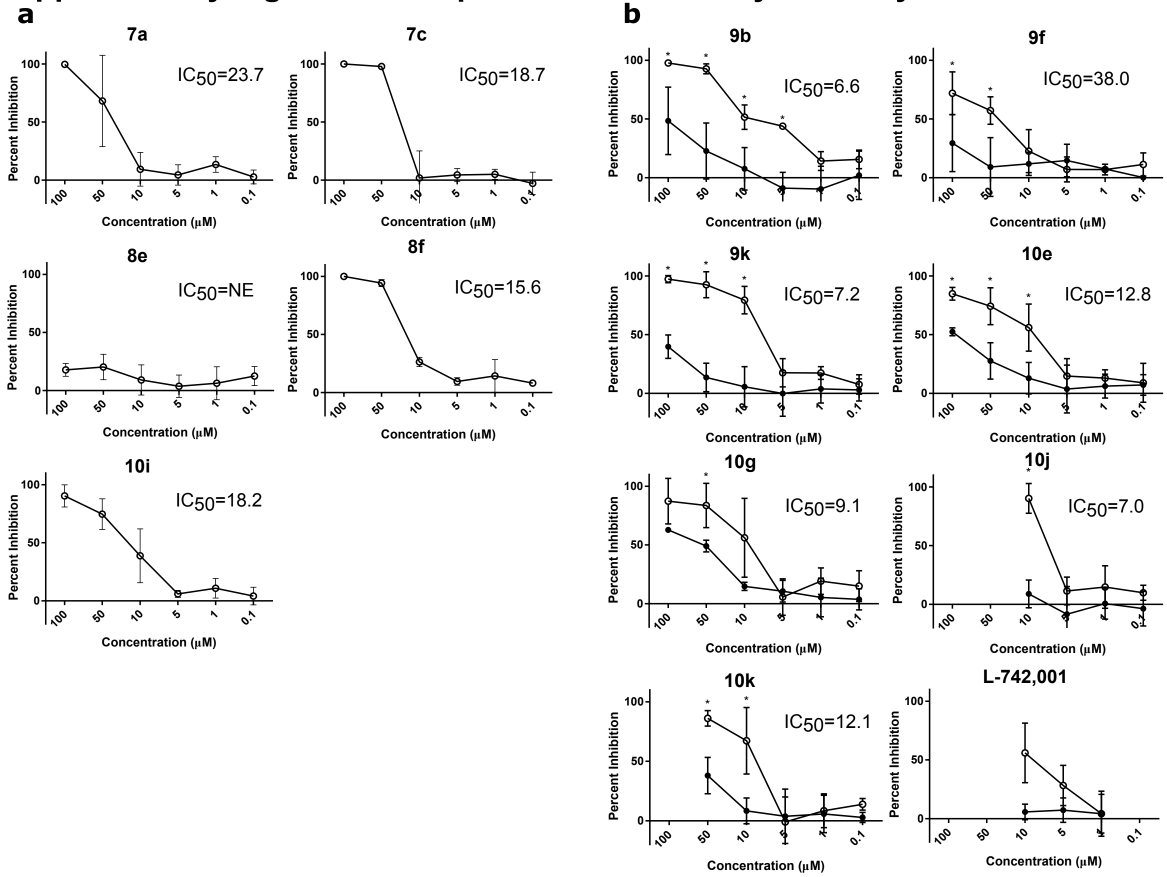

c

Conc (μM)	7a	7c	8e	8f	9b	9f	9k	10e	10g	10i	10j	10k	L-742,001
100	0.011	0.044	0.006	0.083	0.015	0.026	0.011	0.006	0.003	0.006	0.062	0.064	
50	0.031	0.024	0.001	0.010	0.012	0.043	0.022	0.010	0.002	0.041	0.049	0.100	
10	0.083	0.053	0.048	0.462	0.289	0.015	0.004	0.020	0.005	0.031	0.103	0.259	0.810
5	0.031	0.038	0.011	0.293	0.045	0.043	0.028	0.047	0.018	0.020	0.143	0.052	0.513
1	0.011	0.061	0.032	0.091	0.019	0.046	0.023	0.012	0.054	0.011	0.070	0.025	0.621
0.1	0.031	0.050	0.138	0.266	0.014	0.101	0.023	0.099	0.012	0.078	0.087	0.203	

Plaque inhibition assays showing inhibition of virus growth with compounds representative of varying compound classes: A) Inhibition of wild-type virus with 7a, 7c, 8e, 8f and 10i. B) Inhibition of wild-type (open circles) cf. PA mutant E119D (circles) with 9b, 9f, 9k, 10e, 10g, 10j, 10k.. IC_{50} values in micromoles per liter are indicted for the wild-type only; NE = no effect. (*) indicate points where the wild-type and E119D mutant show statistically significant differences by two-way ANOVA ($n = 4$, $\alpha = 0.05$). Error bars represent mean ± SD. C) Cytotoxicity assays showing p-value for the samples relative to untreated control by unpaired t-test with correction for multiple comparisons using the Holm-Sidak method ($n = 3$, $\alpha = 0.05$); (*) indicates samples where there is a significant difference from untreated control.

Supplementary Figure S3. Structures of selected inhibitors in the wild type PA_N.

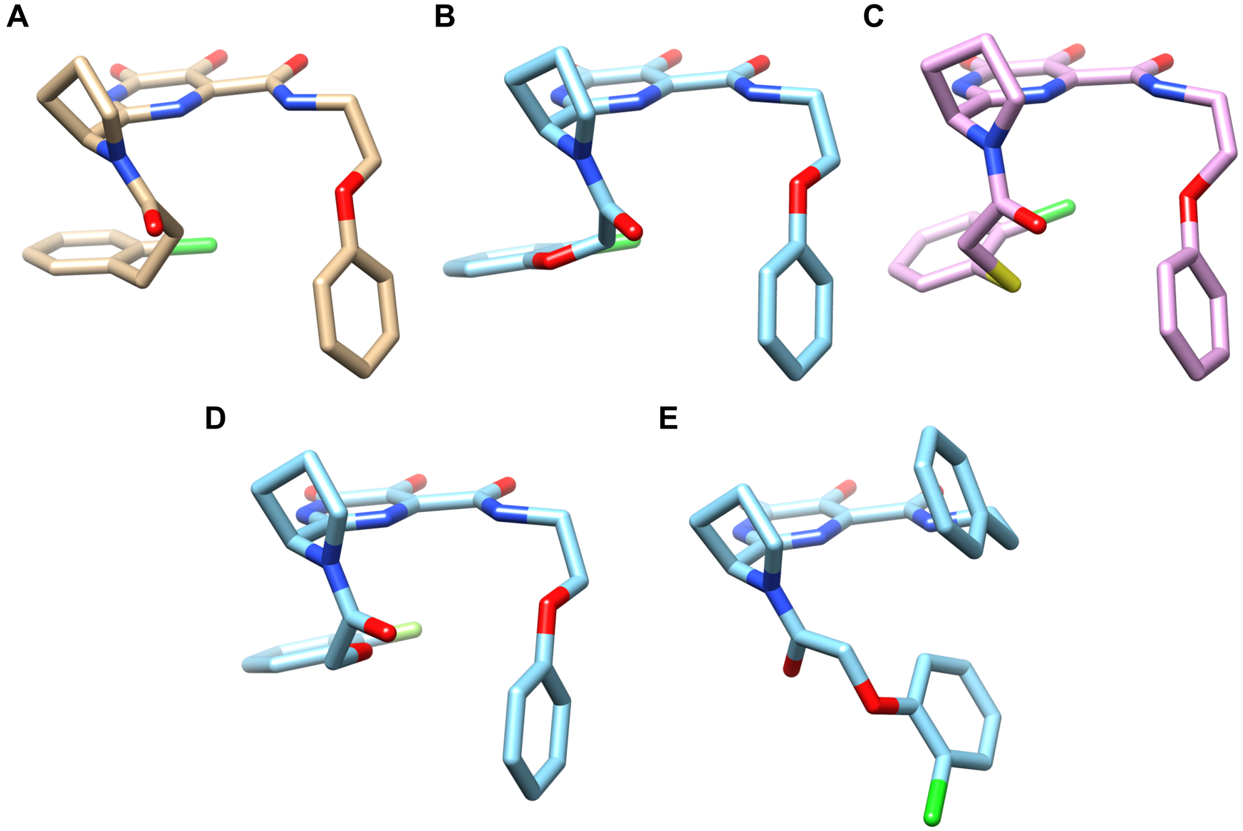

For compounds with X_2 = Cl and R_4 = OPh, such as A) **9b**, B) **9f**, or C) **9k** as well as compounds with X_2 = F and R_4 = OPh, such as D) **9h**, a similar cis conformation is adopted. In this conformation, R_1 stacks underneath the warhead and the phenoxy group stacks with Tyr24. For compounds with R_4 = Ph, such as E) **9f**, a trans conformation is adopted and both the R_1 and R_4 phenyl groups stack with Tyr24.

Supplementary Figure S4. Structures of 8e in the wild type and E119D PA_N constructs.

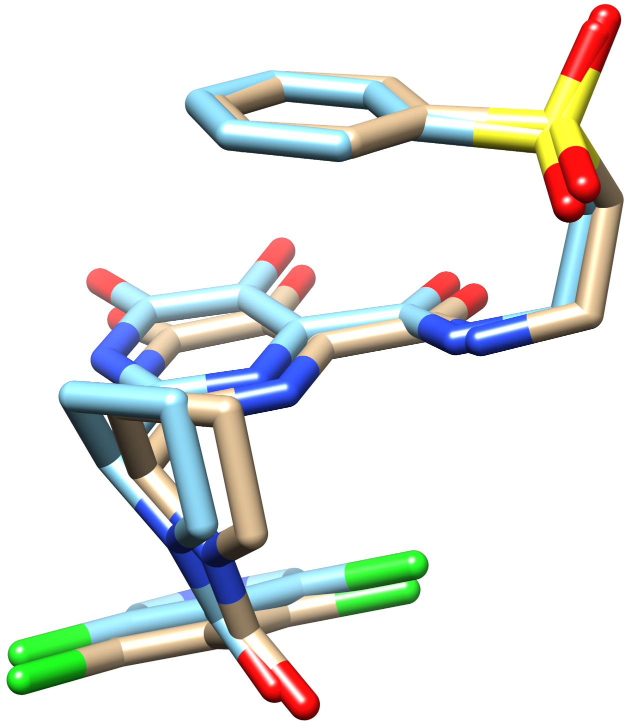

The bound ‘cis-sandwich’ conformation of **8e** with the wild type PA_N construct (in tan), overlaid with the bound conformation observed with the E119D mutant PA_N construct (in cyan). In this case, the inhibitor conformation is similar for wild type and mutant endonucleases.

Supplementary Figure S5. Structures of 10j in the wild type and E119D PA_N constructs.

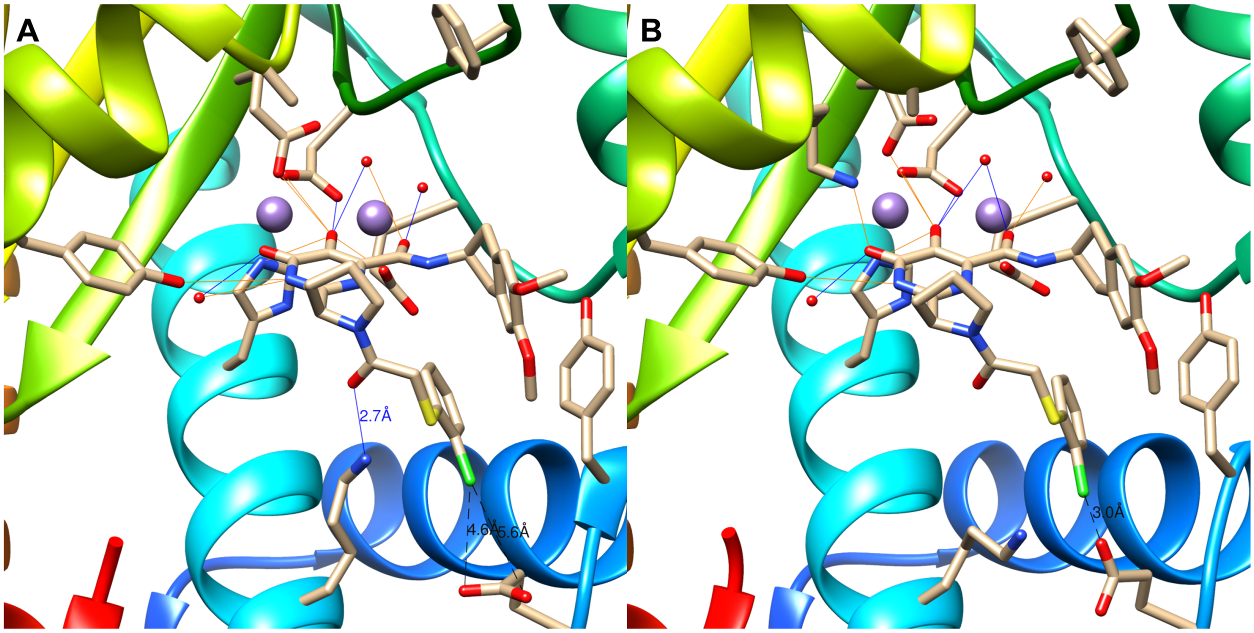

A perspective on the hydrogen bonds and close interactions seen in the co-crystal structure of **10j** in the A) wild type and B) E119D mutant PA_N constructs. Hydrogen-bond interactions are indicated by the blue solid lines, interactions outside of standard distances are indicated by orange solid lines, and other more distant interactions are indicated by black dashed lines. The wild type and mutant structures show significant similarities.

Supplementary Table S1. IC_{50} of representative compounds in FRET based nuclease assay.

Compounds	WT FP Ki (μM)	FRET IC50 (nM)
L-742,001	0.3 ± 0.1	32 ± 2
DPBA	3.7 ± 0.5	98 ± 3
7a	0.36 ± 0.03	36 ± 1
7b	0.06 ± 0.02	26.5 ± 0.2
7c	0.07 ± 0.03	21.9 ± 0.2
8e	0.02 ± 0.02	12 ± 1
8f	0.01 ± 0.01	14.5 ± 0.2
9b	0.4 ± 0.2	29 ± 3
9e	0.4 ± 0.2	37.4 ± 0.8
9k	0.15 ± 0.04	20.5 ± 0.7
10b	0.9 ± 0.2	37 ± 6
10e	0.01 ± 0.04	8.2 ± 0.5
10g	< 0.005	12 ± 1
10i	0.3 ± 0.1	64.4 ± 0.8
10j	0.06 ± 0.05	18 ± 3
10k	0.29 ± 0.05	14 ± 1

Average IC_{50} of selected compounds in FRET based nuclease assay with $PAN^{\Delta Loop}$ was obtained as outlined in methods and reported with respective standard deviations ($n \geq 3$). Average K_is and standard deviation of the selected compounds in FP binding assay with wild type PA protein were included as comparison ($n \geq 3$).

Supplementary Table S2. K_i of representative compounds against E119D mutant in FP binding assay.

Compounds	WT FP Ki (μM)	E119D FP Ki (μM)
L-742,001	3.7 ± 0.5	3 ± 3
7a	0.36 ± 0.03	2 ± 2
7b	0.06 ± 0.02	0.5 ± 0.4
7c	0.07 ± 0.03	0.27 ± 0.09
8e	0.02 ± 0.02	< 0.01
8f	0.01 ± 0.01	0.02 ± 0.04
9b	0.4 ± 0.2	2.6 ± 1.3
9e	0.4 ± 0.2	8 ± 8
9f	0.5 ± 0.2	1 ± 1
9k	0.15 ± 0.04	1.4 ± 0.7
10e	0.01 ± 0.04	0.3 ± 0.5
10g	< 0.01	0.1 ± 0.5
10i	0.3 ± 0.1	9 ± 8
10j	0.06 ± 0.05	0.8 ± 0.4
10k	0.29 ± 0.05	7 ± 9
10m	0.4 ± 0.2	0.6 ± 0.7

Average K_i of selected compounds against E119D-PAN$^{\Delta Loop}$ in FP binding assay was obtained as outlined in methods and reported with respective standard deviations and compared to its average K_i against wild type PA ($n \geq 3$).

Supplementary Table S3. Summary of inhibitor complexes crystallized and crystallization conditions.

Inhibitor	Protein	Crystallization Condition	Space Group	Res. (Å)	PDB Code
6d	WT	0.8 M Succinic Acid pH 7.0, 2 mM $MnCl_2$, 2 mM $MgCl_2$	$P3_221$	2.1	5WDN
6e	WT	0.8 M Succinic Acid pH 7.0, 5 mM $MnCl_2$, 5 mM $MgCl_2$	$P3_221$	1.95	5W3I
7b	WT	0.1 M Tris pH 8.5, 30% PEG 4000, 2 mM $MnCl_2$, 0.2 M $MgCl_2$	$P6_422$	1.85	5WE9
7a	WT	0.1 M Tris pH 8.5, 30% PEG 4000, 2 mM $MnCl_2$, 0.2 M $MgCl_2$	$P6_422$	2.1	5W44
7a	F105S	0.1 M Tris pH 8.5, 30% PEG 4000, 2 mM $MnCl_2$, 0.2 M $MgCl_2$	$P6_422$	2.0	5WEF
7a	E119D	0.1 M CAPSO pH 9.5, 0.8 M Ammonium sulfate, 50 mM $MnCl_2$, 50 mM $MgCl_2$	I422	2.25	5WEI
6c	WT	0.1 M Tris pH 8.5, 24% PEG 6000, 1 M LiCl, 0.1 M Na-Acetate	$P6_422$	2.3	5WCT
	WT	0.8 M Succinic Acid pH 7.0, 1 mM $MnCl_2$, 1 mM $MgCl_2$	$P3_221$	2.12	5WE7
6b	WT	0.1 M Tris pH 8.5, 30% PEG 3000, 2 mM $MnCl_2$, 0.2 M $MgCl_2$	$P6_422$	2.52	5WCS
9f	WT	0.1 M HEPES pH 7.8, 1% PVP K15, 1.0 M Ammonium sulfate, 10 mM $MnCl_2$, 10 mM $MgCl_2$	I422	2.2	5W73
9e	WT	0.1 M HEPES pH 7.8, 1% PVP K15, 1.0 M Ammonium sulfate, 10 mM $MnCl_2$, 10 mM $MgCl_2$	I422	2.1	5WDC
8f	WT	0.1 M CAPSO pH 9.5, 1 M Ammonium sulfate, 10 mM $MnCl_2$, 10 mM $MgCl_2$	I422	2.2	5W7U
9h	WT	0.1 M HEPES pH 7.8, 1% PVP K15, 1.0 M Ammonium sulfate, 10 mM $MnCl_2$, 10 mM $MgCl_2$	I422	2.25	5WA6
9k	WT	0.1 M HEPES pH 7.8, 1% PVP K15, 1.0 M Ammonium sulfate, 10 mM $MnCl_2$, 10 mM $MgCl_2$,	I422	2.1	5W9G
9k	E119D	0.1 M CAPSO pH 9.4, 1 M Ammonium sulfate, 50 mM $MnCl_2$, 50 mM $MgCl_2$	I422	2.3	5WF3
10e	WT	0.1 M HEPES pH 7.8, 1% PVP K15, 1.0 M Ammonium sulfate, 10 mM $MnCl_2$, 10 mM $MgCl_2$,	I422	2.25	5WEB
10e	E119D	0.1 M CAPSO pH 9.4, 1 M Ammonium sulfate, 50 mM $MnCl_2$, 50 mM $MgCl_2$	I422	2.25	5WFM
10i	WT	0.1 M CAPSO pH 9.5,	I422	2.2	5WAP

		1 M Ammonium sulfate,<br>10 mM $MnCl_2$, 10 mM $MgCl_2$			
10j	WT	0.1 M HEPES pH 7.8, 1% PVP K15,<br>1.0 M Ammonium sulfate,<br>10 mM $MnCl_2$, 10 mM $MgCl_2$	I422	2.2	5WB3
10j	E119D	0.1 M CAPSO pH 9.4,<br>1 M Ammonium sulfate,<br>50 mM $MnCl_2$, 50 mM $MgCl_2$	I422	2.3	5WFW
10k	WT	0.1 M CAPSO pH 9.5,<br>1 M Ammonium sulfate,<br>10 mM $MnCl_2$, 10 mM $MgCl_2$	I422	2.2	5WDW
8e	WT	0.1 M HEPES pH 7.8, 1% PVP K15,<br>1.0 M Ammonium sulfate,<br>10 mM $MnCl_2$, 10 mM $MgCl_2$,	I422	2.3	5W92
8e	E119D	0.1 M CAPSO pH 9.6,<br>1.1 M Ammonium sulfate,<br>50 mM $MnCl_2$, 50 mM $MgCl_2$	I422	2.35	5WFZ
9b	WT	0.1 M HEPES pH 7.8, 1% PVP K15,<br>1.0 M Ammonium sulfate,<br>10 mM $MnCl_2$, 10 mM $MgCl_2$,	I422	2.2	5WA7
9b	E119D	0.1 M CAPSO pH 9.4,<br>1 M Ammonium sulfate,<br>50 mM $MnCl_2$, 50 mM $MgCl_2$	I422	2.3	5WG9

Supplementary Table S4. X-ray data collection and refinement statistics.

	PA_N-(6d) (PDB ID: 5WDN)	PA_N-(6e) (PDB ID: 5W3I)	PA_N-(7b) (PDB ID: 5WE9)
**Data collection**			
Space group	$P3_221$	$P3_221$	$P6_422$
Cell dimensions			
a, *b*, *c* (Å)	60.168 60.168 94.443	61.162 61.162 94.813	74.081 74.081 127.694
α, β, γ (°)	90 90 120	90 90 120	90 90 120
Resolution (Å)	50 - 2.1 (2.17 - 2.1)[a]	50 - 1.95 (2.02 - 1.95)	50 - 1.85 (1.92 - 1.85)
$R_{meas,}$	0.082 (0.713)	0.07 (1.003)	0.067 (0.922)
$I/\sigma(I)$	31.2 (4.3)	31.4 (2.8)	42.5 (2.4)
Completeness (%)	99.64 (100.00)	100.0 (100.0)	99.9 (99.7)
Redundancy	10.7 (10.7)	11.2 (10.3)	17.5 (11.9)
**Refinement**			
Resolution (Å)	34.99 - 2.1 (2.17 - 2.1)	35.32 - 1.95 (2.02 - 1.95)	35.57 - 1.85 (1.94 - 1.85)
No. reflections	11985 (1170)	15150 (1205)	18206 (1108)
R_{work} / R_{free}	0.2168 / 0.2321	0.1753 / 0.2011	0.1794 / 0.2066
No. atoms	1499	1541	1621
Protein	1434	1423	1445
Ligand/ion	28	38	75
Water	37	80	101
B factors			
Protein	67.58	24.42	45.82
Ligand/ion	65.62	34.05	40.07
Water	60.43	32.78	52.73
R.m.s. deviations			
Bond lengths (Å)	0.002	0.005	0.010
Bond angles (°)	0.53	0.73	1.01

[a] Values in parentheses are for highest-resolution shell.

Supplementary Table S5. X-ray data collection and refinement statistics.

	PA_N-(7a) (PDB ID: 5W44)	PA_NF105S-(7a) (PDB ID: 5WEF)	PA_NE119D-(7a) (PDB ID: 5WEI)
**Data collection**			
Space group	$P6_422$	$P6_422$	I422
Cell dimensions			
a, *b*, *c* (Å)	74.111 74.111 127.845	73.878 73.878 127.282	89.474 89.474 133.662
α, β, γ (°)	90 90 120	90 90 120	90 90 90
Resolution (Å)	50 - 2.10 (2.18 - 2.10)[a]	50 - 1.99 (2.06 - 1.99)	50 - 2.25 (2.33 - 2.25)
$R_{meas,}$	0.097 (0.650)	0.094 (1.000)	0.105 (1.000)
$I/\sigma(I)$	35.5 (3.2)	43.6 (1.9)	29.4 (1.2)
Completeness (%)	99.7 (97.6)	99.9 (100.0)	99.6 (97.5)
Redundancy	16.5 (11.6)	13.8 (13.7)	8.6 (5.9)
**Refinement**			
Resolution (Å)	42.61 - 2.10 (2.17 - 2.10)	31.95 - 1.99 (2.06 - 1.99)	37.18 - 2.25 (2.33 - 2.25)
No. reflections	12679 (1184)	14686 (1382)	13216 (1241)
R_{work} / R_{free}	0.1871 / 0.2283	0.1940 / 0.2269	0.1965 / 0.2210
No. atoms	1541	1529	1466
Protein	1443	1420	1412
Ligand/ion	37	49	38
Water	61	60	16
B factors			
Protein	51.88	62.24	77.92
Ligand/ion	52.96	71.31	120.54
Water	50.70	60.73	77.22
R.m.s. deviations			
Bond lengths (Å)	0.009	0.017	0.004
Bond angles (°)	0.84	1.34	0.60

[a] Values in parentheses are for highest-resolution shell.

Supplementary Table S6. X-ray data collection and refinement statistics.

	PA_N-(6c) (PDB ID: 5WCT)	PA_N-(6b) (PDB ID: 5WCS)
**Data collection**		
Space group	$P6_422$	$P6_422$
Cell dimensions		
a, *b*, *c* (Å)	74.561 74.561 125.062	74.666 74.666 125.722
α, *β*, *γ* (°)	90 90 120	90 90 120
Resolution (Å)	50 - 2.30 (2.38 - 2.30)[a]	50 - 2.53 (2.62 - 2.53)
$R_{meas,}$	0.111 (1.024)	0.125 (1.096)
$I/\sigma(I)$	37.8 (2.1)	35.3 (2.4)
Completeness (%)	97.6 (88.7)	99.7 (97.6)
Redundancy	16.5 (15)	21.4 (15.3)
**Refinement**		
Resolution (Å)	35.02 - 2.30 (2.38 - 2.30)	35.17 - 2.53 (2.62 - 2.53)
No. reflections	9475 (785)	7447 (696)
R_{work} / R_{free}	0.2188 / 0.2549	0.2063 / 0.2402
No. atoms	1445	1495
Protein	1401	1440
Ligand/ion	28	32
Water	16	23
B factors		
Protein	87.24	71.74
Ligand/ion	72.20	104.17
Water	67.75	58.82
R.m.s. deviations		
Bond lengths (Å)	0.008	0.002
Bond angles (°)	0.89	0.42

[a] Values in parentheses are for highest-resolution shell.

Supplementary Table S7. X-ray data collection and refinement statistics.

	PA_N-(9f) (PDB ID: 5W73)	PA_N-(9e) (PDB ID: 5WDC)	PA_N-(8f) (PDB ID: 5W7U)
**Data collection**			
Space group	I422	I422	I422
Cell dimensions			
a, b, c (Å)	89.843 89.843 135.002	90.777 90.777 134.545	90.746 90.746 135.256
α, β, γ (°)	90 90 90	90 90 90	90 90 90
Resolution (Å)	50 - 2.20 (2.28 - 2.20)[a]	50 – 2.10 (2.18 - 2.10)	50 - 2.20 (2.28 - 2.20)
$R_{meas,}$	0.061 (1.019)	0.063 (1.067)	0.054 (0.760)
$I/\sigma(I)$	36.8 (1.5)	34.9 (1.4)	40.7 (1.8)
Completeness (%)	99.9 (98.9)	99.1 (93.0)	98.89 (92.41)
Redundancy	7.2 (6.6)	7.7 (6.4)	9.3 (6.9)
**Refinement**			
Resolution (Å)	40.24 - 2.20 (2.28 - 2.20)	37.63 – 2.10 (2.18 - 2.10)	32.08 - 2.20 (2.28 - 2.20)
No. reflections	14360 (1387)	16680 (1546)	14581 (1328)
R_{work} / R_{free}	0.2225 / 0.2488	0.2182 / 0.2422	0.2185 / 0.2443
No. atoms	1477	1468	1447
Protein	1417	1404	1399
Ligand/ion	48	47	37
Water	12	17	11
B factors			
Protein	82.17	78.69	89.82
Ligand/ion	101.27	95.20	99.46
Water	71.72	76.85	77.48
R.m.s. deviations			
Bond lengths (Å)	0.004	0.004	0.008
Bond angles (°)	0.54	0.57	0.91

[a] Values in parentheses are for highest-resolution shell.

Supplementary Table S8. X-ray data collection and refinement statistics.

	PA_N-(9h) (PDB ID: 5WA6)	PA_N-(9k) (PDB ID: 5W9G)	PA_N-(10e) (PDB ID: 5WEB)
**Data collection**			
Space group	I422	I422	I422
Cell dimensions			
a, *b*, *c* (Å)	90.563 90.563 135.167	90.249 90.249 135.307	90.793 90.793 135.062
α, β, γ (°)	90 90 90	90 90 90	90 90 90
Resolution (Å)	50 - 2.25 (2.33 - 2.25)[a]	50 – 2.10 (2.18 - 2.10)	50 - 2.25 (2.33 - 2.25)
$R_{meas,}$	0.071 (1.215)	0.061 (1.092)	0.064 (1.198)
$I/\sigma(I)$	36.0 (1.5)	38.3 (1.5)	36.5 (1.6)
Completeness (%)	99.9 (99.4)	99.4 (95.6)	99.8 (99.8)
Redundancy	7.8 (7.0)	7.5 (6.7)	7.0 (6.8)
**Refinement**			
Resolution (Å)	38.8 - 2.25 (2.33 - 2.25)	38.68 – 2.10 (2.17 - 2.10)	38.88 - 2.25 (2.33 - 2.25)
No. reflections	13738 (1355)	16669 (1556)	13680 (1342)
R_{work} / R_{free}	0.2307 / 0.2535	0.2249 / 0.2409	0.2192 / 0.2546
No. atoms	1453	1450	1513
Protein	1407	1389	1409
Ligand/ion	38	44	88
Water	8	17	16
B factors			
Protein	91.32	84.76	89.81
Ligand/ion	102.91	106.49	98.40
Water	82.39	75.48	89.53
R.m.s. deviations			
Bond lengths (Å)	0.003	0.003	0.008
Bond angles (°)	0.61	0.59	0.90

[a] Values in parentheses are for highest-resolution shell.

Supplementary Table S9. X-ray data collection and refinement statistics.

	PA_N-(10i) (PDB ID: 5WAP)	PA_N-(10j) (PDB ID: 5WB3)	PA_N-(10k) (PDB ID: 5WDW)
**Data collection**			
Space group	I422	I422	I422
Cell dimensions			
a, *b*, *c* (Å)	90.653 90.653 135.17	90.698 90.698 134.31	90.744 90.744 135.876
α, *β*, *γ* (°)	90 90 90	90 90 90	90 90 90
Resolution (Å)	50 - 2.20 (2.28 - 2.20)[a]	50 - 2.20 (2.28 - 2.20)	50 - 2.30 (2.38 - 2.30)
$R_{meas,}$	0.079 (0.879)	0.068 (1.148)	0.058 (0.810)
$I/\sigma(I)$	31.3 (1.7)	35.8 (2.0)	35.5 (2.0)
Completeness (%)	99.7 (97.5)	100.0 (100.0)	99.3 (98.4)
Redundancy	8.4 (6.7)	7.8 (7.8)	9.4 (7.8)
**Refinement**			
Resolution (Å)	32.05 - 2.20 (2.28 - 2.20)	30.06 - 2.20 (2.28 - 2.20)	32.08 - 2.30 (2.38 - 2.30)
No. reflections	14617 (1389)	14543 (1410)	12940 (1240)
R_{work} / R_{free}	0.2260 / 0.2447	0.2152 / 0.2417	0.2401 / 0.2854
No. atoms	1457	1478	1424
Protein	1404	1410	1381
Ligand/ion	43	52	39
Water	10	16	4
B factors			
Protein	82.80	77.31	88.12
Ligand/ion	90.80	95.14	99.65
Water	77.53	71.53	71.29
R.m.s. deviations			
Bond lengths (Å)	0.002	0.004	0.003
Bond angles (°)	0.47	0.58	0.51

[a] Values in parentheses are for highest-resolution shell.

Supplementary Table S10. X-ray data collection and refinement statistics.

	PA_N-(8e) (PDB ID: 5W92)	PA_N-(9b) (PDB ID: 5WA7)
**Data collection**		
Space group	I422	I422
Cell dimensions		
a, *b*, *c* (Å)	90.448 90.448 134.167	90.298 90.298 135.708
α, β, γ (°)	90 90 90	90 90 90
Resolution (Å)	50 - 2.30 (2.38 - 2.30)[a]	50 - 2.20 (2.28 - 2.20)
$R_{meas,}$	0.1 (1.248)	0.068 (1.512)
$I/\sigma(I)$	32.0 (1.7)	32.7 (1.3)
Completeness (%)	99.9 (100.0)	99.3 (98.9)
Redundancy	7.4 (7.5)	7.0 (6.7)
**Refinement**		
Resolution (Å)	38.73 - 2.30 (2.38 - 2.30)	40.44 - 2.20 (2.28 - 2.20)
No. reflections	12733 (1262)	14445 (1391)
R_{work} / R_{free}	0.2122 / 0.2574	0.2319 / 0.2494
No. atoms	1496	1455
Protein	1402	1401
Ligand/ion	78	43
Water	16	11
B factors		
Protein	80.26	89.87
Ligand/ion	111.46	130.24
Water	74.60	80.03
R.m.s. deviations		
Bond lengths (Å)	0.007	0.003
Bond angles (°)	0.76	0.53

[a] Values in parentheses are for highest-resolution shell.

Supplementary Table S11. X-ray data collection and refinement statistics.

	PA_NE119D-(9k) (PDB ID: 5WF3)	PA_NE119D-(10e) (PDB ID: 5WFM)	PA_NE119D-(10j) (PDB ID: 5WFW)
**Data collection**			
Space group	I422	I422	I422
Cell dimensions			
a, *b*, *c* (Å)	90.679 90.679 133.752	90.902 90.902 133.737	90.425 90.425 133.813
α, β, γ (°)	90 90 90	90 90 90	90 90 90
Resolution (Å)	50 - 2.25 (2.33 - 2.25)[a]	50 - 2.25 (2.33 - 2.25)	50 - 2.29 (2.37 - 2.29)
$R_{meas,}$	0.136 (0.809)	0.083 (1.000)	0.094 (1.000)
$I/\sigma(I)$	25.0 (1.8)	30.5 (2.0)	29.9 (2.1)
Completeness (%)	98.6 (88.2)	99.9 (99.9)	99.9 (100.0)
Redundancy	9.5 (4.9)	10.7 (8.9)	10.0 (8.0)
**Refinement**			
Resolution (Å)	32.06 - 2.25 (2.33 - 2.25)	37.59 - 2.25 (2.33 - 2.25)	29.96 - 2.29 (2.37 - 2.29)
No. reflections	13412 (1170)	13661 (1344)	12811 (1243)
R_{work} / R_{free}	0.2037 / 0.2270	0.2048 / 0.2254	0.2127 / 0.2235
No. atoms	1460	1557	1491
Protein	1402	1439	1422
Ligand/ion	48	88	52
Water	10	30	17
B factors			
Protein	72.51	70.05	77.03
Ligand/ion	102.86	77.67	101.7
Water	69.77	69.10	76.78
R.m.s. deviations			
Bond lengths (Å)	0.006	0.002	0.004
Bond angles (°)	0.59	0.47	0.61

[a] Values in parentheses are for highest-resolution shell.

Supplementary Table S12. X-ray data collection and refinement statistics.

	PA_NE119D-(8e) (PDB ID: 5WFZ)	PA_NE119D-(9b) (PDB ID: 5WG9)
**Data collection**		
Space group	I422	I422
Cell dimensions		
a, b, c (Å)	90.3 90.3 133.353	90.612 90.612 133.788
α, β, γ (°)	90 90 90	90 90 90
Resolution (Å)	50 - 2.35 (2.43 - 2.35)[a]	50 - 2.29 (2.37 - 2.29)
$R_{meas,}$	0.093 (0.986)	0.103 (0.776)
$I/\sigma(I)$	21.8 (1.7)	25.0 (2.0)
Completeness (%)	99.9 (99.9)	98.73 (90.40)
Redundancy	6.9 (6.5)	8.7 (4.5)
**Refinement**		
Resolution (Å)	31.93 - 2.35 (2.43 - 2.35)	29.99 - 2.29 (2.37 - 2.29)
No. reflections	11859 (1170)	12714 (1149)
R_{work} / R_{free}	0.2072 / 0.2270	0.1983 / 0.2207
No. atoms	1511	1468
Protein	1426	1401
Ligand/ion	55	48
Water	30	19
B factors		
Protein	63.62	74.73
Ligand/ion	109.66	95.91
Water	63.81	77.62
R.m.s. deviations		
Bond lengths (Å)	0.002	0.006
Bond angles (°)	0.42	0.84

[a] Values in parentheses are for highest-resolution shell.

Synthesis of Library Compounds:

General Remarks. Reagents were obtained from Sigma Aldrich, Oakwood Chemical, TCI America, AK Scientific, Enamine, Life Chemicals, Ark Pharm, J + W Pharm Lab, Acros, or Combi-Blocks at the highest grade available and were used as purchased. Automated flash chromatography purifications were conducted using Biotage SNAP cartridges for normal phase purification and Biotage KP-C18-HS SNAP cartridges for reverse phase purifications on Biotage SP-4 or Isolera Four systems. NMR experiments were conducted using a 400 MHz Varian instrument. Compounds tested were of >95% purity (average based on UV detection at 280 nm) as determined by high pressure liquid chromatography (HPLC) conducted on an XBridge C18 5 µm 4.6 x 250 mm column with gradient elution using 0.1% formic acid in water/acetonitrile mobile phase. LCMS data was obtained on an LCQ Fleet Ion Trap Mass Spectrometer (Thermo Scientific) coupled to the Finnigan Surveyor Plus HPLC System (Thermo Scientific).

Supplementary Scheme 1. Synthesis of dimethyl 2-((((Z)-amino((S)-1-((benzyloxy)carbonyl)pyrrolidin-2-yl)methylene)amino)oxy)maleate.

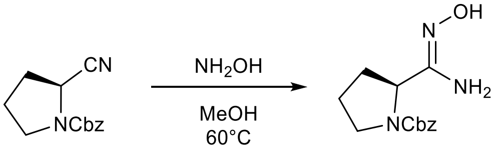

(S,E)-benzyl 2-(N'-hydroxycarbamimidoyl)pyrrolidine-1-carboxylate. To a solution of *Z*-L-prolinamide (2.8 g, 12 mmol, 1 eq.) in methanol (25 mL) at room temperature was added a solution of hydroxylamine (50 wt. % in H_2O, 0.74 mL, 12 mmol, 1 eq.). The reaction mixture was stirred at 60^0C for 3 h then concentrated *in vacuo* to obtain a white solid (3.0 g, 95%). ^{1}H NMR (400 MHz, DMSO-d_6): δ ppm = 9.03 (d, J = 18.8 Hz, 1H), 7.50 – 7.21 (m, 6H), 5.48 – 5.23 (m, 2H), 5.06 (d, J = 9.5 Hz, 2H), 4.39 – 4.20 (m, 1H), 3.43 (d, J = 7.5 Hz, 1H), 3.34 (d, J = 5.6 Hz, 2H), 2.01 – 1.71 (m, 4H). LCMS (ESI): m/z = 263 $[M + H]^+$.

Supplementary Scheme 2. Synthesis of methyl (S)-2-(1-((benzyloxy)carbonyl)pyrrolidin-2-yl)-6-oxo-5-(pivaloyloxy)-1,6-dihydropyrimidine-4-carboxylate.

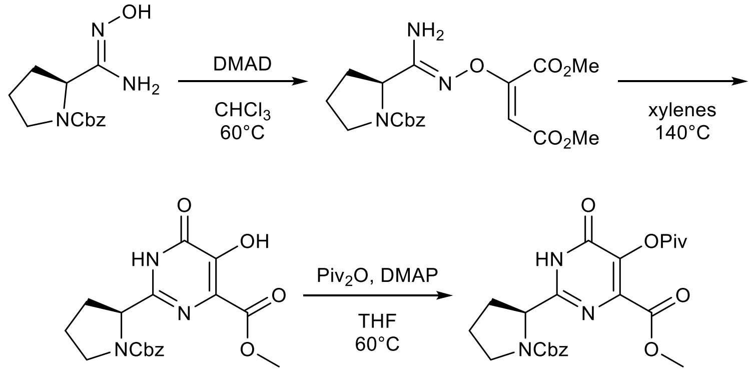

methyl (S)-2-(1-((benzyloxy)carbonyl)pyrrolidin-2-yl)-6-oxo-5-(pivaloyloxy)-1,6-dihydropyrimidine-4-carboxylate. To a suspension of (S,E)-benzyl 2-(N'-hydroxycarbamimidoyl)pyrrolidine-1-carboxylate (1.1 g, 4.1 mmol, 1 eq.) in methanol (10 mL) at room temperature was added dimethyl acetylene dicarboxylate (0.50 mL, 4.1 mmol, 1 eq.). The reaction mixture was stirred at 60^0C under N_2 for 3 h, then cooled to room temperature and concentrated *in vacuo*. The crude product was resuspended in

xylenes (10 mL) and stirred at 140^{0}C for 16 h. After cooling to room temperature the reaction mixture was cooled to room temperature, concentrated *in vacuo* and the crude material was used without purification.

The crude material was diluted in anhydrous THF (5 mL) at room temperature and 4-dimethylaminopyridine (0.043 g, 0.35 mmol, 0.1 eq.) was added, followed by pivalic anhydride (0.88 mL, 4.4 mmol, 1.1 eq.). The reaction mixture was stirred at 60^{0}C under N_2 for 2 h, then concentrated *in vacuo*. Purification by automated flash chromatography (*n*-hexane/EtOAc) gave an orange foam (0.60 g, 38%).^{1}H NMR (400 MHz, Methanol-*d$_4$*): δ ppm = 7.31 – 7.19 (m, 2H), 7.14 (dd, *J* = 5.1, 1.9 Hz, 2H), 7.00 (dd, *J* = 6.7, 2.9 Hz, 1H), 5.03 (s, 1H), 4.66 – 4.53 (m, 1H), 3.77 (d, *J* = 5.0 Hz, 3H), 3.66 – 3.55 (m, 1H), 3.52 – 3.41 (m, 1H), 2.38 – 2.21 (m, 1H), 2.04 – 1.77 (m, 3H), 1.27 (s, 9H). LCMS (ESI): *m/z* = 457 [M + H]$^+$.

Supplementary Scheme 3. Synthesis of (S)-methyl 6-oxo-5-(pivaloyloxy)-2-(pyrrolidin-2-yl)-1,6-dihydropyrimidine-4-carboxylate hydrochloride

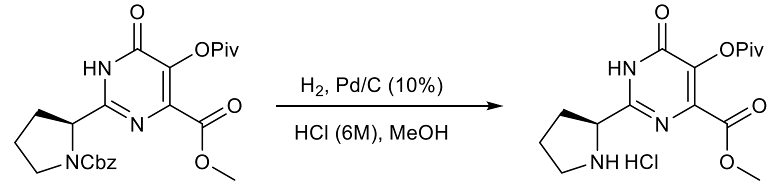

(S)-methyl 6-oxo-5-(pivaloyloxy)-2-(pyrrolidin-2-yl)-1,6-dihydropyrimidine-4-carboxylate hydrochloride. To a solution of (S)-methyl 2-(1-((benzyloxy)carbonyl)pyrrolidin-2-yl)-6-oxo-5-(pivaloyloxy)-1,6-dihydropyrimidine-4-carboxylate (1.5 g, 3.2 mmol, 1 eq.) in methanol (10 mL) was added 10% wetted Pd/C (0.34 g, 1 eq.) and 6M HCl (0.54 mL, 1 eq.). The reaction mixture was placed under a balloon of H_2 and stirred at room temperature for 2 h. The reaction mixture was then diluted with methanol and ethyl acetate and filtered through a pad of celite. The filtrate was concentrated *in vacuo* to obtain a red oil (1.0 g, 96%). ^{1}H NMR (400 MHz, Methanol-*d$_4$*): δ ppm = 4.68 (d, *J* = 7.9 Hz, 1H), 3.93 (s, 3H), 3.58 (td, *J* = 11.3, 10.5, 5.4 Hz, 1H), 3.45 (dt, *J* = 11.2, 6.7 Hz, 1H), 2.57 (d, *J* = 7.3 Hz, 1H), 2.17 (t, *J* = 3.4 Hz, 3H), 1.38 (s, 9H). LCMS (ESI): *m/z* = 323 [M + H]$^+$.

Supplementary Scheme 4. General Procedure for Synthesis of Library compounds: Part 1.

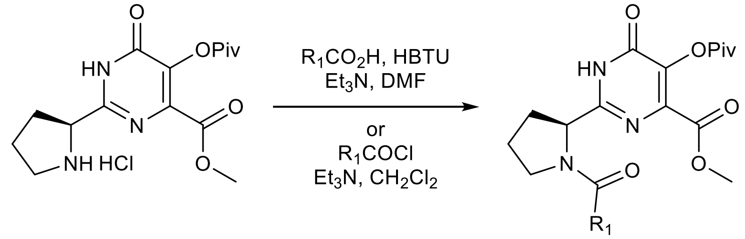

To a mixture of carboxylic acid (1 eq.) in dry DMF (1 mL) at room temperature was added triethylamine (2 eq.), followed by HBTU (1.1 eq.) and amine (1.1 eq), OR to amine (1 eq.) in dry DCM (1 mL) was added acid chloride (1 eq.) followed by triethylamine (3 eq.). Once the reaction was complete, quenched with aqueous NH_4Cl and diluted with ethyl acetate. The organic layer was washed with water and brine, dried over $MgSO_4$, filtered, and concentrated. The crude product was used directly in the next step.

Supplementary Scheme 5. General Procedure for Synthesis of Library compounds: Part II.

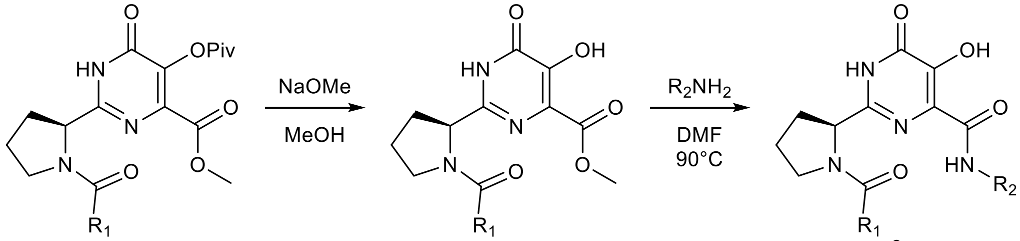

6

To the crude product in MeOH (0.5 M) was added NaOMe (2 eq., 25 wt % solution) dropwise. The reaction stirred at room temperature overnight. When the reaction was complete, it was quenched with a few drops of NH_4Cl and concentrated. The crude product was purified by reverse phase chromatography (MeOH/water + 0.1% formic acid).

To the ester (1 eq.) in dry DMF (0.2 M) was added the amine (3 eq.). This mixture was heated to 90^{0}C overnight. Once the reaction was complete, it was cooled to room temperature and purified by reverse phase column directly (MeOH/water + 0.1% formic acid).

Supplementary Scheme 6. Synthesis of Carboxylate Analogs

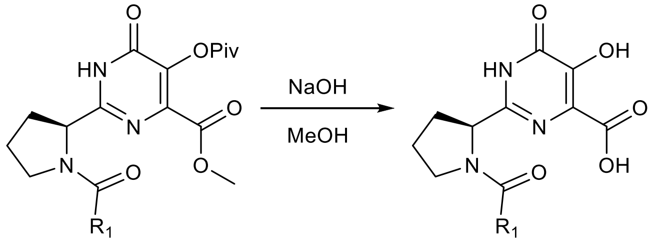

The crude intermediate was dissolved in MeOH (0.5 mL) and 2M sodium hydroxide solution (8 eq.) was added at room temperature. The reaction mixture was then stirred at 60^{0}C for 3-5 h. After cooling to room temperature, the reaction mixture was concentrated to remove methanol. The pH of the reaction mixture was adjusted to 2 using 10% aqueous HCl solution. Purification was achieved using automated reversed-phase flash chromatography (MeCN/water + 1% formic acid).

Library Compounds:

(S)-5-hydroxy-2-(1-(2-(naphthalen-1-yloxy)acetyl)pyrrolidin-2-yl)-6-oxo-1,6-dihydropyrimidine-4-carboxylic acid (6b): ^{1}H NMR (400 MHz, DMSO-d_6): δ ppm = 8.30 – 8.12 (m, 1H), 7.97 – 7.79 (m, 1H), 7.59 – 7.41 (m, 2H), 7.36 (t, J = 8.0 Hz, 1H), 6.92 (d, J = 7.7 Hz, 1H), 5.14 – 4.87 (m, 2H), 4.79 (dd, J = 8.1, 5.0 Hz, 1H), 3.84 (q, J = 7.5 Hz, 1H), 3.65 (q, J = 7.7, 7.2 Hz, 1H), 2.33 – 2.16 (m, 1H), 2.16 – 2.03 (m, 1H), 1.95 (tq, J = 11.4, 5.8 Hz, 1H). LCMS (ESI): m/z = 410 $[M + H]^+$.

(S)-2-(1-(2-(4-chlorophenyl)acetyl)pyrrolidin-2-yl)-5-hydroxy-6-oxo-1,6-dihydropyrimidine-4-carboxylic acid (6c): ^{1}H NMR (400 MHz, DMSO-d_6): δ ppm = 7.37-7.32 (m, 2H), 7.32-7.25 (m, 2H), 4.74 (dd, J = 8.3, 4.4 Hz, 1H), 3.80 – 3.53 (m, 4H), 2.19 (dt, J = 8.7, 4.0 Hz, 1H), 2.07-1.96 (m, 1H), 1.91 (dq, J = 10.3, 5.5 Hz, 2H), 1.12 (s, 1H). LCMS (ESI): m/z = 378 $[M + H]^+$.

(S)-5-hydroxy-6-oxo-2-(1-(2-phenoxyacetyl)pyrrolidin-2-yl)-1,6-dihydropyrimidine-4-carboxylic acid (6d): ^{1}H NMR (400 MHz, Methanol-d_4): δ ppm = δ 7.15 (t, *J* = 7.9 Hz, 2H), 6.89 – 6.77 (m, 2H), 4.71 (s, 2H), 3.78 (s, 1H), 3.64 – 3.47 (m, 1H), 2.21 (t, *J* = 7.1 Hz, 1H), 2.17 – 2.04 (m, 1H), 2.04 – 1.90 (m, 2H). LCMS (ESI): *m/z* = 360 $[M + H]^+$.

(S)-5-hydroxy-N-(2-methoxyethyl)-6-oxo-2-(1-(2-phenoxyacetyl)pyrrolidin-2-yl)-1,6-dihydropyrimidine-4-carboxamide (6e): ^{1}H NMR (400 MHz, Chloroform-*d*): δ ppm = 12.23 (br s, 1H), 7.95 – 7.85 (m, 1H), 7.26 (d, *J* = 1.1 Hz, 2H), 7.03 – 6.84 (m, 3H), 5.04 (dd, *J* = 7.7, 3.6 Hz, 1H), 4.86 – 4.74 (m, 2H), 4.00 – 3.89 (m, 1H), 3.73 – 3.48 (m, 5H), 3.40 (s, 3H), 2.45 – 2.32 (m, 1H), 2.26 – 1.99 (m, 3H). LCMS (ESI): *m/z* = 417 $[M + H]^+$.

(S)-2-(1-(2,6-dichlorobenzoyl)pyrrolidin-2-yl)-5-hydroxy-6-oxo-N-phenethyl-1,6-dihydropyrimidine-4-carboxamide (7a): ^{1}H NMR (400 MHz, Chloroform-*d*): δ ppm = 12.29 (s, 1H), 11.07 (s, 1H), 7.53 – 7.46 (m, 1H), 7.37 - 7.25 (m, 5H), 7.25 - 7.16 (m, 2H), 5.19 (dd, *J* = 8.0, 2.6 Hz, 1H), 3.72 - 3.64 (m, 2H), 3.28 - 3.19 (m, 2H), 2.91 (t, *J* = 6.8 Hz, 2H), 2.56 - 2.45 (m, 1H), 2.15 - 2.08 (m, 1H), 1.96 - 1.91 (m, 2H). LCMS (ESI): *m/z* = 501 $[M + H]^+$. Melting point: 222-224° C.

(S)-2-(1-(2,6-difluorobenzoyl)pyrrolidin-2-yl)-5-hydroxy-6-oxo-N-phenethyl-1,6-dihydropyrimidine-4-carboxamide (7b): ^{1}H NMR (400 MHz, Chloroform-*d*): δ ppm = 12.27 (s, 1H), 11.86 (s, 1H), 7.63 (t, *J* = 6.3 Hz, 1H), 7.37 (ddd, *J* = 14.0, 8.0, 6.1 Hz, 1H), 7.31 - 7.16 (m, 11H), 6.93 (dt, *J* = 18.7, 8.1 Hz, 2H), 5.13 (dd, *J* = 7.7, 4.3 Hz, 1H), 3.78 - 3.59 (m, 2H), 3.48 - 3.32 (m, 2H), 2.91 (t, *J* = 7.0 Hz, 2H), 2.35 – 2.25 (m, *J* = 5.9, 5.3 Hz, 2H), 2.0 -1.92 (m, *J* = 2H). LCMS (ESI): *m/z* = 469 $[M + H]^+$. Melting point: 241-243° C.

(S)-2-(1-(3,5-dichloroisonicotinoyl)pyrrolidin-2-yl)-5-hydroxy-6-oxo-N-phenethyl-1,6-dihydropyrimidine-4-carboxamide (7c): ^{1}H NMR (400 MHz, Chloroform-*d*): δ ppm = 12.37 (s, 1H), 8.57 (d, *J* = 5.3 Hz, 2H), 7.50 (t, *J* = 6.8 Hz, 1H), 7.36 – 7.28 (m, 2H), 7.28 – 7.20 (m, 3H), 5.20 (dd, *J* = 8.1, 2.9 Hz, 1H), 3.81 – 3.62 (m, 2H), 3.26 (t, *J* = 6.9 Hz, 2H), 2.98 – 2.88 (m, 2H), 2.55 – 2.42 (m, 1H), 2.23 – 2.10 (m, 1H), 2.05 – 1.93 (m, 2H). LCMS (ESI): *m/z* = 502 $[M + H]^+$. Melting point: 242-244° C.

(S)-2-(1-(2,5-dichloroisonicotinoyl)pyrrolidin-2-yl)-5-hydroxy-6-oxo-N-phenethyl-1,6-dihydropyrimidine-4-carboxamide (7d): ^{1}H NMR (400 MHz, DMSO-d_6): δ ppm = 12.82 (br s, 1H), 12.40 (br s, 1H), 8.67 – 8.07 (m, 2H), 7.69 (s, 1H), 7.38 – 7.08 (m, 4H), 4.84 – 4.29 (m, 1H), 3.57 – 3.37 (m, 4H), 2.94 – 2.61 (m, 2H), 2.34 – 2.13 (m, 1H), 2.10 – 1.75 (m, 3H). LCMS (ESI): *m/z* = 502 $[M + H]^+$.

(S)-2-(1-(2-chloroisonicotinoyl)pyrrolidin-2-yl)-5-hydroxy-6-oxo-N-phenethyl-1,6-dihydropyrimidine-4-carboxamide (7e): ^{1}H NMR (400 MHz, DMSO-d_6): δ ppm = 12.57 (br, 2H), 8.81 – 8.18 (m, 2H), 7.80 – 7.43 (m, 1H), 7.43 – 7.02 (m, 5H), 4.96 – 4.37 (m, 1H), 3.86 – 3.67 (m, 1H), 3.64 – 3.33 (m, 3H), 2.94 – 2.73 (m, 2H), 2.34 – 2.15 (m, 1H), 2.04 – 1.67 (m, 3H). LCMS (ESI): *m/z* = 468 $[M + H]^+$.

(S)-2-(1-(3,5-dichloroisonicotinoyl)pyrrolidin-2-yl)-5-hydroxy-6-oxo-N-(3-(trifluoromethyl)benzyl)-1,6-dihydropyrimidine-4-carboxamide (8a): ^{1}H NMR (400 MHz, Chloroform-*d*) δ ppm = 12.21 (s, 1H), 11.44 (br s, 1H), 8.55 (d, *J* = 14.7 Hz, 2H), 7.90 (t, *J* = 6.4 Hz, 1H), 7.63 – 7.47 (m, 4H), 5.21 (dd, *J* = 8.0, 3.8 Hz, 1H), 4.77 – 4.61 (m, 2H), 3.48 – 3.37 (m, 1H), 3.35 – 3.25 (m, 1H), 2.60 – 2.48 (m, 1H), 2.39 – 2.24 (m, 1H), 2.21 – 1.96 (m, 2H). LCMS (ESI): *m/z* = 556 $[M + H]^{+}$. Melting point: 236-238° C.

(S)-2-(1-(3,5-dichloroisonicotinoyl)pyrrolidin-2-yl)-5-hydroxy-1-methyl-6-oxo-N-phenethyl-1,6-dihydropyrimidine-4-carboxamide (8b): ^{1}H NMR (400 MHz, Chloroform-*d*) δ ppm = 12.09 (s, 1H), 8.57 (d, *J* = 11.4 Hz, 2H), 7.45 (br s, 1H), 7.26 – 7.25 (m, 1H), 7.24 – 7.15 (m, 3H), 7.12 – 7.06 (m, 1H), 5.32 (dd, *J* = 8.4, 2.6 Hz, 1H), 4.07 – 3.93 (m, 1H), 3.72 (s, 3H), 3.56 – 3.42 (m, 1H), 3.24 – 3.10 (m, 1H), 3.04 – 2.79 (m, 3H), 2.46 – 2.27 (m, 1H), 2.11 – 1.87 (m, 3H). LCMS (ESI): *m/z* = 516 $[M + H]^{+}$. Melting point: 105-108° C.

(S)-2-(1-(3,5-dichloroisonicotinoyl)pyrrolidin-2-yl)-5-hydroxy-6-oxo-N-(3-(trifluoromethyl)phenethyl)-1,6-dihydropyrimidine-4-carboxamide (8c): ^{1}H NMR (400 MHz, Chloroform-*d*) δ ppm = 12.28 (s, 1H), 8.60 – 8.55 (m, 2H), 7.54 – 7.48 (m, 2H), 7.48 – 7.43 (m, 2H), 5.19 (dd, *J* = 8.1, 3.2 Hz, 1H), 3.82 – 3.65 (m, 2H), 3.33 – 3.24 (m, 2H), 3.05 – 2.97 (m, 2H), 2.53 – 2.41 (m, 1H), 2.28 – 2.15 (m, 1H), 2.00 (p, *J* = 6.9 Hz, 2H). LCMS (ESI): *m/z* = 570 $[M + H]^{+}$. Melting point: 235-238° C.

(S)-2-(1-(3,5-dichloroisonicotinoyl)pyrrolidin-2-yl)-5-hydroxy-6-oxo-N-(2-phenoxyethyl)-1,6-dihydropyrimidine-4-carboxamide (8d): ^{1}H NMR (400 MHz, Chloroform-*d*) δ ppm = 12.27 (s, 1H), 11.26 (br s, 1H), 8.57 (s, 1H), 8.49 (s, 1H), 7.99 (t, *J* = 6.1 Hz, 1H), 7.32 – 7.27 (m, 2H), 7.01 – 6.94 (m, 1H), 6.92 – 6.87 (m, 2H), 5.23 (dd, *J* = 7.9, 3.1 Hz, 1H), 4.16 (t, *J* = 5.1 Hz, 2H), 3.94 – 3.75 (m, 2H), 3.41 – 3.23 (m, 2H), 2.69 – 2.57 (m, 1H), 2.35 – 2.12 (m, 2H), 2.12 – 1.97 (m, 1H). LCMS (ESI): *m/z* = 518 $[M + H]^{+}$. Melting point: 234-236° C.

(S)-2-(1-(3,5-dichloroisonicotinoyl)pyrrolidin-2-yl)-5-hydroxy-6-oxo-N-(2-(phenylsulfonyl)ethyl)-1,6-dihydropyrimidine-4-carboxamide (8e): ^{1}H NMR (400 MHz, Chloroform-*d*) δ ppm = 11.97 (s, 1H), 8.58 (s, 2H), 7.99 – 7.87 (m, 2H), 7.77 – 7.66 (m, 1H), 7.61 (t, *J* = 7.6 Hz, 2H), 5.29 (dd, *J* = 7.7, 1.9 Hz, 1H), 3.89 (p, *J* = 5.2 Hz, 2H), 3.44 – 3.24 (m, 4H), 2.99 – 2.86 (m, 1H), 2.38 – 2.04 (m, 3H). LCMS (ESI): *m/z* = 566 $[M + H]^{+}$. Melting point: 150-153° C.

(S)-2-(1-(3,5-dichloroisonicotinoyl)pyrrolidin-2-yl)-N-(2,3-dihydro-1H-inden-2-yl)-5-hydroxy-6-oxo-1,6-dihydropyrimidine-4-carboxamide (8f): ^{1}H NMR (400 MHz, Chloroform-*d*) δ ppm = 12.40 (s, 1H), 11.11 (br s, 1H), 8.56 (d, *J* = 7.2 Hz, 2H), 7.67 (d, *J* = 8.1 Hz, 1H), 7.32 – 7.18 (m, 4H), 5.17 (dd, *J* = 8.0, 3.7 Hz, 1H), 4.93 – 4.78 (m, 1H), 3.43 (dd, *J* = 16.2, 7.4 Hz, 2H), 3.39 – 3.24 (m, 2H), 2.95 (dt, *J* = 16.4, 4.8 Hz, 2H), 2.55 – 2.39 (m, 1H), 2.34 – 2.19 (m, 1H), 2.13 – 1.94 (m, 2H). LCMS (ESI): *m/z* = 514 $[M + H]^{+}$. Melting point: 155-158° C.

(S)-2-(1-(3,5-dichloroisonicotinoyl)pyrrolidin-2-yl)-N-(2,3-dihydro-1H-inden-2-yl)-5-hydroxy-1-methyl-6-oxo-1,6-dihydropyrimidine-4-carboxamide (8g): ^{1}H NMR (400 MHz, Chloroform-*d*) δ ppm = 12.03 (s, 1H), 8.56 (s, 1H), 8.50 (s, 1H), 7.66 (d, *J* = 8.6 Hz, 1H), 7.26 – 7.19 (m, 2H), 7.18 – 7.08 (m, 2H), 5.34 (dd, *J* = 8.4, 2.9 Hz, 1H), 4.94 – 4.76 (m, 1H), 3.74 (s, 3H), 3.39 (dt, *J* = 15.1, 7.0 Hz, 2H), 3.29 – 3.18 (m, 1H), 3.08 – 3.00 (m, 1H), 2.93 (dd, *J* = 16.1, 4.3 Hz, 1H), 2.84 (dd, *J* = 16.1, 4.3 Hz, 1H), 2.42 – 2.30 (m, 1H), 2.14 – 1.91 (m, 3H). LCMS (ESI): *m/z* = 528 $[M + H]^+$. Melting point: 140-143° C.

(S)-2-(1-(3,5-dichloroisonicotinoyl)pyrrolidin-2-yl)-N-((2,3-dihydro-1H-inden-2-yl)methyl)-5-hydroxy-6-oxo-1,6-dihydropyrimidine-4-carboxamide (8h): ^{1}H NMR (400 MHz, Chloroform-*d*) δ ppm = 12.42 (s, 1H), 11.08 (br s, 1H), 8.58 (d, *J* = 5.1 Hz, 2H), 7.66 – 7.57 (m, 1H), 7.25 – 7.19 (m, 2H), 7.18 – 7.12 (m, 2H), 5.28 – 5.18 (m, 1H), 3.63 – 3.47 (m, 2H), 3.43 – 3.26 (m, 2H), 3.14 (dd, *J* = 15.1, 7.2 Hz, 2H), 2.89 – 2.69 (m, 3H), 2.59 (s, 1H), 2.36 – 2.22 (m, 1H), 2.19 – 1.99 (m, 2H). LCMS (ESI): *m/z* = 528 $[M + H]^+$. Melting point: 230-233° C.

(S)-2-(1-(3-(2-chlorophenyl)propanoyl)pyrrolidin-2-yl)-5-hydroxy-6-oxo-N-phenethyl-1,6-dihydropyrimidine-4-carboxamide (9a): ^{1}H NMR (400 MHz, Chloroform-*d*) δ ppm = 12.24 (s, 1H), 11.48 (br s, 1H), 7.50 – 7.42 (m, 1H), 7.37 – 7.30 (m, 3H), 7.29 – 7.22 (m, 3H), 7.21 – 7.11 (m, 2H), 4.99 (d, *J* = 6.8 Hz, 1H), 3.76 – 3.63 (m, 2H), 3.46 – 3.31 (m, 2H), 3.11 (t, *J* = 7.5 Hz, 2H), 2.92 (t, *J* = 6.8 Hz, 2H), 2.74 – 2.64 (m, 2H), 2.50 – 2.40 (m, 1H), 1.98 – 1.80 (m, 3H). LCMS (ESI): *m/z* = 495 $[M + H]^+$. Melting point: 88-90° C.

(S)-2-(1-(3-(2-chlorophenyl)propanoyl)pyrrolidin-2-yl)-5-hydroxy-6-oxo-N-(2-phenoxyethyl)-1,6-dihydropyrimidine-4-carboxamide (9b): ^{1}H NMR (400 MHz, Chloroform-*d*) δ ppm = 12.14 (s, 1H), 11.49 (br s, 1H), 7.95 (t, *J* = 6.0 Hz, 1H), 7.35 – 7.27 (m, 4H), 7.16 (pd, *J* = 7.4, 1.8 Hz, 2H), 7.03 – 6.94 (m, 1H), 6.90 (dd, *J* = 8.8, 1.0 Hz, 2H), 5.04 (d, *J* = 7.3 Hz, 1H), 4.18 – 4.10 (m, 2H), 3.91 – 3.75 (m, 2H), 3.52 – 3.34 (m, 2H), 3.11 (t, *J* = 7.2 Hz, 2H), 2.71 – 2.62 (m, 3H), 2.13 – 1.89 (m, 3H). LCMS (ESI): *m/z* = 511 $[M + H]^+$. Melting point: 140-143° C.

(S)-2-(1-(3-(2-fluorophenyl)propanoyl)pyrrolidin-2-yl)-5-hydroxy-6-oxo-N-phenethyl-1,6-dihydropyrimidine-4-carboxamide (9c): ^{1}H NMR (400 MHz, Chloroform-*d*) δ ppm = 12.23 (s, 1H), 11.51 (s, 1H), 7.47 (t, *J* = 6.0 Hz, 1H), 7.36 – 7.30 (m, 2H), 7.28 – 7.21 (m, 4H), 7.21 – 7.14 (m, 1H), 7.08 – 6.95 (m, 2H), 4.98 (d, *J* = 6.1 Hz, 1H), 3.69 (qd, *J* = 6.6, 4.1 Hz, 2H), 3.50 – 3.42 (m, 1H), 3.41 – 3.33 (m, 1H), 3.01 (t, *J* = 7.7 Hz, 2H), 2.92 (t, *J* = 6.7 Hz, 2H), 2.64 (t, *J* = 7.6 Hz, 2H), 2.47 – 2.39 (m, 1H), 1.99 – 1.82 (m, 3H). LCMS (ESI): *m/z* = 479 $[M + H]^+$. Melting point: 140-143° C.

(S)-2-(1-(3-(2-fluorophenyl)propanoyl)pyrrolidin-2-yl)-5-hydroxy-6-oxo-N-(2-phenoxyethyl)-1,6-dihydropyrimidine-4-carboxamide (9d): ^{1}H NMR (400 MHz, Chloroform-*d*) δ ppm = 12.14 (br s, 1H), 11.47 (br s, 1H), 7.96 (t, *J* = 5.8 Hz, 1H), 7.33 –

7.27 (m, 2H), 7.25 – 7.14 (m, 2H), 7.08 – 7.02 (m, 1H), 7.02 – 6.95 (m, 2H), 6.94 – 6.83 (m, 2H), 5.04 (d, *J* = 7.5 Hz, 1H), 4.15 (t, *J* = 5.2 Hz, 2H), 3.89 – 3.78 (m, 2H), 3.52 – 3.33 (m, 2H), 3.02 (t, *J* = 7.6 Hz, 2H), 2.71 – 2.60 (m, 3H), 2.12 – 1.89 (m, 3H). LCMS (ESI): *m/z* = 495 $[M + H]^+$. Melting point: 154-157° C.

(S)-2-(1-(2-(2-chlorophenoxy)acetyl)pyrrolidin-2-yl)-5-hydroxy-6-oxo-N-phenethyl-1,6-dihydropyrimidine-4-carboxamide (9e): ^{1}H NMR (400 MHz, Chloroform-*d*) δ ppm = 12.17 (br s, 1H), 7.63 (t, *J* = 6.2 Hz, 1H), 7.36 – 7.28 (m, 3H), 7.27 – 7.18 (m, 3H), 7.14 (t, *J* = 7.0 Hz, 1H), 6.96 – 6.83 (m, 2H), 4.92 (dd, *J* = 8.6, 3.6 Hz, 1H), 4.87 (s, 2H), 4.05 – 3.94 (m, 1H), 3.75 – 3.59 (m, 3H), 2.90 (t, *J* = 7.1 Hz, 2H), 2.28 – 2.18 (m, 1H), 2.12 – 1.97 (m, 3H). LCMS (ESI): *m/z* = 497 $[M + H]^+$. Melting point: 116-118° C.

(S)-2-(1-(2-(2-chlorophenoxy)acetyl)pyrrolidin-2-yl)-5-hydroxy-6-oxo-N-(2-phenoxyethyl)-1,6-dihydropyrimidine-4-carboxamide (9f): ^{1}H NMR (400 MHz, Chloroform-*d*) δ ppm = 12.12 (s, 1H), 11.98 (br s, 1H), 7.99 (t, *J* = 5.7 Hz, 1H), 7.36 – 7.28 (m, 3H), 7.20 – 7.12 (m, 1H), 7.02 – 6.96 (m, 1H), 6.96 – 6.86 (m, 4H), 5.01 (dd, *J* = 7.8, 3.6 Hz, 1H), 4.92 – 4.82 (m, 2H), 4.15 (t, *J* = 5.1 Hz, 2H), 4.03 – 3.94 (m, 1H), 3.89 – 3.69 (m, 3H), 2.40 – 2.28 (m, 1H), 2.26 – 2.00 (m, 3H). LCMS (ESI): *m/z* = 513 $[M + H]^+$. Melting point: 166-168° C.

(S)-2-(1-(2-(2-fluorophenoxy)acetyl)pyrrolidin-2-yl)-5-hydroxy-6-oxo-N-phenethyl-1,6-dihydropyrimidine-4-carboxamide (9g): ^{1}H NMR (400 MHz, Chloroform-*d*) δ ppm = 12.21 (s, 1H), 12.00 (br s, 1H), 7.58 (t, *J* = 6.1 Hz, 1H), 7.36 – 7.30 (m, 2H), 7.29 – 7.21 (m, 4H), 7.10 – 6.88 (m, 3H), 4.97 (dd, *J* = 7.8, 3.8 Hz, 1H), 4.84 (s, 2H), 3.95 – 3.81 (m, 1H), 3.74 – 3.61 (m, 3H), 2.92 (t, *J* = 7.0 Hz, 2H), 2.27 – 2.09 (m, 2H), 2.07 – 1.95 (m, 2H). LCMS (ESI): *m/z* = 481 $[M + H]^+$. Melting point: 148-151° C.

(S)-2-(1-(2-(2-fluorophenoxy)acetyl)pyrrolidin-2-yl)-5-hydroxy-6-oxo-N-(2-phenoxyethyl)-1,6-dihydropyrimidine-4-carboxamide (9h): ^{1}H NMR (400 MHz, Chloroform-*d*) δ ppm = 12.11 (s, 1H), 11.95 (br s, 1H), 8.03 – 7.95 (m, 1H), 7.33 – 7.27 (m, 2H), 7.08 – 6.95 (m, 4H), 6.95 – 6.85 (m, 3H), 5.02 (dd, *J* = 7.8, 3.6 Hz, 1H), 4.84 (d, *J* = 2.0 Hz, 2H), 4.15 (t, *J* = 5.2 Hz, 2H), 3.96 – 3.90 (m, 1H), 3.88 – 3.63 (m, 3H), 2.41 – 2.30 (m, 1H), 2.26 – 2.00 (m, 3H). LCMS (ESI): *m/z* = 497 $[M + H]^+$. Melting point: 125-128° C.

(S)-2-(1-(2-((2-chlorophenyl)thio)acetyl)pyrrolidin-2-yl)-5-hydroxy-6-oxo-N-phenethyl-1,6-dihydropyrimidine-4-carboxamide (9i): ^{1}H NMR (400 MHz, Chloroform-*d*) δ ppm = 12.21 (s, 1H), 11.71 (br s, 1H), 7.57 – 7.50 (m, 2H), 7.37 – 7.30 (m, 3H), 7.28 – 7.15 (m, 5H), 4.91 (dd, *J* = 7.8, 2.9 Hz, 1H), 3.89 – 3.74 (m, 3H), 3.73 – 3.61 (m, 3H), 2.91 (t, *J* = 6.9 Hz, 2H), 2.35 – 2.21 (m, 1H), 2.14 – 1.93 (m, 3H). LCMS (ESI): *m/z* = 513 $[M + H]^+$. Melting point: 110-113° C.

2-((2S)-1-(2-((2-chlorophenyl)sulfinyl)acetyl)pyrrolidin-2-yl)-5-hydroxy-6-oxo-N-phenethyl-1,6-dihydropyrimidine-4-carboxamide (9j): ^{1}H NMR (400 MHz, Chloroform-*d*) δ ppm = 12.23 (s, 1H), 11.69 (br s, 1H), 7.95 – 7.83 (m, 1H), 7.74 (s, 1H), 7.59 – 7.36 (m, 3H), 7.35 – 7.28 (m, 2H), 7.28 – 7.17 (m, 3H), 4.96 (dd, *J* = 8.0, 2.9 Hz,

1H), 4.41 (d, *J* = 13.2 Hz, 1H), 4.06 – 3.92 (m, 1H), 3.90 – 3.79 (m, 1H), 3.74 – 3.56 (m, 3H), 2.93 (t, *J* = 7.1 Hz, 2H), 2.31 – 2.10 (m, 1H), 2.08 – 1.89 (m, 3H). LCMS (ESI): *m/z* = 529 $[M + H]^+$. Melting point: 158-162° C.

(S)-2-(1-(2-((2-chlorophenyl)thio)acetyl)pyrrolidin-2-yl)-5-hydroxy-6-oxo-N-(2-phenoxyethyl)-1,6-dihydropyrimidine-4-carboxamide (9k): ^{1}H NMR (400 MHz, Chloroform-*d*) δ ppm = 12.12 (s, 1H), 11.63 (br s, 1H), 7.97 (t, *J* = 5.6 Hz, 1H), 7.54 (dd, *J* = 7.4, 1.8 Hz, 1H), 7.38 – 7.28 (m, 3H), 7.25 – 7.14 (m, 2H), 6.99 (t, *J* = 7.4 Hz, 1H), 6.94 – 6.88 (m, 2H), 5.02 – 4.90 (m, 1H), 4.15 (t, *J* = 5.1 Hz, 2H), 3.93 – 3.76 (m, 5H), 3.76 – 3.62 (m, 1H), 2.54 – 2.42 (m, 1H), 2.23 – 2.01 (m, 3H). LCMS (ESI): *m/z* = 529 $[M + H]^+$. Melting point: 120-123° C.

(S)-2-(1-(2-((2-fluorophenyl)thio)acetyl)pyrrolidin-2-yl)-5-hydroxy-6-oxo-N-phenethyl-1,6-dihydropyrimidine-4-carboxamide (9l): ^{1}H NMR (400 MHz, Chloroform-*d*) δ ppm = 12.22 (s, 1H), 11.49 (br s, 1H), 7.57 – 7.51 (m, 1H), 7.47 (td, *J* = 7.5, 1.7 Hz, 1H), 7.34 (t, *J* = 7.1 Hz, 2H), 7.30 – 7.21 (m, 4H), 7.12 – 7.02 (m, 2H), 4.90 (d, *J* = 6.1 Hz, 1H), 3.87 – 3.56 (m, 6H), 2.92 (t, *J* = 6.9 Hz, 2H), 2.39 – 2.27 (m, 1H), 2.10 – 1.93 (m, 3H). LCMS (ESI): *m/z* = 497 $[M + H]^+$. Melting point: 98-101° C.

(S)-2-(1-(2-((2-fluorophenyl)thio)acetyl)pyrrolidin-2-yl)-5-hydroxy-6-oxo-N-(2-phenoxyethyl)-1,6-dihydropyrimidine-4-carboxamide (9m): ^{1}H NMR (400 MHz, Chloroform-*d*) δ ppm = 12.12 (s, 1H), 11.41 (s, 1H), 7.97 (t, *J* = 6.1 Hz, 1H), 7.48 (td, *J* = 7.4, 1.6 Hz, 1H), 7.34 – 7.26 (m, 3H), 7.11 – 7.02 (m, 2H), 7.02 – 6.96 (m, 1H), 6.95 – 6.87 (m, 2H), 4.98 – 4.92 (m, 1H), 4.15 (t, *J* = 5.2 Hz, 2H), 3.90 – 3.77 (m, 2H), 3.76 – 3.60 (m, 3H), 2.60 – 2.48 (m, 1H), 2.20 – 2.02 (m, 3H). LCMS (ESI): *m/z* = 513 $[M + H]^+$. Melting point: 140-142° C.

(S)-2-(1-(2-(2-chlorophenoxy)acetyl)pyrrolidin-2-yl)-5-hydroxy-6-oxo-N-(2-(phenylthio)ethyl)-1,6-dihydropyrimidine-4-carboxamide (10a): ^{1}H NMR (400 MHz, Chloroform-*d*) δ ppm = 12.36 (br s, 1H), 12.06 (s, 1H), 7.97 (t, *J* = 5.6 Hz, 1H), 7.44 – 7.36 (m, 2H), 7.34 – 7.27 (m, 3H), 7.25 – 7.19 (m, 1H), 7.19 – 7.10 (m, 1H), 6.97 – 6.84 (m, 2H), 4.99 (dd, *J* = 8.1, 4.1 Hz, 1H), 4.90 (d, *J* = 5.1 Hz, 2H), 4.12 – 4.00 (m, 1H), 3.83 – 3.69 (m, 1H), 3.60 (q, *J* = 6.4 Hz, 2H), 3.14 (t, *J* = 6.6 Hz, 2H), 2.34 – 2.02 (m, 4H). LCMS (ESI): *m/z* = 529 $[M + H]^+$. Melting point: 180-182° C.

(S)-2-(1-(2-((2-chlorophenyl)thio)acetyl)pyrrolidin-2-yl)-5-hydroxy-6-oxo-N-(2-(phenylthio)ethyl)-1,6-dihydropyrimidine-4-carboxamide (10b): ^{1}H NMR (400 MHz, Chloroform-*d*) δ ppm = 12.08 (s, 1H), 11.87 (br s, 1H), 7.96 (t, *J* = 6.2 Hz, 1H), 7.54 (d, *J* = 7.5 Hz, 1H), 7.43 – 7.37 (m, 2H), 7.38 – 7.27 (m, 3H), 7.25 – 7.13 (m, 3H), 4.96 (d, *J* = 6.7 Hz, 1H), 4.04 – 3.77 (m, 1H), 3.72 (q, *J* = 8.0 Hz, 1H), 3.60 (q, *J* = 6.4 Hz, 2H), 3.14 (t, *J* = 6.5 Hz, 2H), 2.48 – 2.32 (m, 1H), 2.25 – 2.05 (m, 3H). LCMS (ESI): *m/z* = 545 $[M + H]^+$. Melting point: 120-123° C.

2-((S)-1-(2-(2-chlorophenoxy)acetyl)pyrrolidin-2-yl)-5-hydroxy-6-oxo-N-(2-(phenylsulfinyl)ethyl)-1,6-dihydropyrimidine-4-carboxamide (10c): ^{1}H NMR (400 MHz, Chloroform-*d*) δ ppm = 11.97 (s, 1H), 8.54 (dt, *J* = 28.2, 5.8 Hz, 1H), 7.67 – 7.45

(m, 4H), 7.34 (dd, *J* = 7.9, 1.6 Hz, 1H), 7.19 – 7.10 (m, 1H), 6.99 – 6.85 (m, 2H), 5.08 – 4.99 (m, 1H), 4.96 – 4.81 (m, 2H), 4.04 – 3.96 (m, 1H), 3.95 – 3.84 (m, 1H), 3.82 – 3.65 (m, 2H), 3.31 – 3.17 (m, 1H), 2.97 – 2.87 (m, 1H), 2.47 – 2.35 (m, 1H), 2.29 – 2.00 (m, 3H). LCMS (ESI): *m/z* = 545 $[M + H]^+$. Melting point: 130-132° C.

(S)-2-(1-(2-(2-chlorophenoxy)acetyl)pyrrolidin-2-yl)-5-hydroxy-6-oxo-N-(2-(phenylsulfonyl)ethyl)-1,6-dihydropyrimidine-4-carboxamide (10d): ^{1}H NMR (400 MHz, Chloroform-*d*) δ ppm = 11.81 (s, 1H), 8.49 (t, *J* = 5.6 Hz, 1H), 7.91 (d, *J* = 7.7 Hz, 2H), 7.68 (t, *J* = 7.2 Hz, 1H), 7.58 (t, *J* = 7.6 Hz, 2H), 7.35 (d, *J* = 7.9 Hz, 1H), 7.22 – 7.12 (m, 1H), 6.98 – 6.88 (m, 2H), 5.12 – 5.01 (m, 1H), 4.88 (s, 2H), 4.08 – 3.80 (m, 3H), 3.78 – 3.67 (m, 1H), 3.47 – 3.28 (m, 2H), 2.50 – 2.32 (m, 1H), 2.30 – 2.07 (m, 3H). LCMS (ESI): *m/z* = 561 $[M + H]^+$. Melting point: 137-139° C.

(S)-2-(1-(2-((2-chlorophenyl)thio)acetyl)pyrrolidin-2-yl)-5-hydroxy-6-oxo-N-(2-(phenylsulfonyl)ethyl)-1,6-dihydropyrimidine-4-carboxamide (10e): ^{1}H NMR (400 MHz, Chloroform-*d*) δ ppm = 11.83 (br s, 1H), 11.50 (br s, 1H), 8.51 (t, *J* = 6.1 Hz, 1H), 7.92 (dt, *J* = 7.1, 1.5 Hz, 2H), 7.69 (t, *J* = 7.5 Hz, 1H), 7.59 (t, *J* = 7.5 Hz, 1H), 7.53 (dd, *J* = 7.5, 1.9 Hz, 1H), 7.37 (dd, *J* = 7.1, 2.3 Hz, 1H), 7.25 – 7.15 (m, 2H), 5.00 (dd, *J* = 8.0, 2.6 Hz, 1H), 3.94 – 3.77 (m, 5H), 3.73 – 3.62 (m, 1H), 3.36 (t, *J* = 5.7 Hz, 2H), 2.67 – 2.57 (m, 1H), 2.34 – 2.21 (m, 1H), 2.19 – 2.06 (m, 2H). LCMS (ESI): *m/z* = 577 $[M + H]^+$.

(S)-2-(1-(2-(2-chlorophenoxy)acetyl)pyrrolidin-2-yl)-5-hydroxy-N-(2-((4-methoxyphenyl)sulfonyl)ethyl)-6-oxo-1,6-dihydropyrimidine-4-carboxamide (10f): ^{1}H NMR (400 MHz, Chloroform-*d*) δ ppm = 11.86 (s, 1H), 8.53 (t, *J* = 5.9 Hz, 1H), 7.82 (d, *J* = 8.9 Hz, 2H), 7.36 (dd, *J* = 7.8, 1.6 Hz, 1H), 7.20 – 7.14 (m, 1H), 7.02 (d, *J* = 9.0 Hz, 2H), 6.97 – 6.89 (m, 2H), 5.07 (dd, *J* = 7.8, 2.4 Hz, 1H), 4.85 (d, *J* = 4.0 Hz, 2H), 3.96 – 3.80 (m, 3H), 3.87 (s, 3H), 3.79 – 3.65 (m, 1H), 3.32 (t, *J* = 5.8 Hz, 2H), 2.58 – 2.46 (m, 1H), 2.33 – 2.06 (m, 3H). LCMS (ESI): *m/z* = 591 $[M + H]^+$. Melting point: 152-154° C.

(S)-2-(1-(2-((2-chlorophenyl)thio)acetyl)pyrrolidin-2-yl)-5-hydroxy-N-(2-((4-methoxyphenyl)sulfonyl)ethyl)-6-oxo-1,6-dihydropyrimidine-4-carboxamide (10g): ^{1}H NMR (400 MHz, Chloroform-*d*) δ ppm = 11.87 (s, 1H), 11.35 (s, 1H), 8.53 (t, *J* = 5.8 Hz, 1H), 7.83 (d, *J* = 8.8 Hz, 2H), 7.56 – 7.50 (m, 1H), 7.40 – 7.34 (m, 1H), 7.24 – 7.17 (m, 2H), 7.03 (d, *J* = 8.9 Hz, 2H), 5.00 (d, *J* = 7.9 Hz, 1H), 3.88 (s, 3H), 3.87 – 3.81 (m, 2H), 3.81 – 3.77 (m, 2H), 3.67 (q, *J* = 9.1 Hz, 1H), 3.32 (t, *J* = 5.6 Hz, 2H), 2.75 – 2.60 (m, 1H), 2.36 – 2.22 (m, 1H), 2.20 – 2.03 (m, 2H). LCMS (ESI): *m/z* = 607 $[M + H]^+$. Melting point: 143-145° C.

(S)-2-(1-(2-(2-chlorophenoxy)acetyl)pyrrolidin-2-yl)-N-(2,3-dihydro-1H-inden-2-yl)-5-hydroxy-6-oxo-1,6-dihydropyrimidine-4-carboxamide (10h): ^{1}H NMR (400 MHz, Chloroform-*d*) δ ppm = 12.27 (s, 2H), 7.78 (d, *J* = 8.8 Hz, 1H), 7.32 (dd, *J* = 7.8, 1.5 Hz, 1H), 7.30 – 7.19 (m, 4H), 7.19 – 7.13 (m, 1H), 6.92 (dtd, *J* = 14.3, 7.7, 1.3 Hz, 2H), 4.96 (dd, *J* = 7.7, 4.6 Hz, 1H), 4.91 – 4.75 (m, 3H), 3.99 (dt, *J* = 11.6, 6.1 Hz, 1H), 3.81 – 3.67 (m, 1H), 3.41 (dd, *J* = 16.1, 7.4 Hz, 2H), 2.95 (dt, *J* = 15.9, 6.5 Hz, 2H), 2.29 – 2.13

(m, 2H), 2.10 – 1.97 (m, 2H). LCMS (ESI): *m/z* = 509 $[M + H]^+$. Melting point: 208-210° C.

(S)-2-(1-(2-((2-chlorophenyl)thio)acetyl)pyrrolidin-2-yl)-N-(2,3-dihydro-1H-inden-2-yl)-5-hydroxy-6-oxo-1,6-dihydropyrimidine-4-carboxamide (10i): ^{1}H NMR (400 MHz, Chloroform-*d*) δ ppm = 12.27 (br s, 1H), 12.21 (s, 1H), 7.74 (d, *J* = 8.0 Hz, 1H), 7.56 – 7.47 (m, 1H), 7.32 (d, *J* = 7.7 Hz, 1H), 7.28 – 7.09 (m, 4H), 4.92 – 4.75 (m, 2H), 4.17 – 3.80 (m, 3H), 3.80 – 3.62 (m, 1H), 3.46 – 3.34 (m, 2H), 3.02 – 2.86 (m, 2H), 2.30 – 2.09 (m, 2H), 2.04 (d, *J* = 7.4 Hz, 2H). LCMS (ESI): *m/z* = 525 $[M + H]^+$. Melting point: 121-123° C.

(S)-2-(1-(2-((2-chlorophenyl)thio)acetyl)pyrrolidin-2-yl)-N-(5,6-dimethoxy-2,3-dihydro-1H-inden-2-yl)-5-hydroxy-6-oxo-1,6-dihydropyrimidine-4-carboxamide (10j): ^{1}H NMR (400 MHz, Chloroform-*d*) δ ppm = 12.25 (s, 1H), 12.02 (br s, 1H), 7.73 (d, *J* = 8.2 Hz, 1H), 7.51 (d, *J* = 7.3 Hz, 1H), 7.34 (dd, *J* = 7.6, 1.5 Hz, 1H), 7.24 – 7.10 (m, 2H), 6.78 (d, *J* = 8.9 Hz, 2H), 4.96 – 4.77 (m, 2H), 4.03 – 3.95 (m, 1H), 3.87 (d, *J* = 3.3 Hz, 6H), 3.86 – 3.78 (m, 2H), 3.77 – 3.65 (m, 1H), 3.35 (dd, *J* = 15.8, 7.3 Hz, 2H), 2.94 – 2.78 (m, 2H), 2.27 – 2.17 (m, 2H), 2.11 – 1.98 (m, 2H). LCMS (ESI): *m/z* = 585 $[M + H]^+$. Melting point: 139-141° C.

2-((S)-1-(2-((2-chlorophenyl)thio)acetyl)pyrrolidin-2-yl)-N-((2,3-dihydrobenzofuran-2-yl)methyl)-5-hydroxy-6-oxo-1,6-dihydropyrimidine-4-carboxamide (10k): ^{1}H NMR (400 MHz, Chloroform-*d*) δ ppm = 12.05 (s, 1H), 11.69 (s, 1H), 7.94 – 7.84 (m, 1H), 7.52 (t, *J* = 7.4 Hz, 1H), 7.39 – 7.33 (m, 1H), 7.24 – 7.07 (m, 4H), 6.86 (tdd, *J* = 7.4, 3.6, 1.0 Hz, 1H), 6.79 (dd, *J* = 8.0, 2.6 Hz, 1H), 5.06 – 4.96 (m, 1H), 4.95 – 4.87 (m, 1H), 3.96 – 3.74 (m, 3H), 3.75 – 3.57 (m, 3H), 3.36 (dd, *J* = 15.7, 9.4 Hz, 1H), 2.98 (ddd, *J* = 15.8, 7.2, 3.3 Hz, 1H), 2.43 – 2.23 (m, 1H), 2.09 (s, 2H), 2.02 – 1.93 (m, 1H). LCMS (ESI): *m/z* = 541 $[M + H]^+$. Melting point: 115-118° C.

(S)-N-(benzofuran-2-ylmethyl)-2-(1-(2-((2-chlorophenyl)thio)acetyl)pyrrolidin-2-yl)-5-hydroxy-6-oxo-1,6-dihydropyrimidine-4-carboxamide (10l): ^{1}H NMR (400 MHz, Chloroform-*d*) δ ppm = 11.98 (s, 1H), 7.96 (t, *J* = 6.2 Hz, 1H), 7.56 – 7.49 (m, 2H), 7.48 – 7.42 (m, 1H), 7.35 – 7.27 (m, 2H), 7.25 – 7.12 (m, 3H), 6.68 (d, *J* = 0.9 Hz, 1H), 4.93 (dd, *J* = 8.0, 3.5 Hz, 1H), 4.73 (d, *J* = 6.2 Hz, 2H), 4.07 – 3.91 (m, 1H), 3.91 – 3.79 (m, 2H), 3.71 (q, *J* = 8.0 Hz, 1H), 2.39 – 2.28 (m, 1H), 2.29 – 1.98 (m, 3H). LCMS (ESI): *m/z* = 539 $[M + H]^+$. Melting point: 132-134° C.

(S)-2-(1-(2-(2-fluorophenoxy)acetyl)pyrrolidin-2-yl)-5-hydroxy-6-oxo-N-(2-(pyridin-2-yl)ethyl)-1,6-dihydropyrimidine-4-carboxamide (10m): ^{1}H NMR (400 MHz, Chloroform-*d*) δ ppm = 12.13 (br s, 1H), 8.59 (d, *J* = 5.0 Hz, 1H), 8.50 – 8.38 (m, 1H), 8.04 (s, 1H), 7.99 – 7.84 (m, 1H), 7.50 – 7.35 (m, 2H), 7.11 – 6.96 (m, 3H), 6.96 – 6.87 (m, 1H), 5.05 – 4.98 (m, 1H), 4.96 – 4.76 (m, 2H), 3.99 – 3.83 (m, 3H), 3.66 (q, *J* = 8.3 Hz, 1H), 3.43 – 3.26 (m, 2H), 2.56 – 2.38 (m, 1H), 2.25 – 2.01 (m, 3H). LCMS (ESI): *m/z* = 482 $[M + H]^+$. Melting point: 138-140° C.

(S)-2-(1-(2-(2-fluorophenoxy)acetyl)pyrrolidin-2-yl)-5-hydroxy-6-oxo-N-(2-(pyridin-4-yl)ethyl)-1,6-dihydropyrimidine-4-carboxamide (10n): ^{1}H NMR (400 MHz, DMSO-d_6) δ ppm = 12.62 (s, 1H), 12.36 (s, 1H), 8.65 (t, *J* = 6.2 Hz, 1H), 8.47 (d, *J* = 4.6 Hz, 2H), 7.27 (d, *J* = 4.8 Hz, 2H), 7.18 (dd, *J* = 11.8, 7.9 Hz, 1H), 7.10 – 6.95 (m, 2H), 6.94 – 6.83 (m, 1H), 5.02 – 4.82 (m, 2H), 4.68 (dd, *J* = 8.2, 4.0 Hz, 1H), 3.89 – 3.76 (m, 1H), 3.71 – 3.47 (m, 3H), 2.89 (t, *J* = 7.3 Hz, 2H), 2.31 – 2.13 (m, 1H), 2.06 – 1.83 (m, 3H). LCMS (ESI): *m/z* = 482 $[M + H]^+$. Melting point: 203-205° C.

Proton NMR Spectra

The solvent is $CDCl_3$ unless otherwise indicated in the upper left-hand corner of the spectrum.

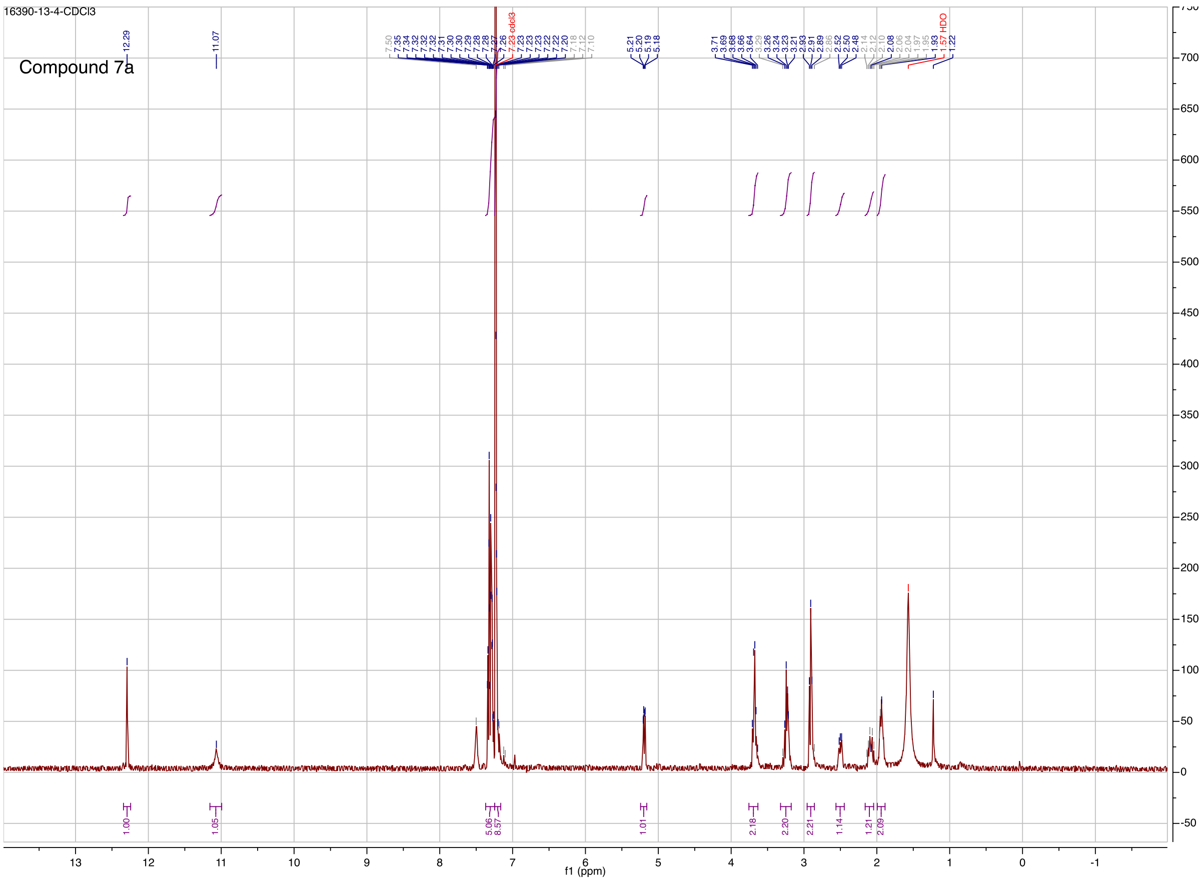

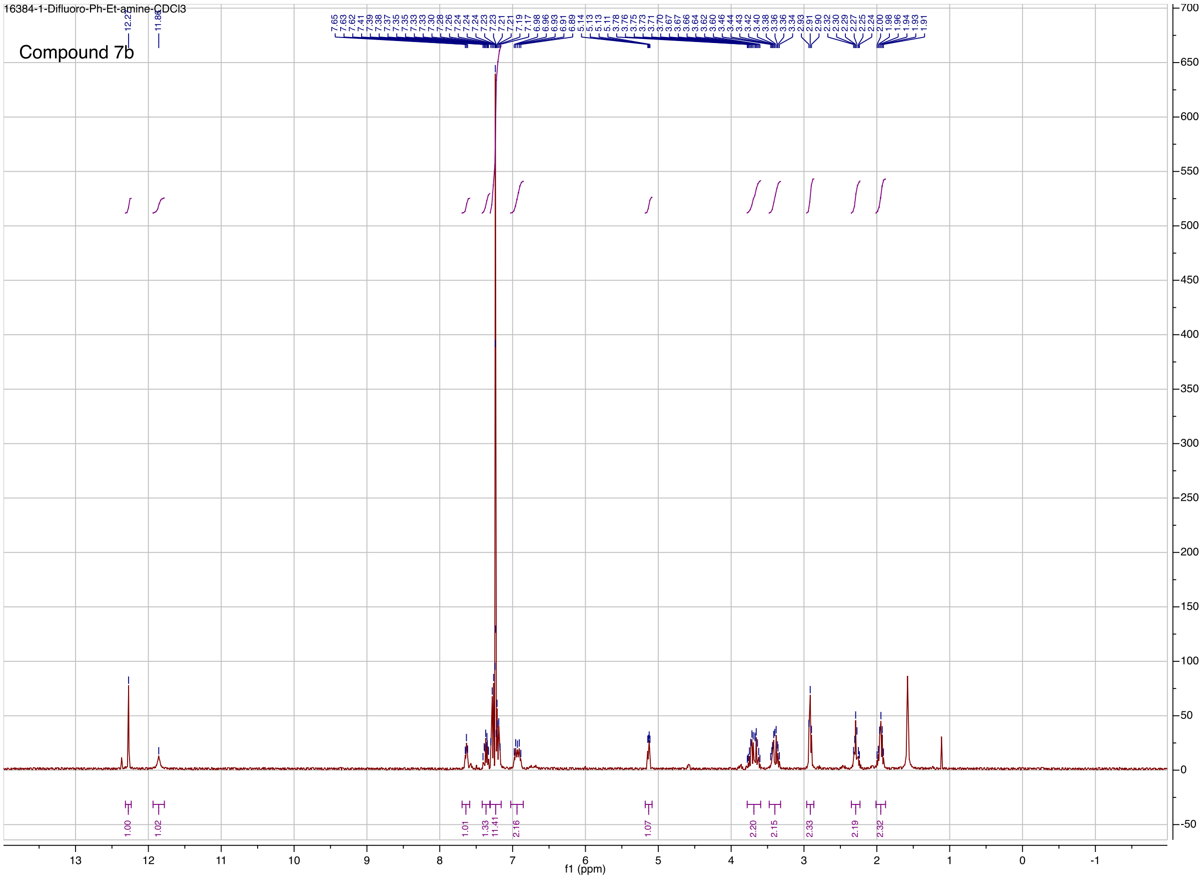
16384-1-Difluoro-Ph-Et-amine-CDCl3
Compound 7b
f1 (ppm)

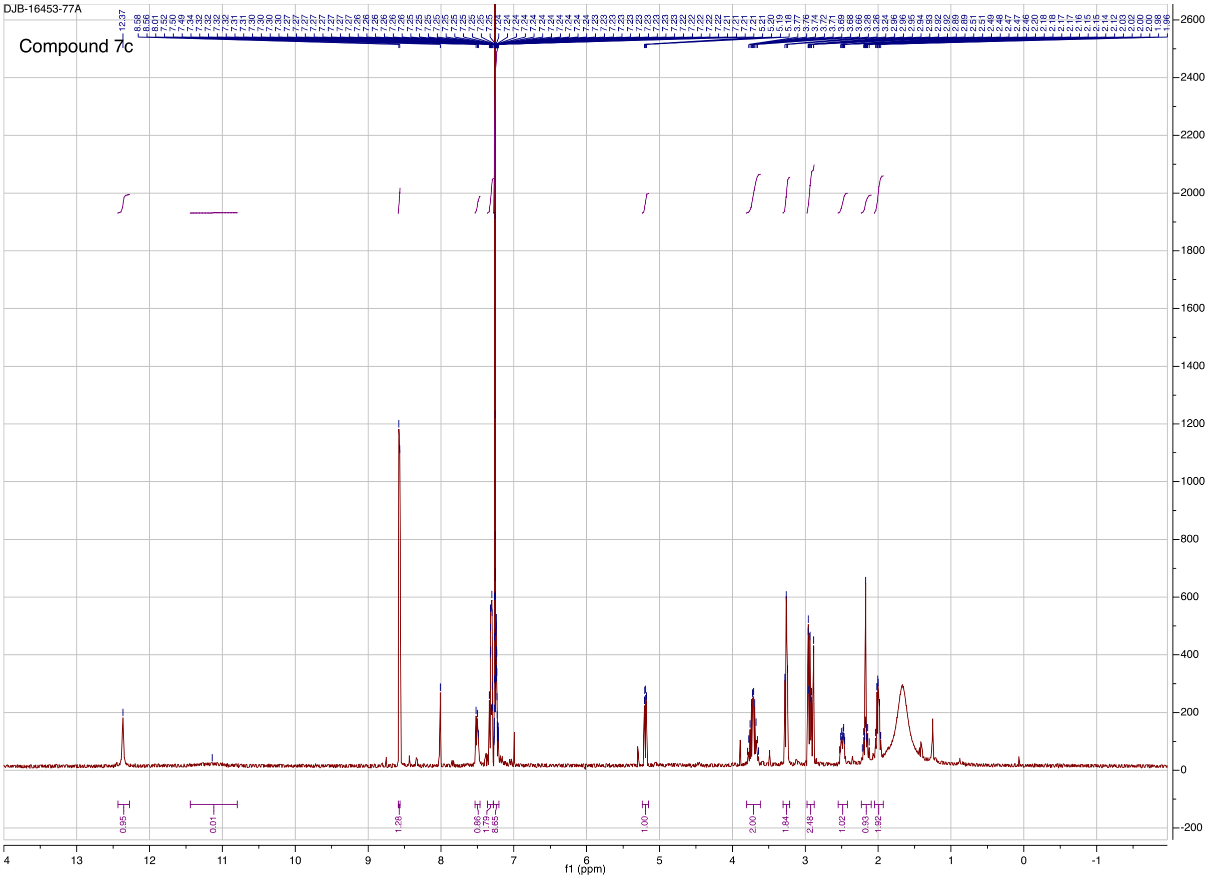

DJB-16453-77A
Compound 7c
f1 (ppm)

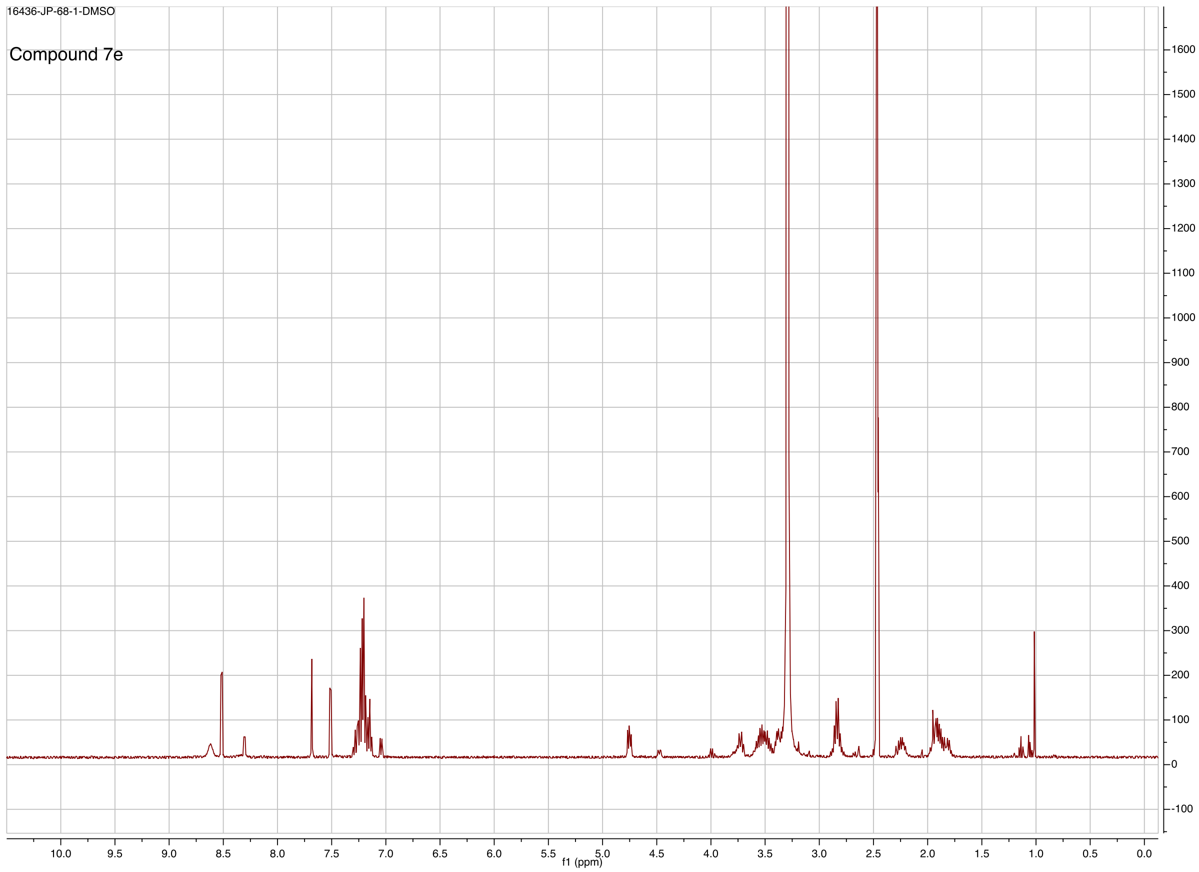
16436-JP-68-1-DMSO
Compound 7e
f1 (ppm)

DJB-16453-78Cvac

Compound 8a

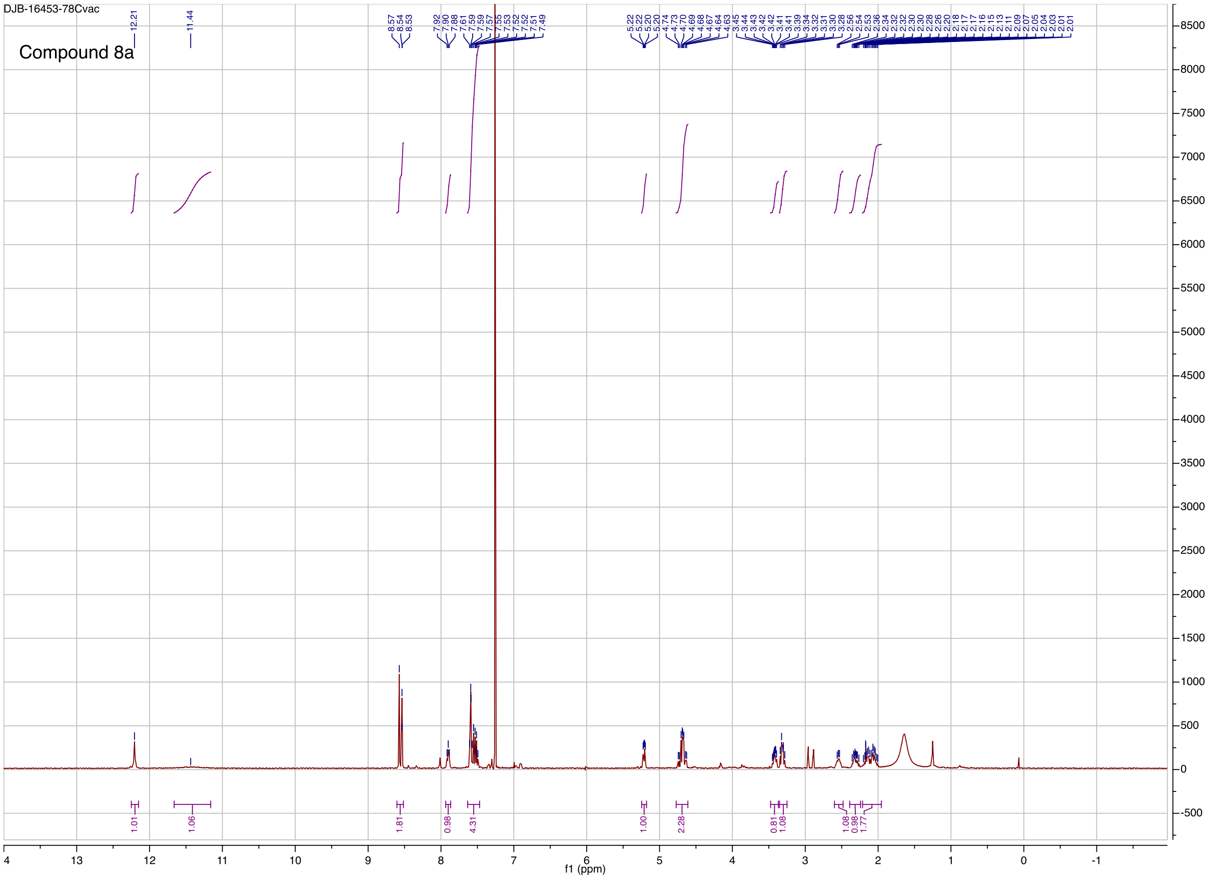

DJB-16453-77A

Compound 8b

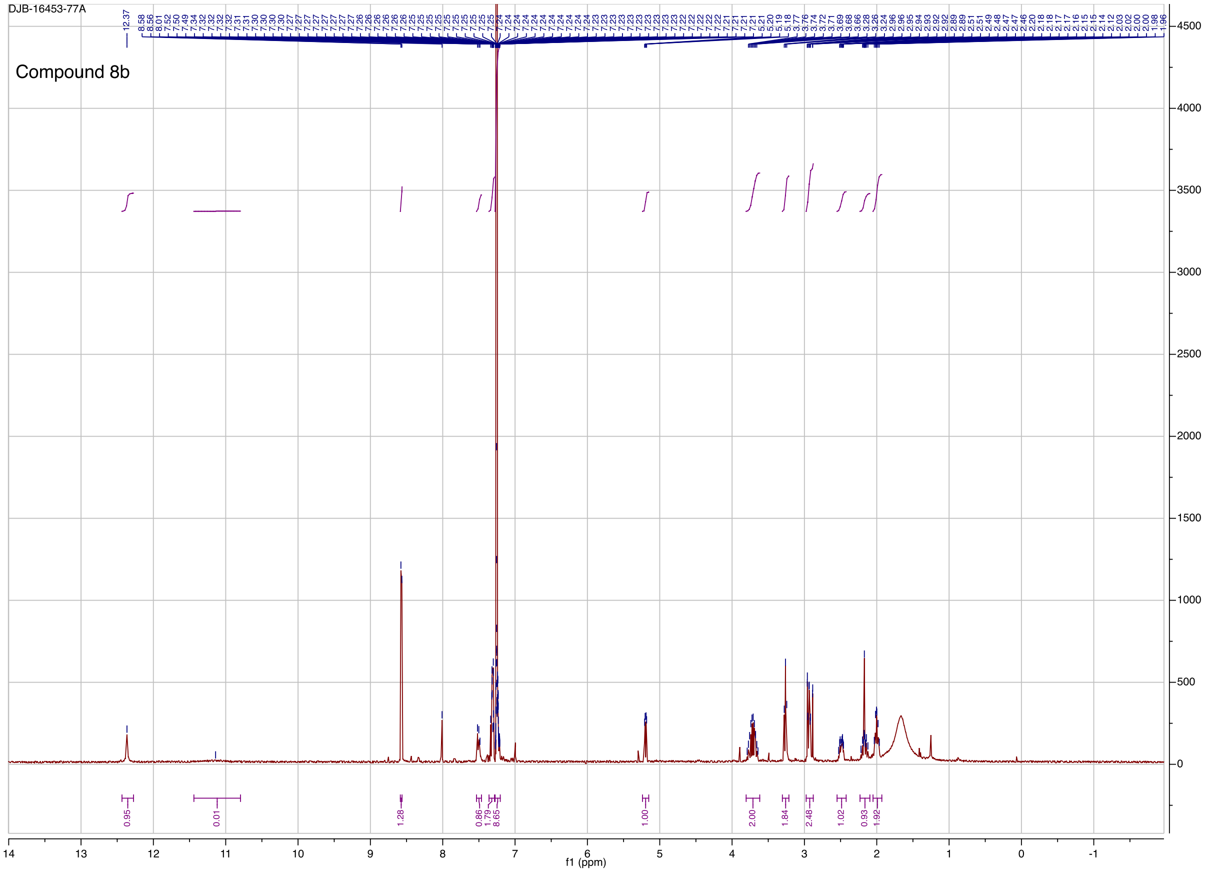

DJB-16453-77C

Compound 8C

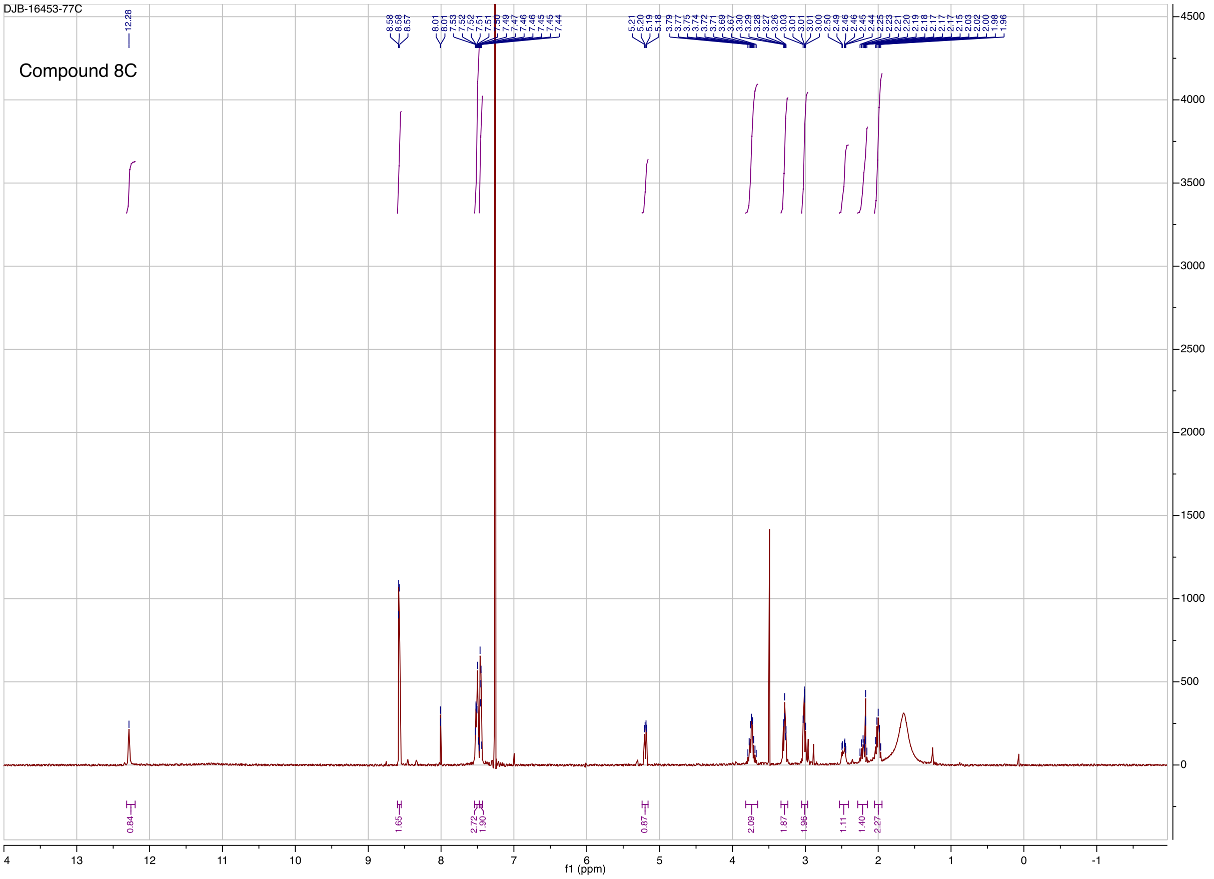

DJB-16453-77B

Compound 8D

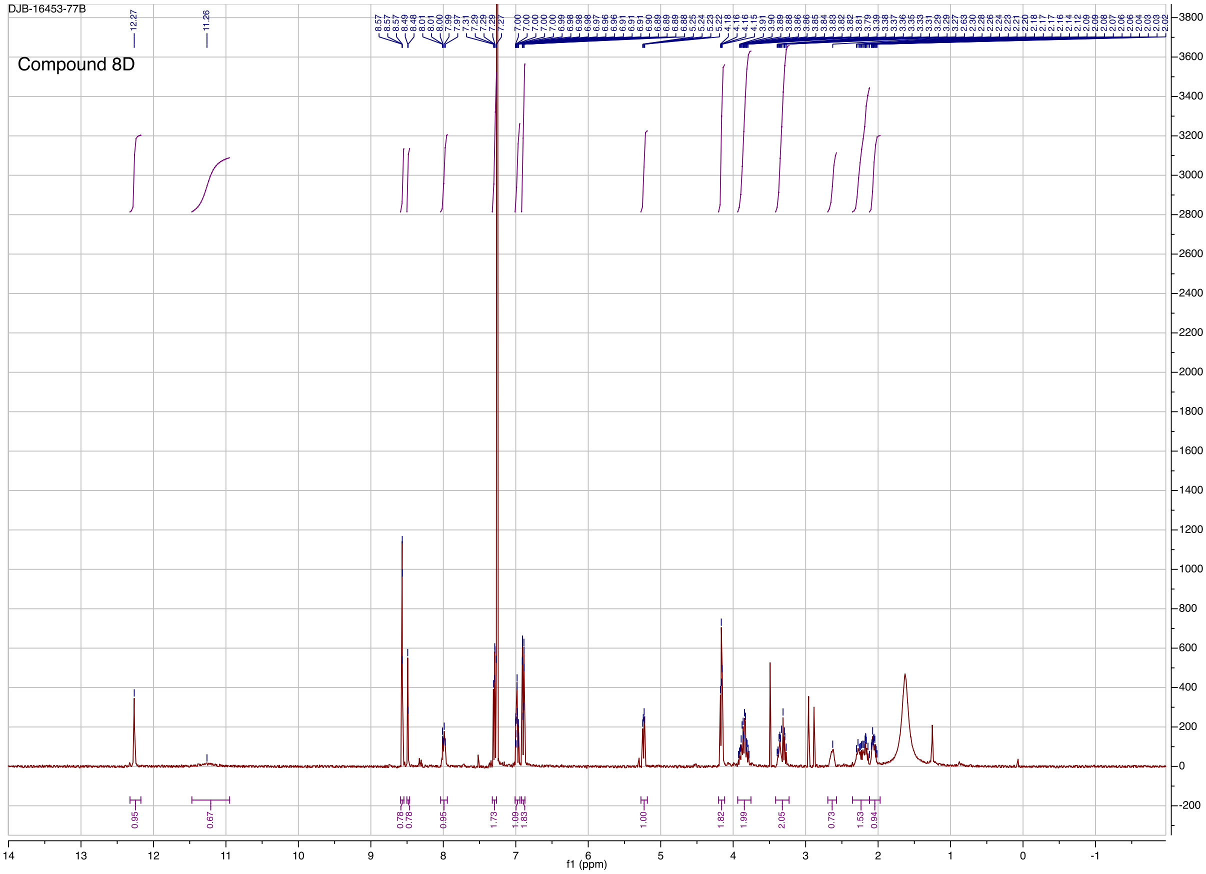

DJB-16494-61A

Compound 8e

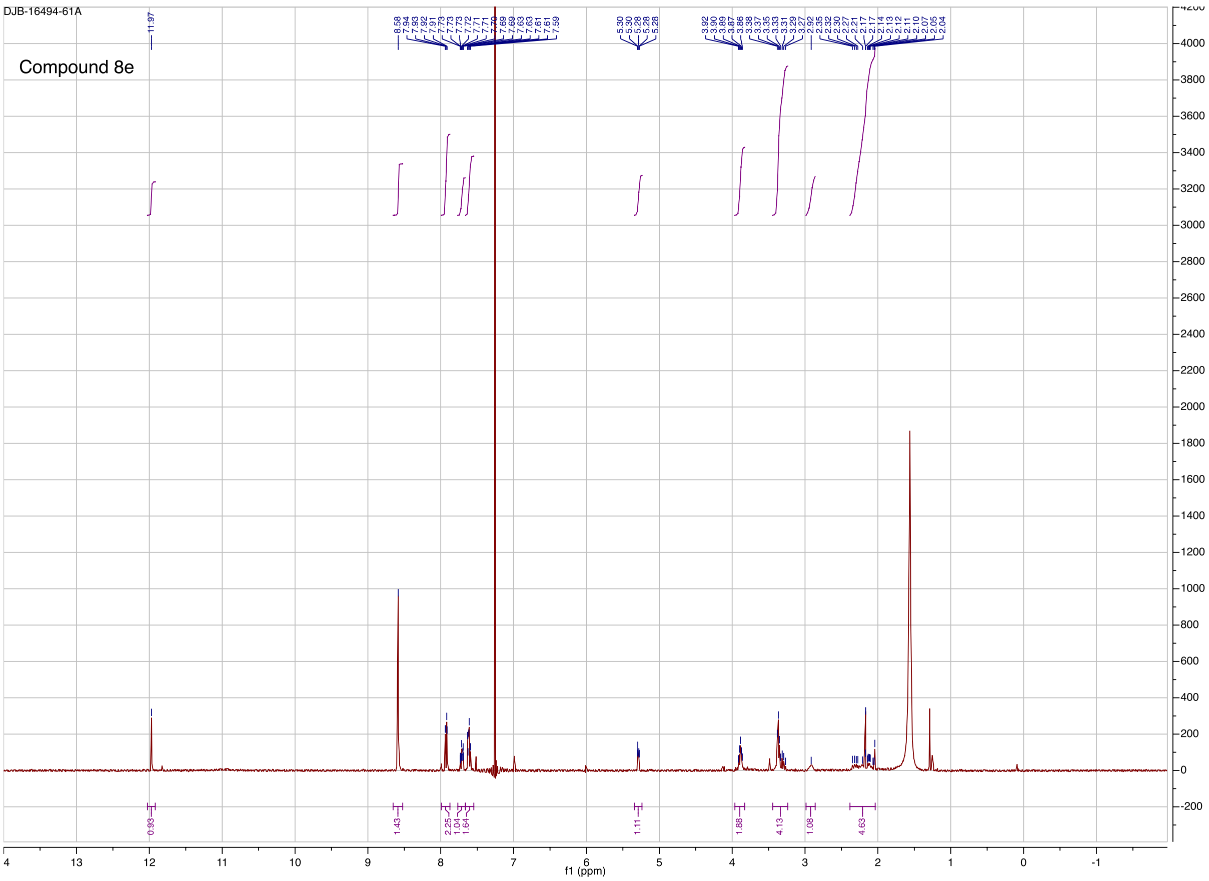

DJB-16453-78A

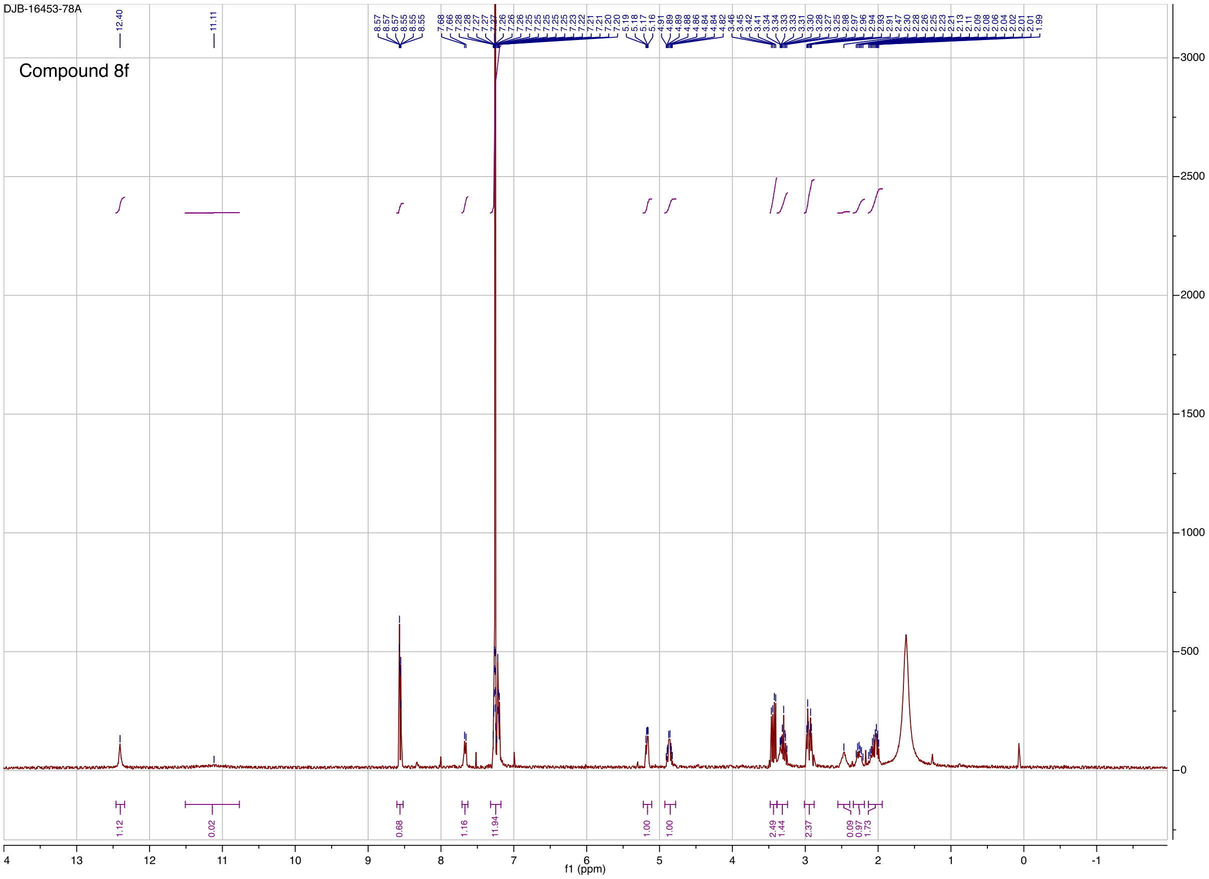

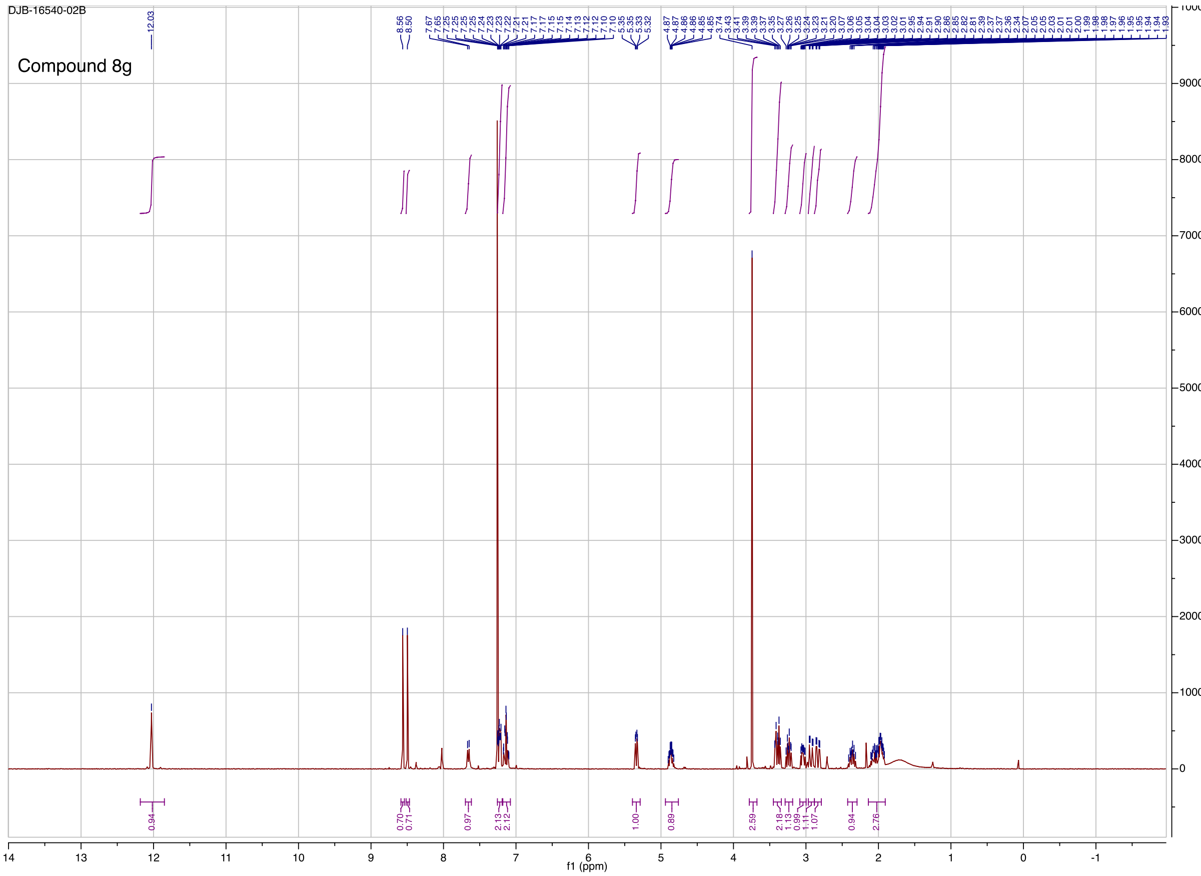

DJB-16540-02B
Compound 8g
f1 (ppm)

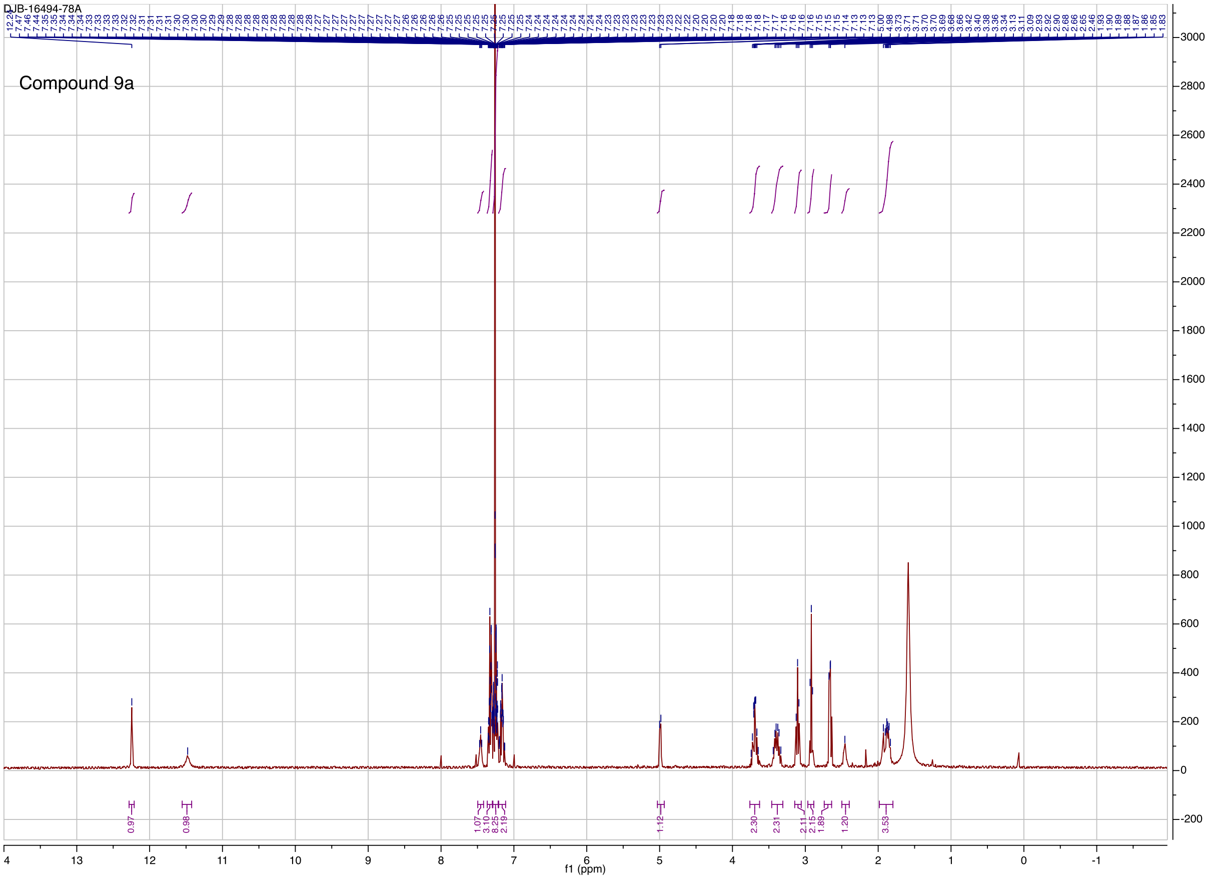

DJB-16494-78A
Compound 9a
f1 (ppm)

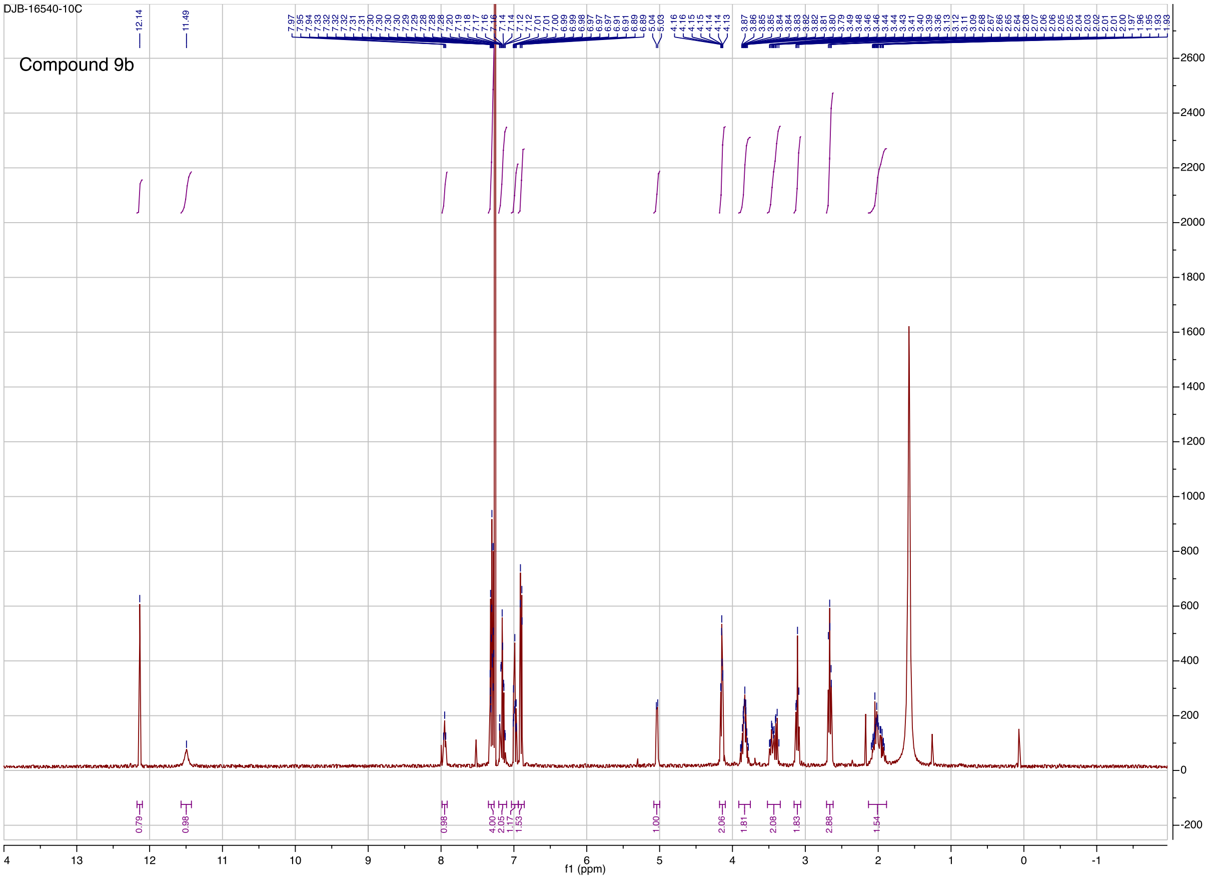
DJB-16540-10C
Compound 9b
f1 (ppm)

DJB-16453-45A

Compound 9c

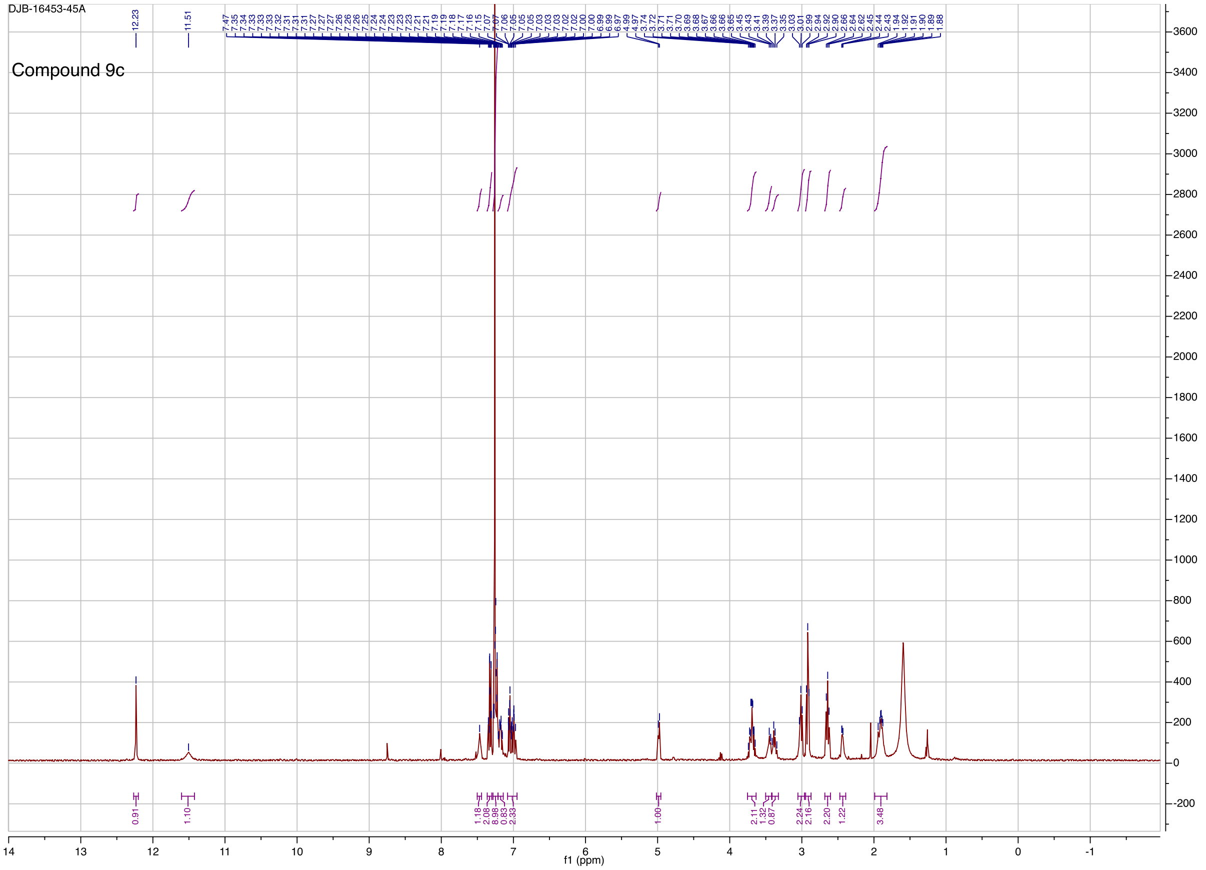

DJB-16453-45B

Compound 9d

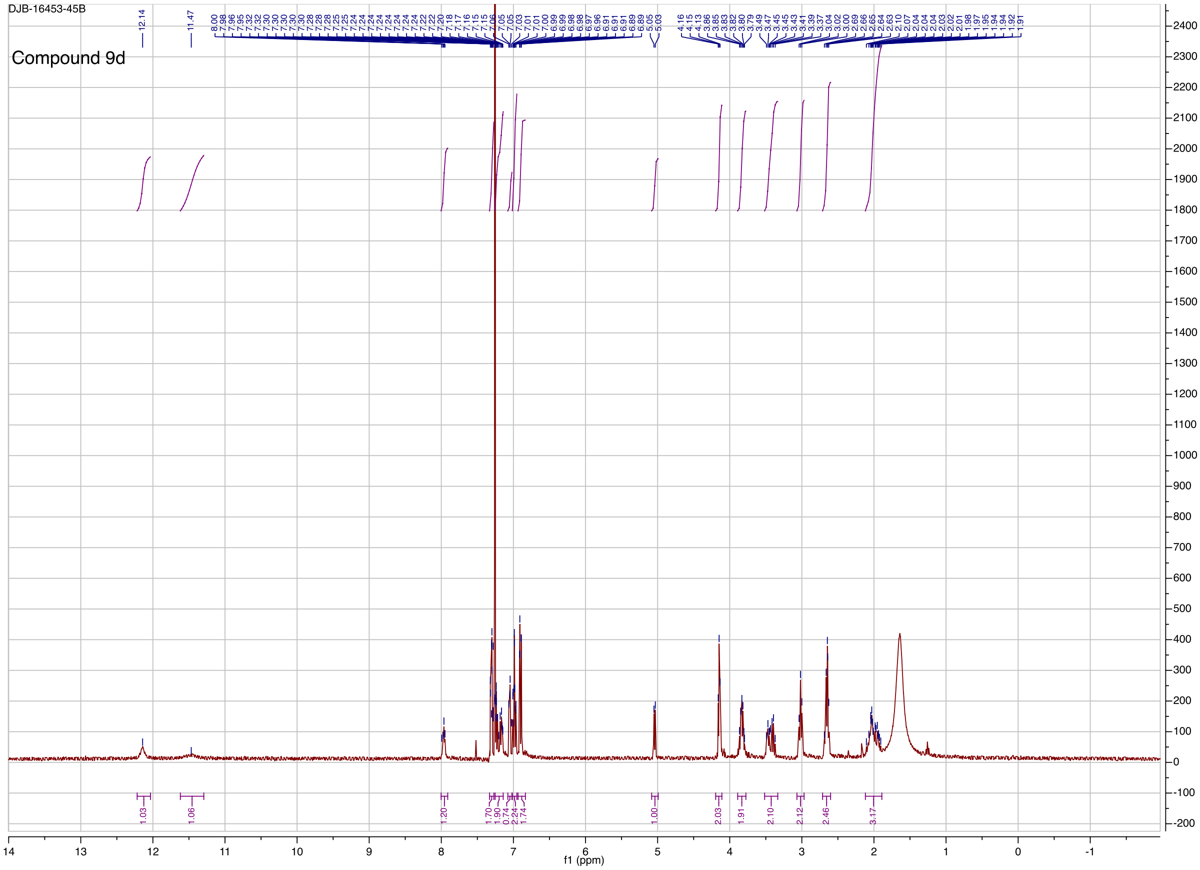

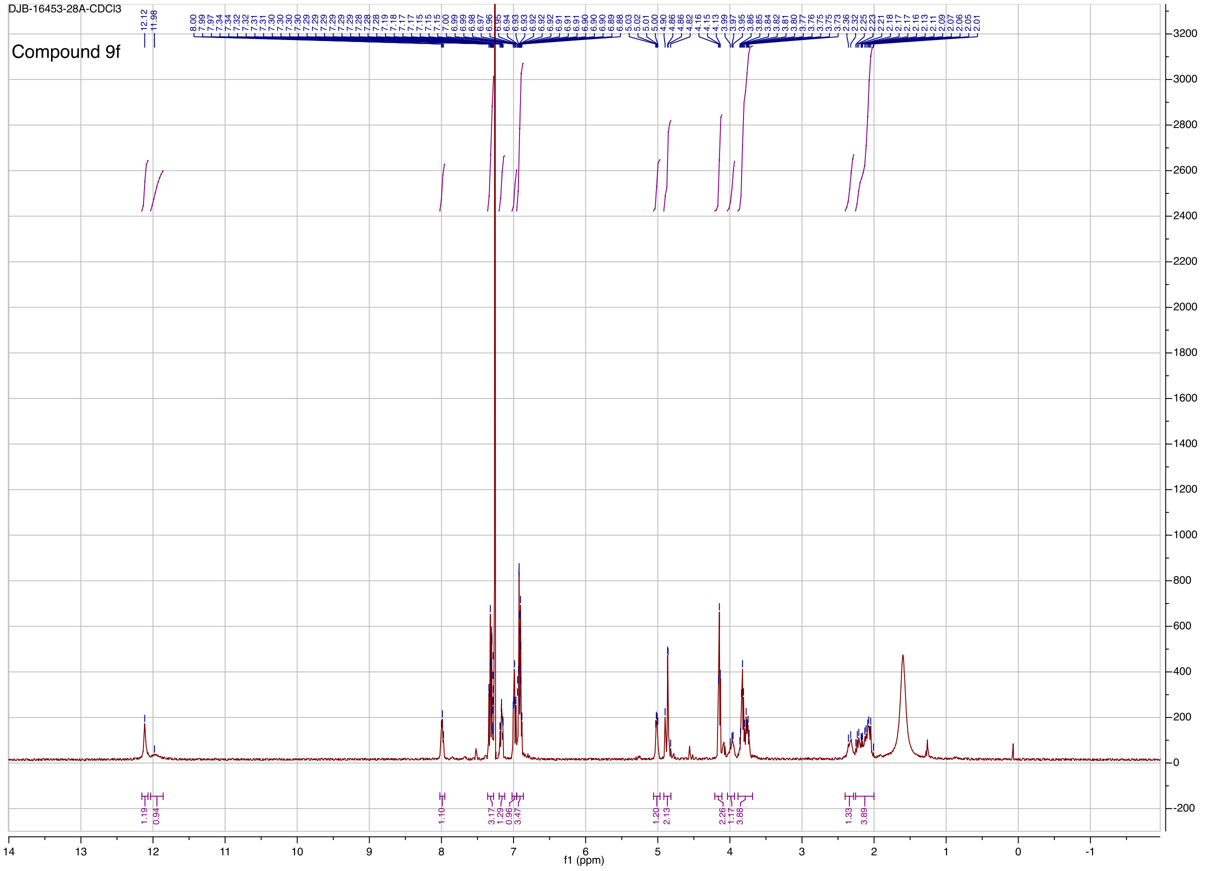

DJB-16453-28A-CDCl3
Compound 9f
f1 (ppm)

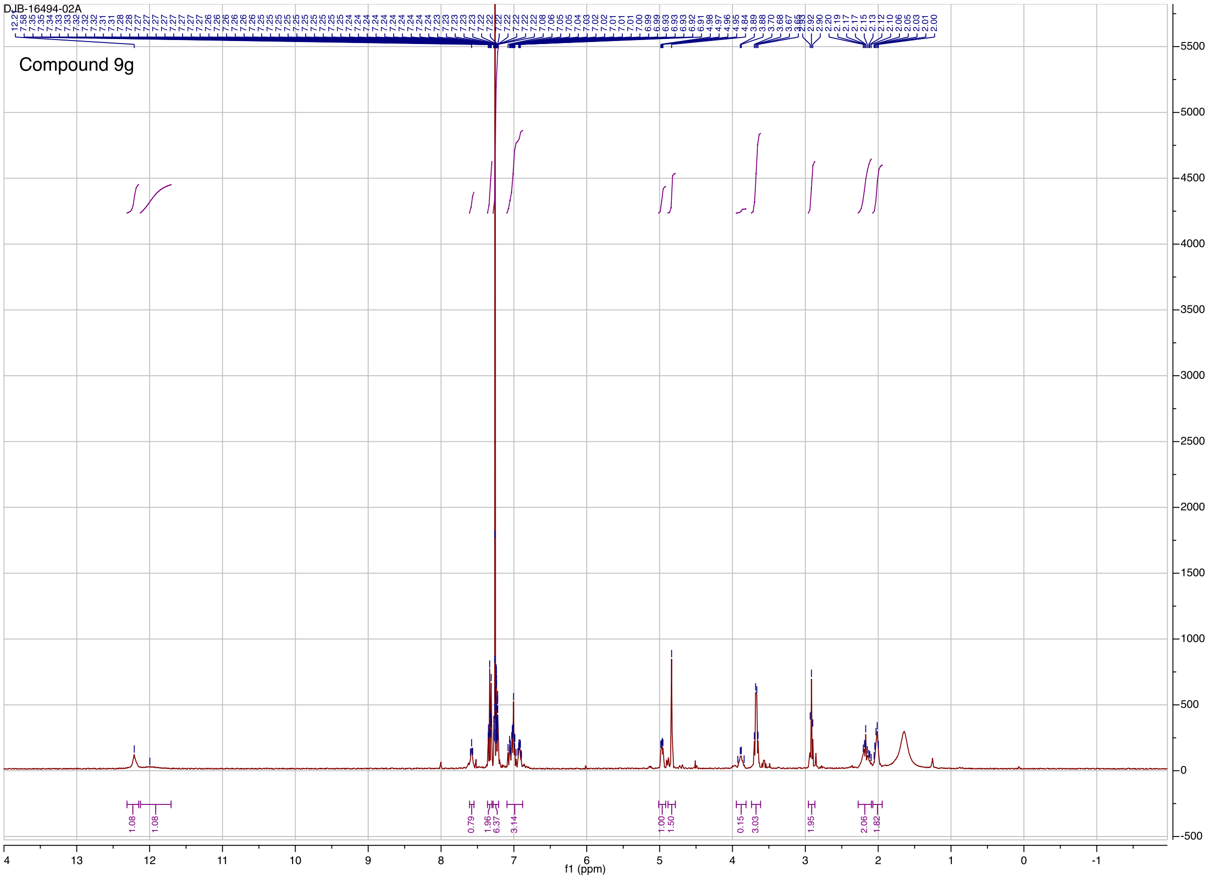
DJB-16494-02A
Compound 9g
f1 (ppm)

DJB-16494-02B

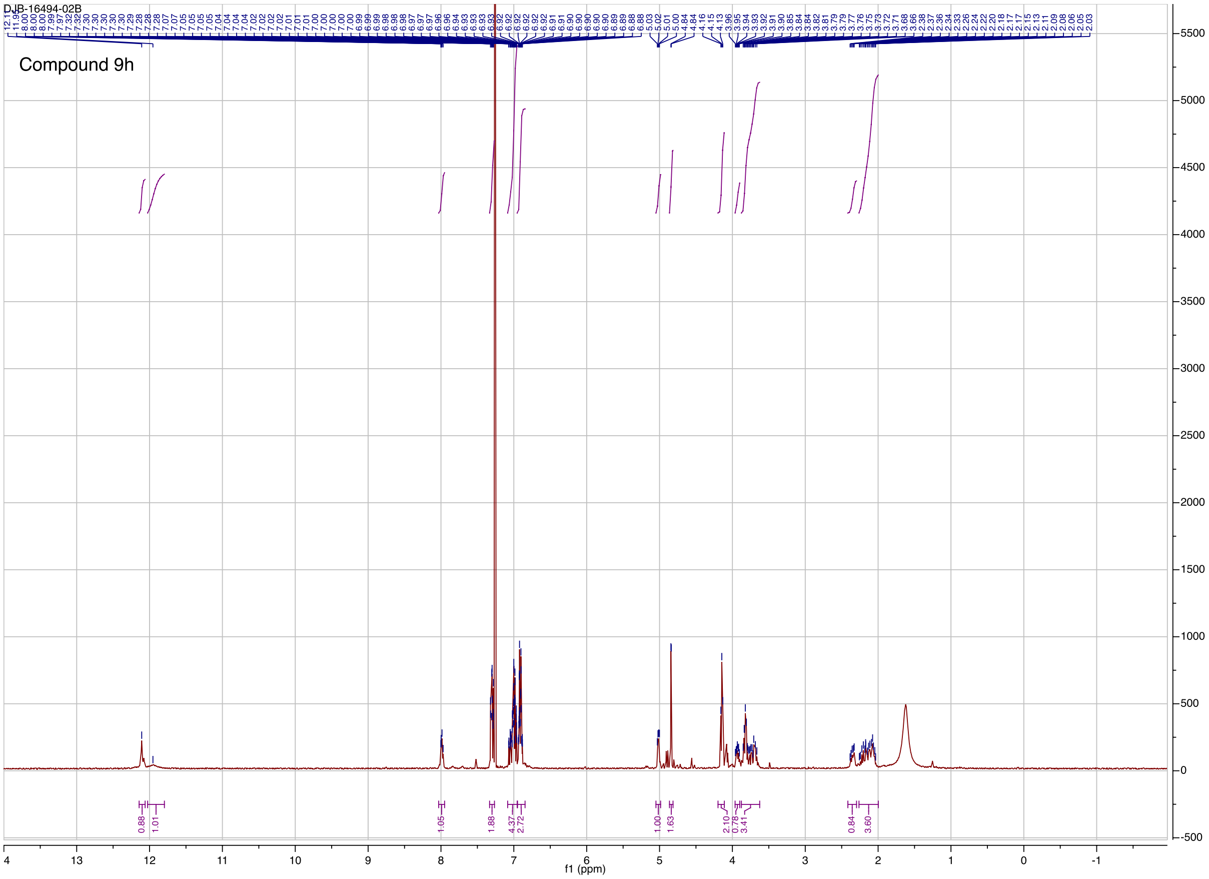

DJB-16494-23A

Compound 9i

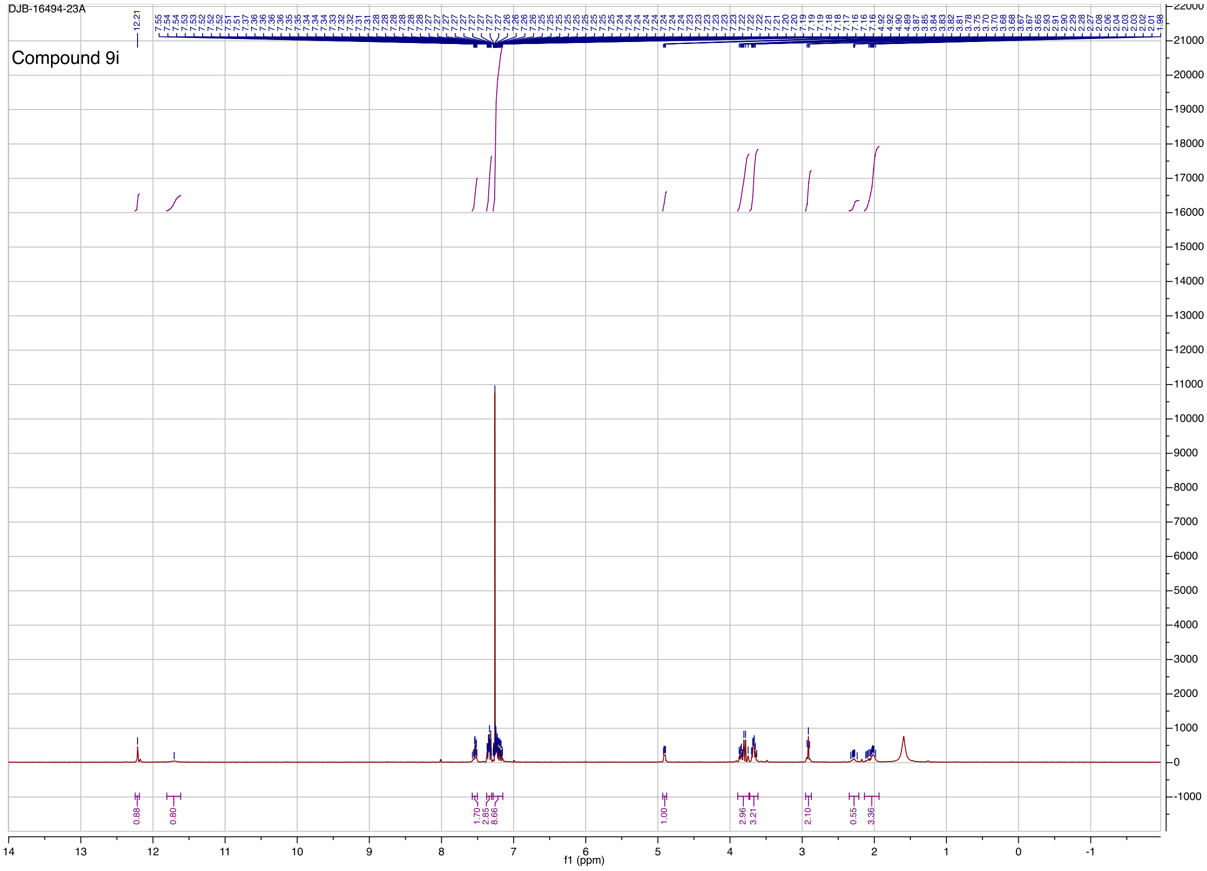

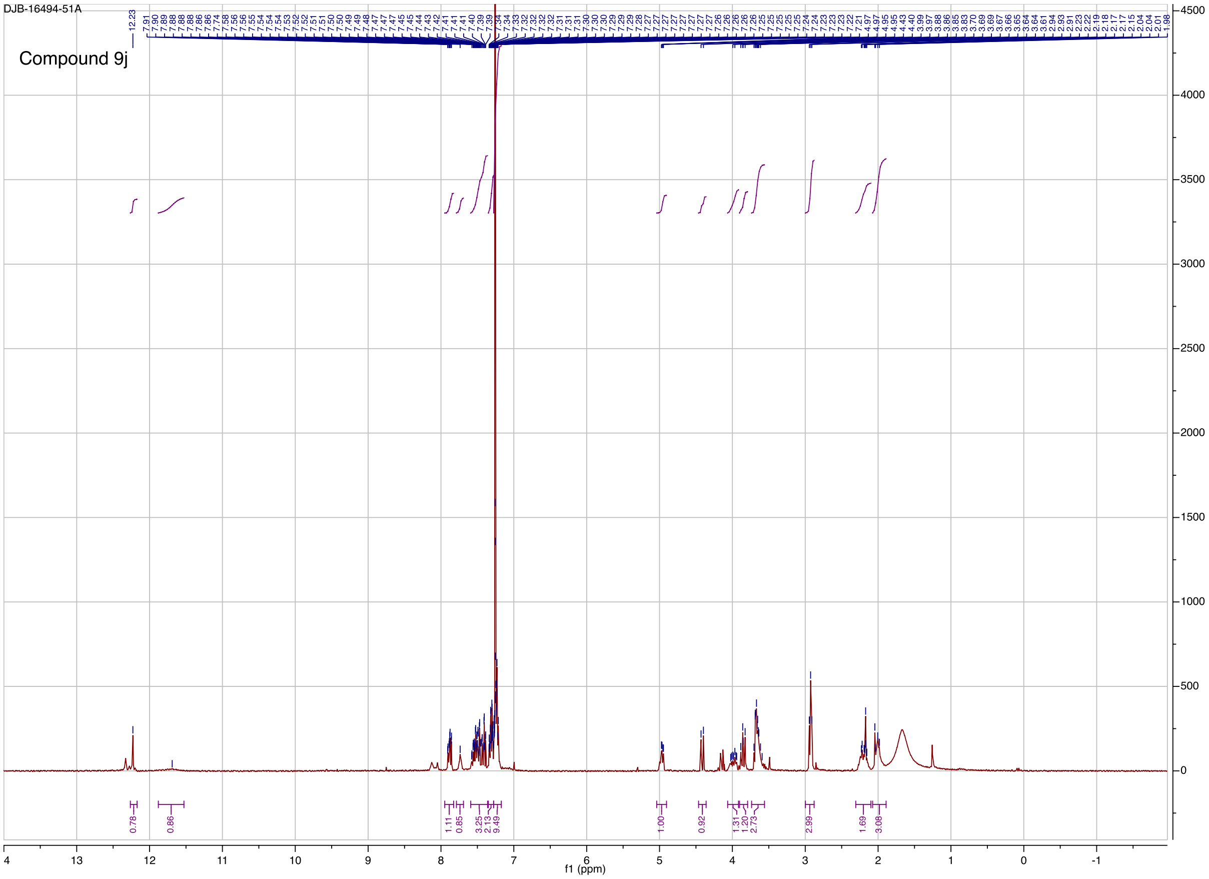
DJB-16494-51A
Compound 9j
f1 (ppm)
0.78
0.86
1.11
0.85
3.25
2.13
9.49
1.00
0.92
1.31
1.20
2.73
2.99
1.69
3.08

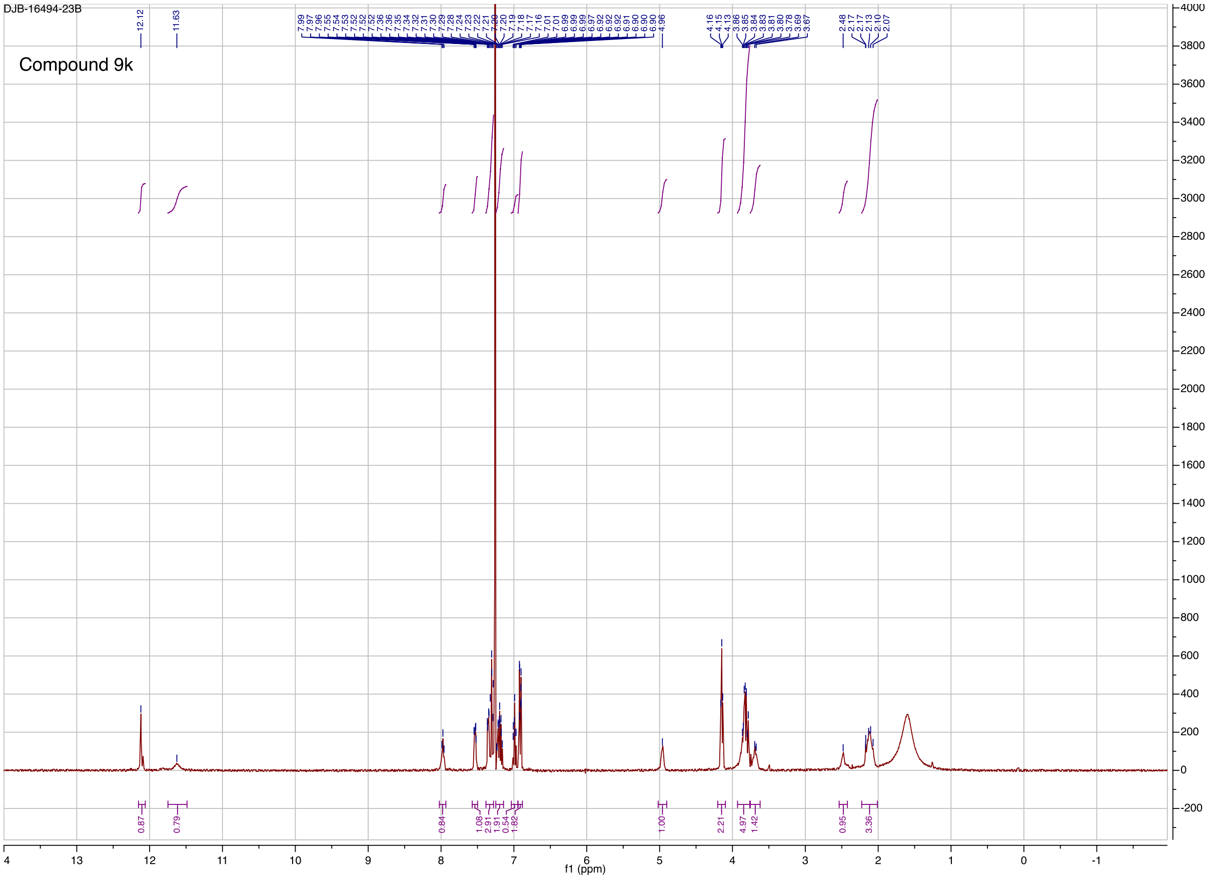

DJB-16494-23B
Compound 9k
f1 (ppm)

DJB-16494-37A

Compound 9l

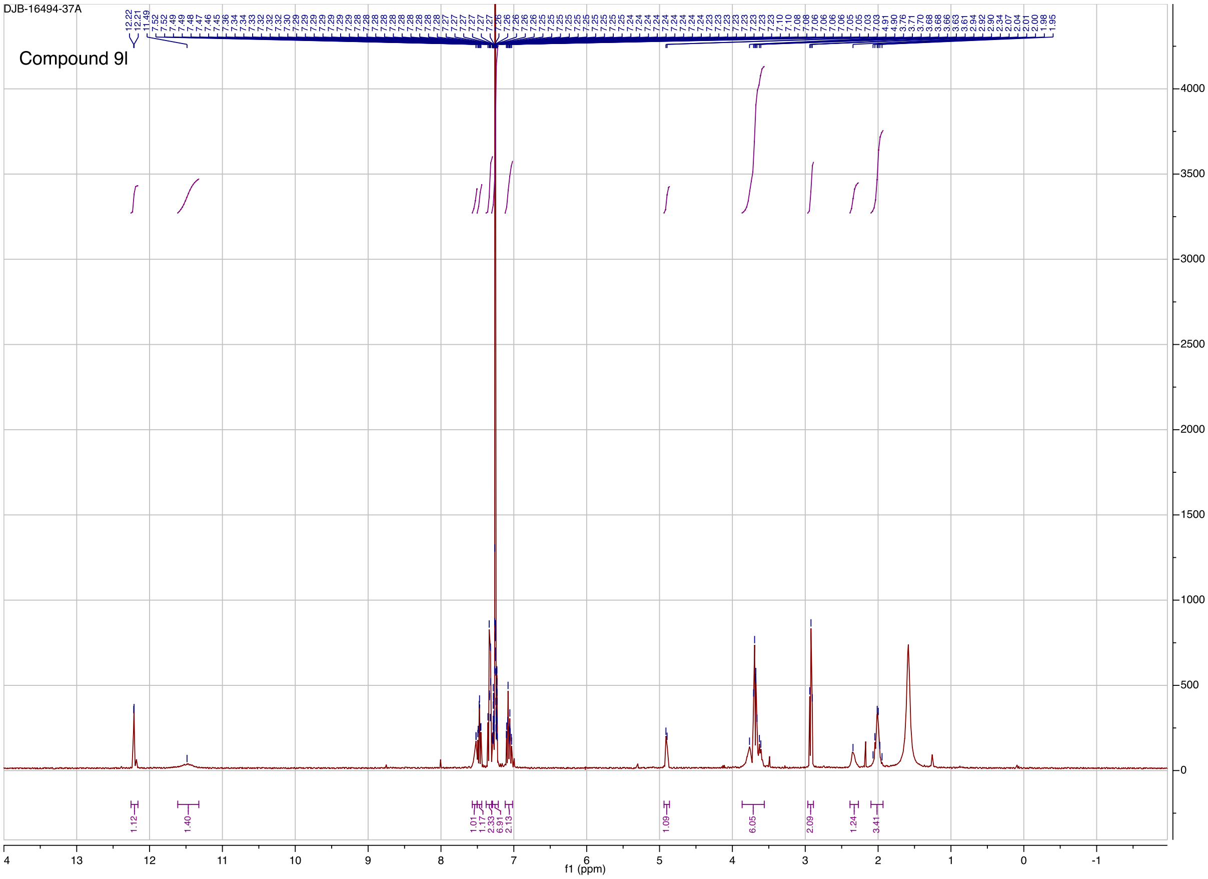

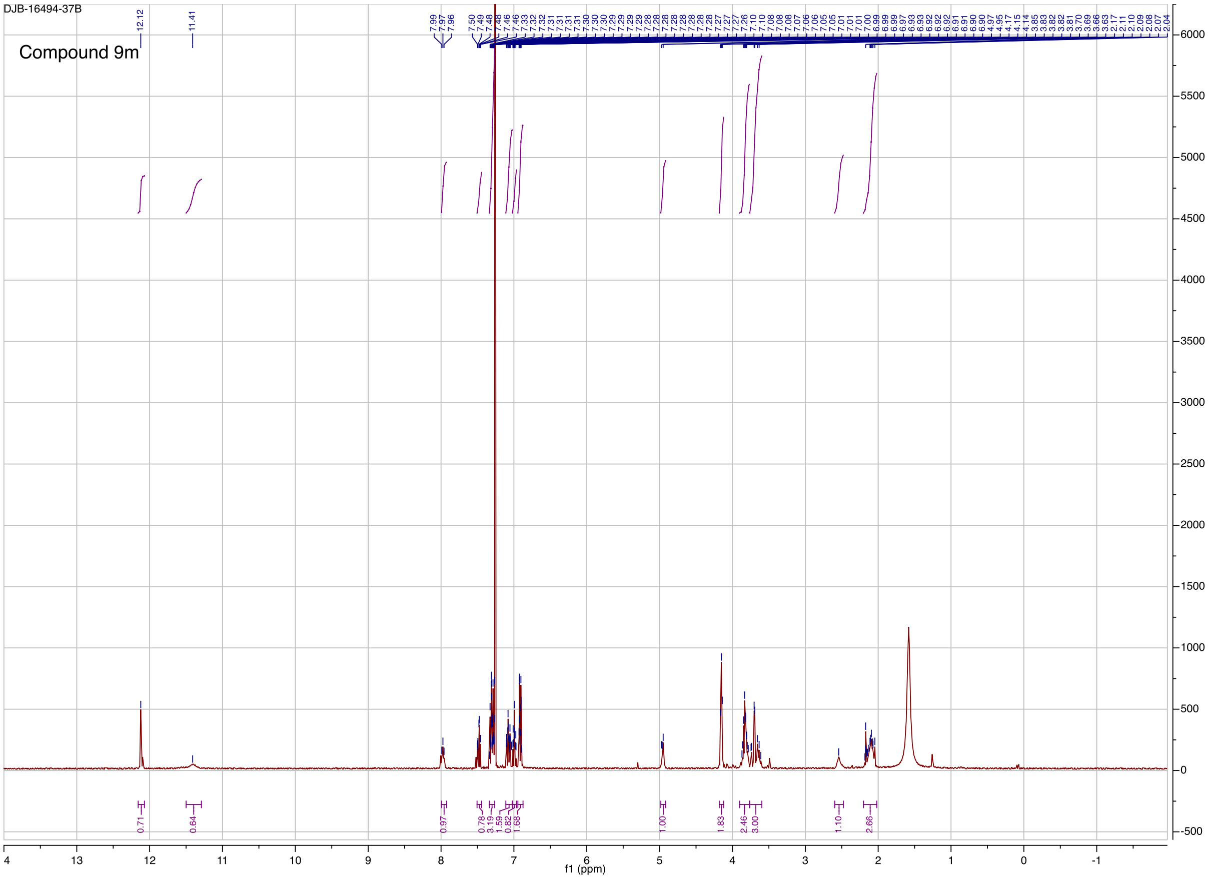

DJB-16494-37B
Compound 9m
f1 (ppm)

DJB-16453-33B

Compound 10a

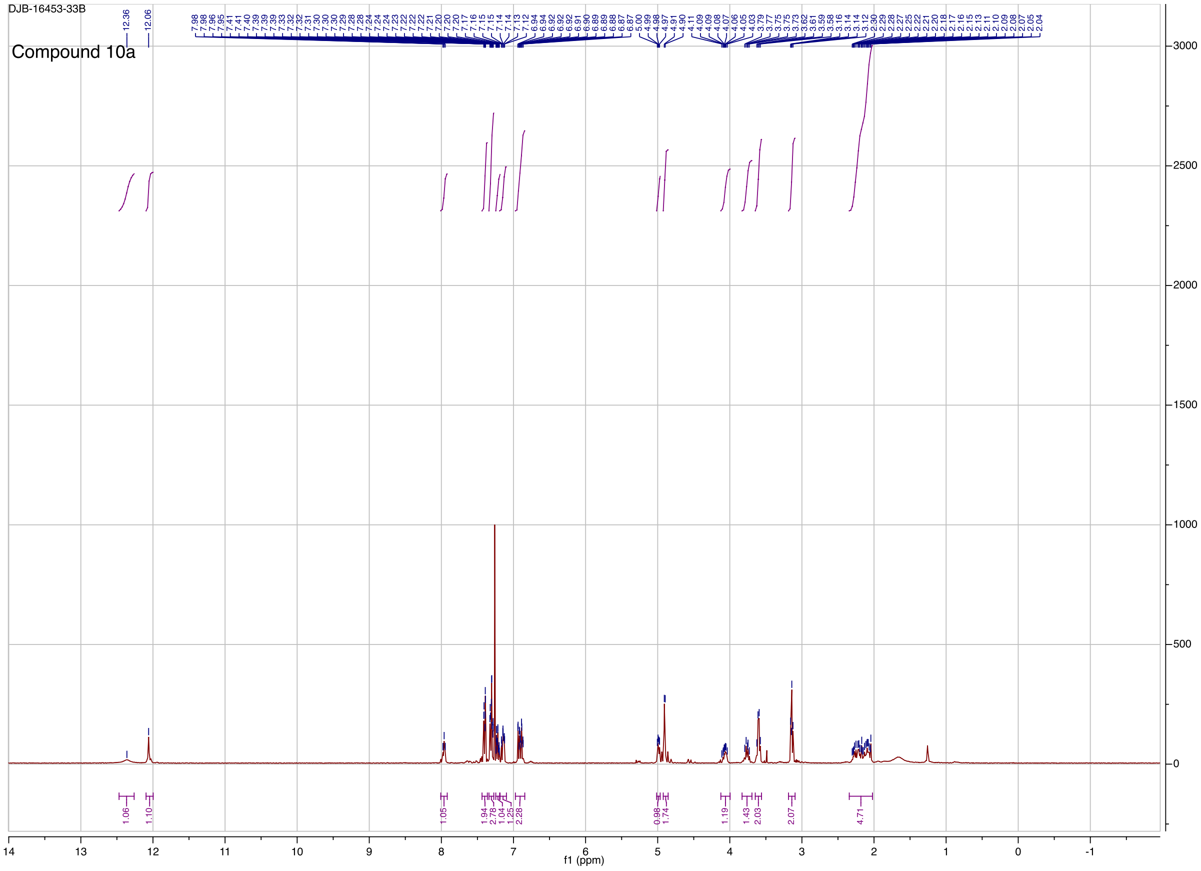

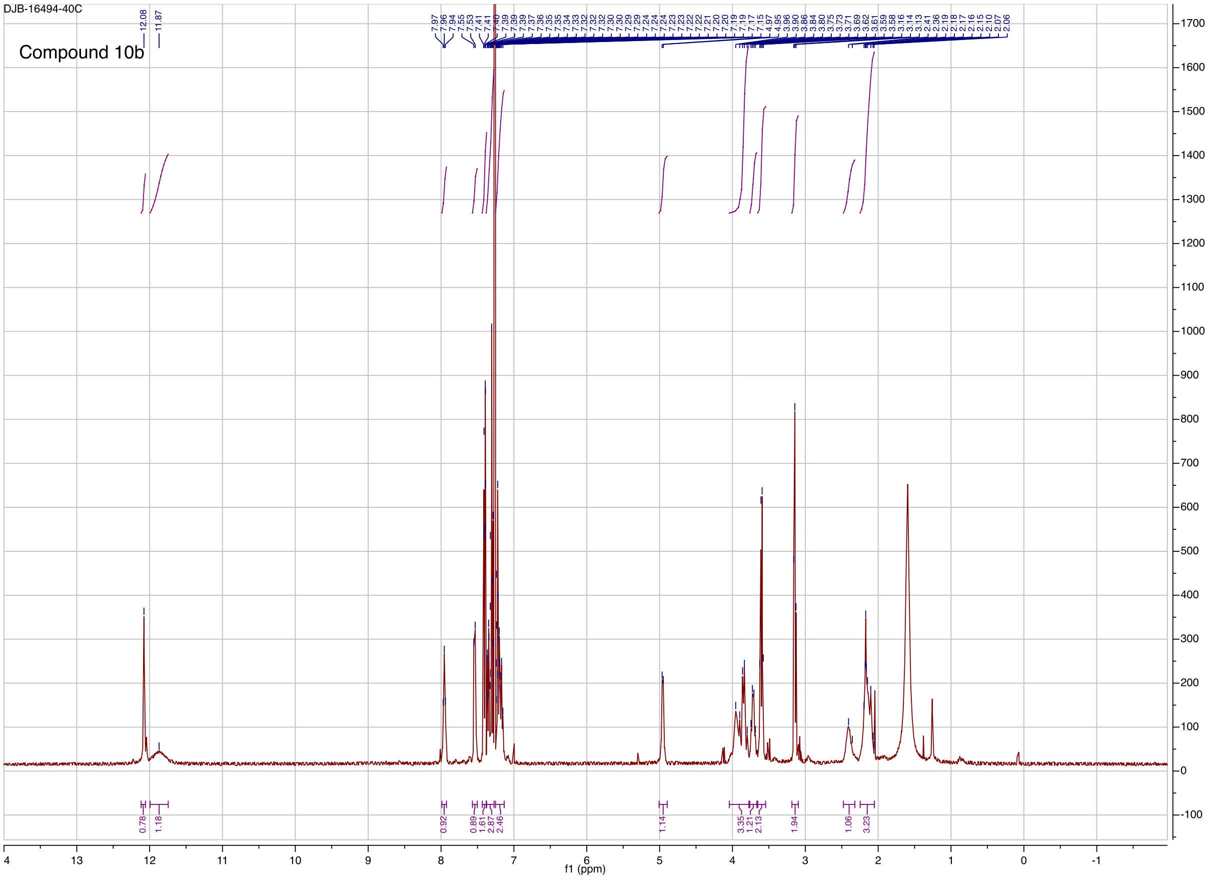
DJB-16494-40C
Compound 10b
f1 (ppm)

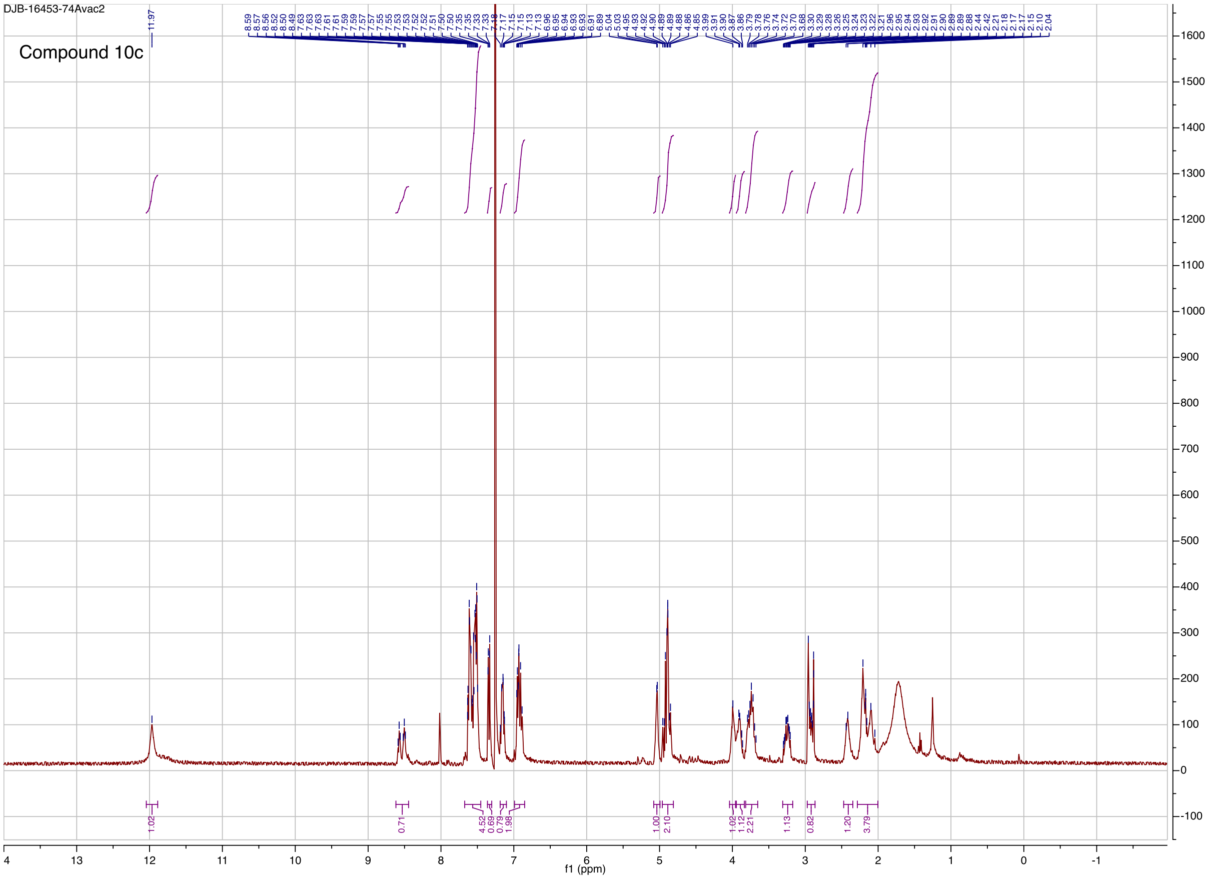
DJB-16453-74Avac2
Compound 10c
f1 (ppm)

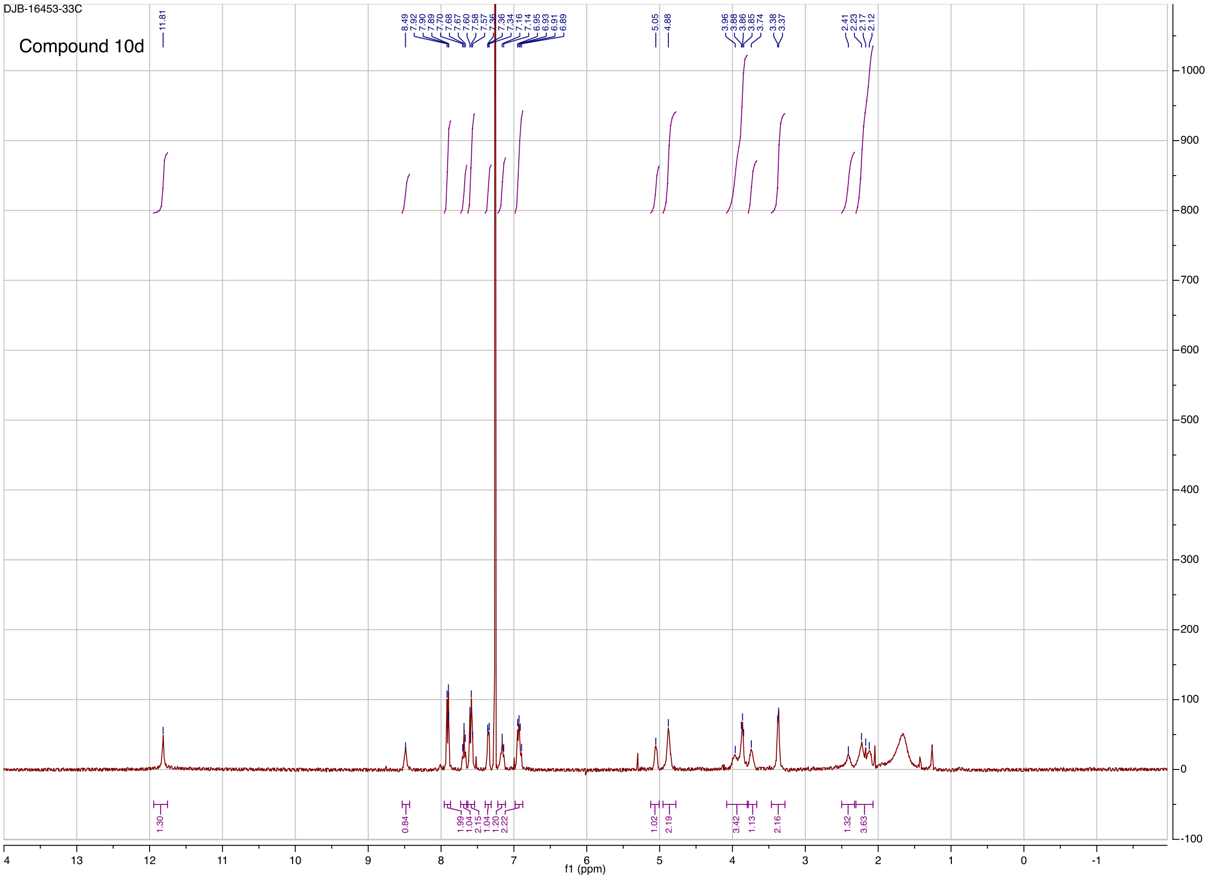
DJB-16453-33C
Compound 10d
f1 (ppm)

DJB-16494-23C

Compound 10e

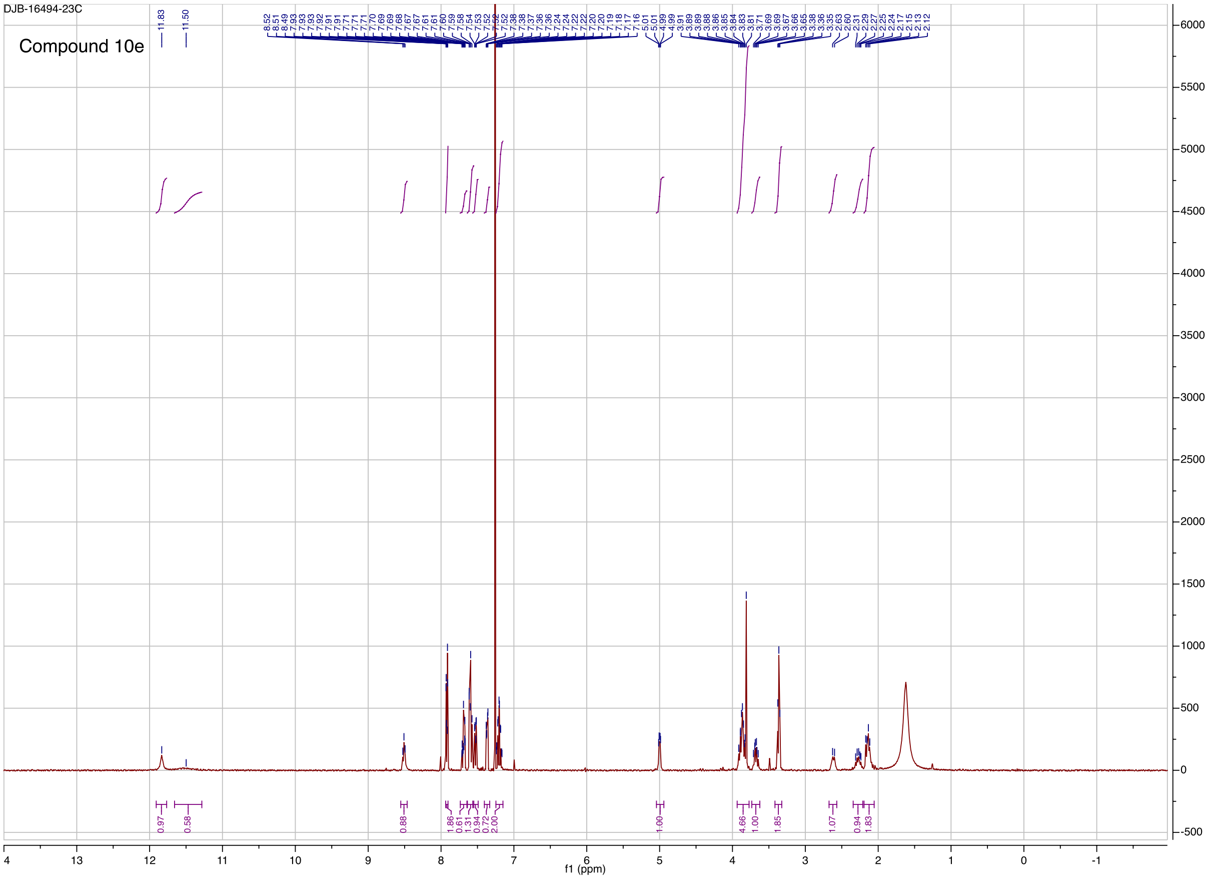

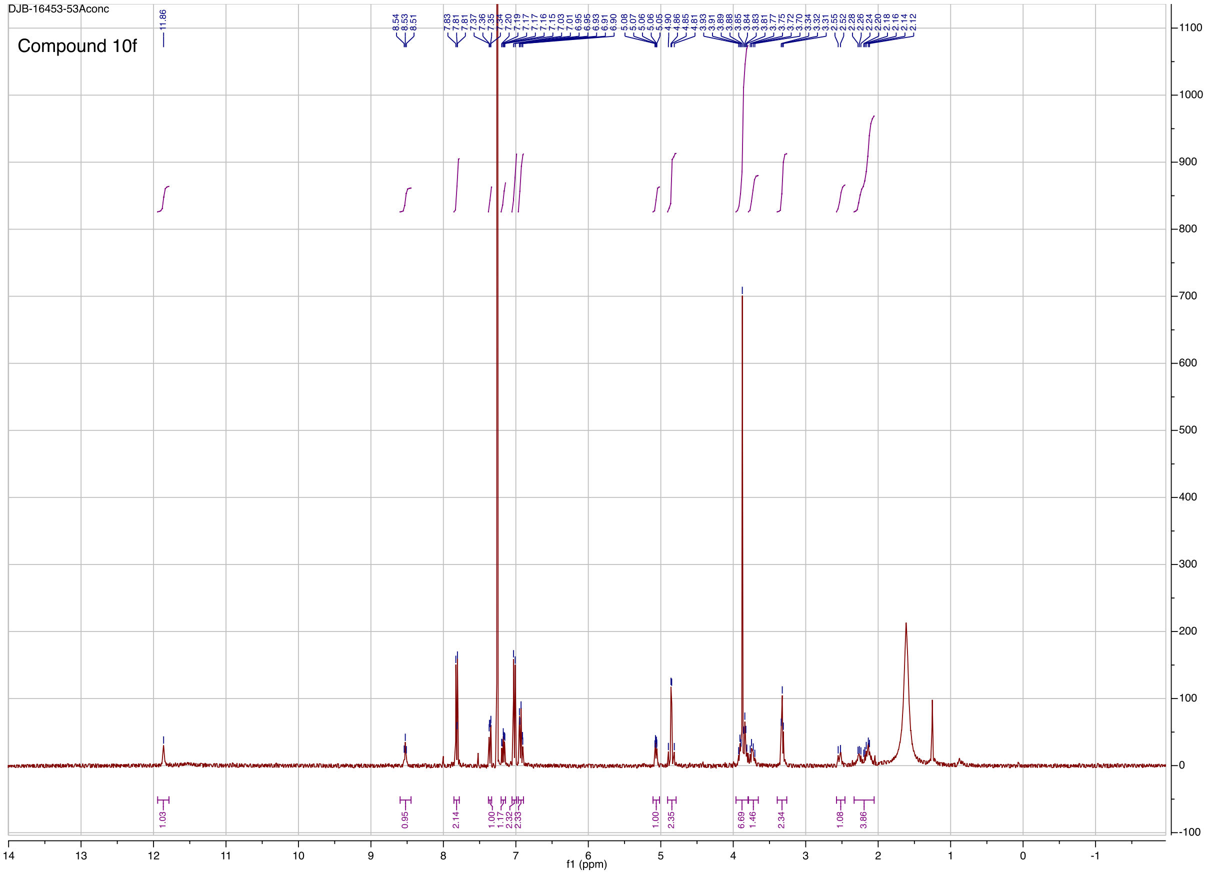
DJB-16453-53Aconc
Compound 10f
f1 (ppm)

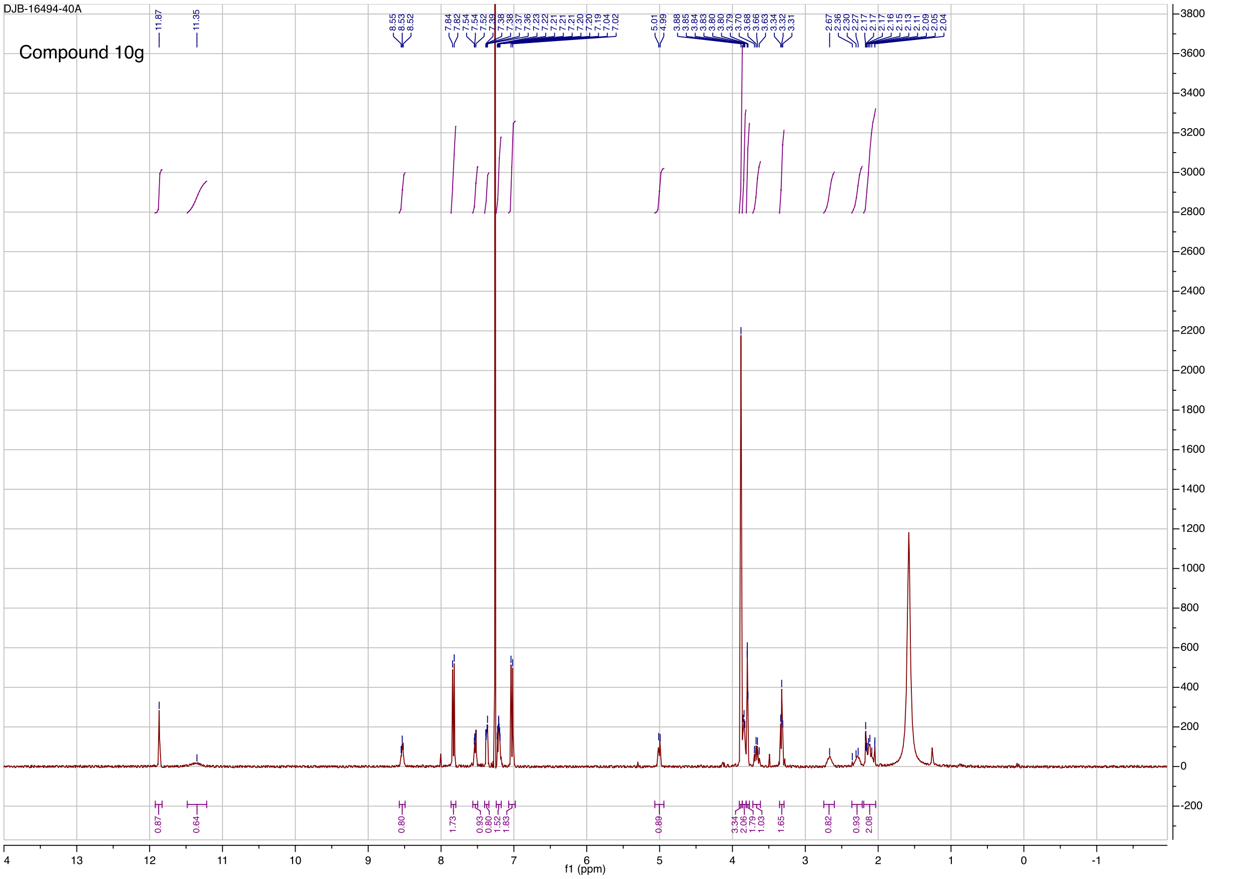
DJB-16494-40A
Compound 10g
f1 (ppm)

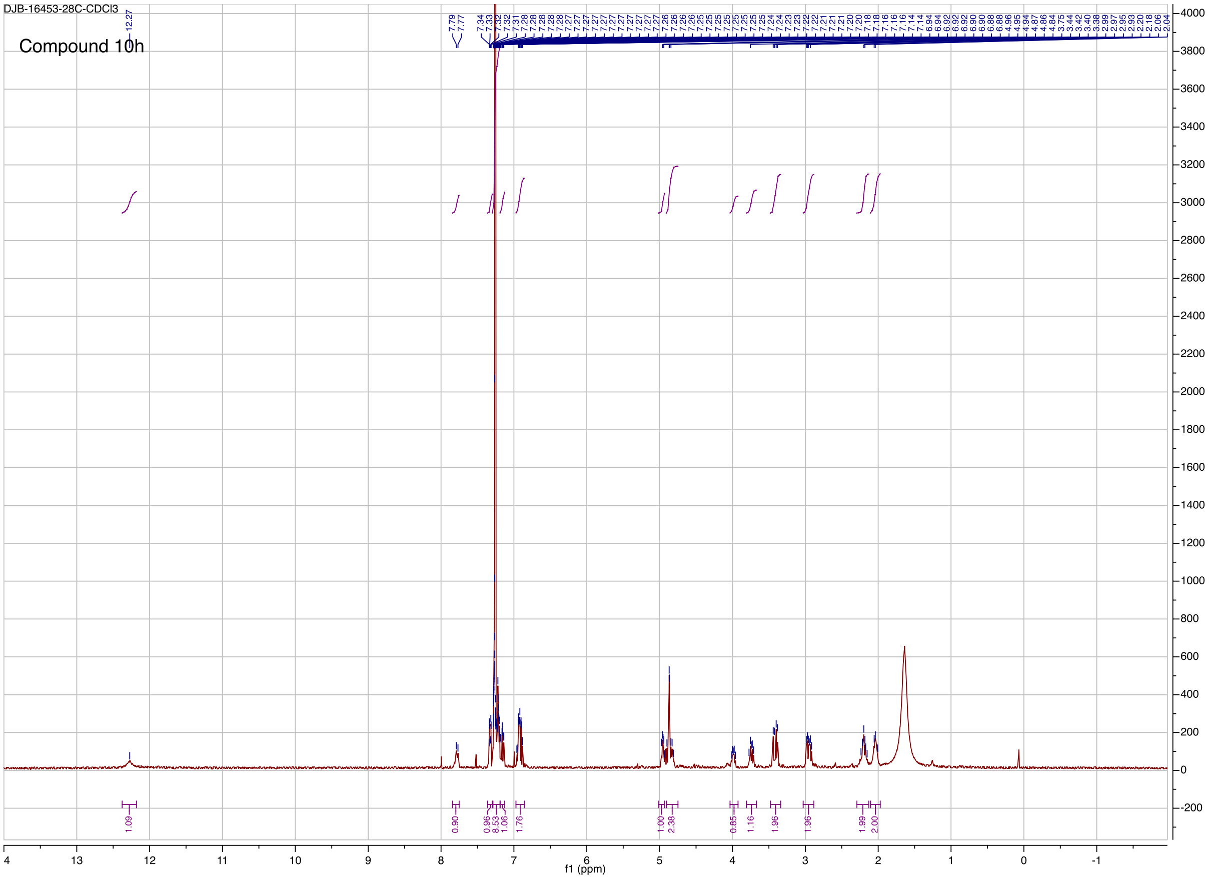

DJB-16453-28C-CDCl3
Compound 10h
f1 (ppm)

DJB-16494-29A

Compound 10i

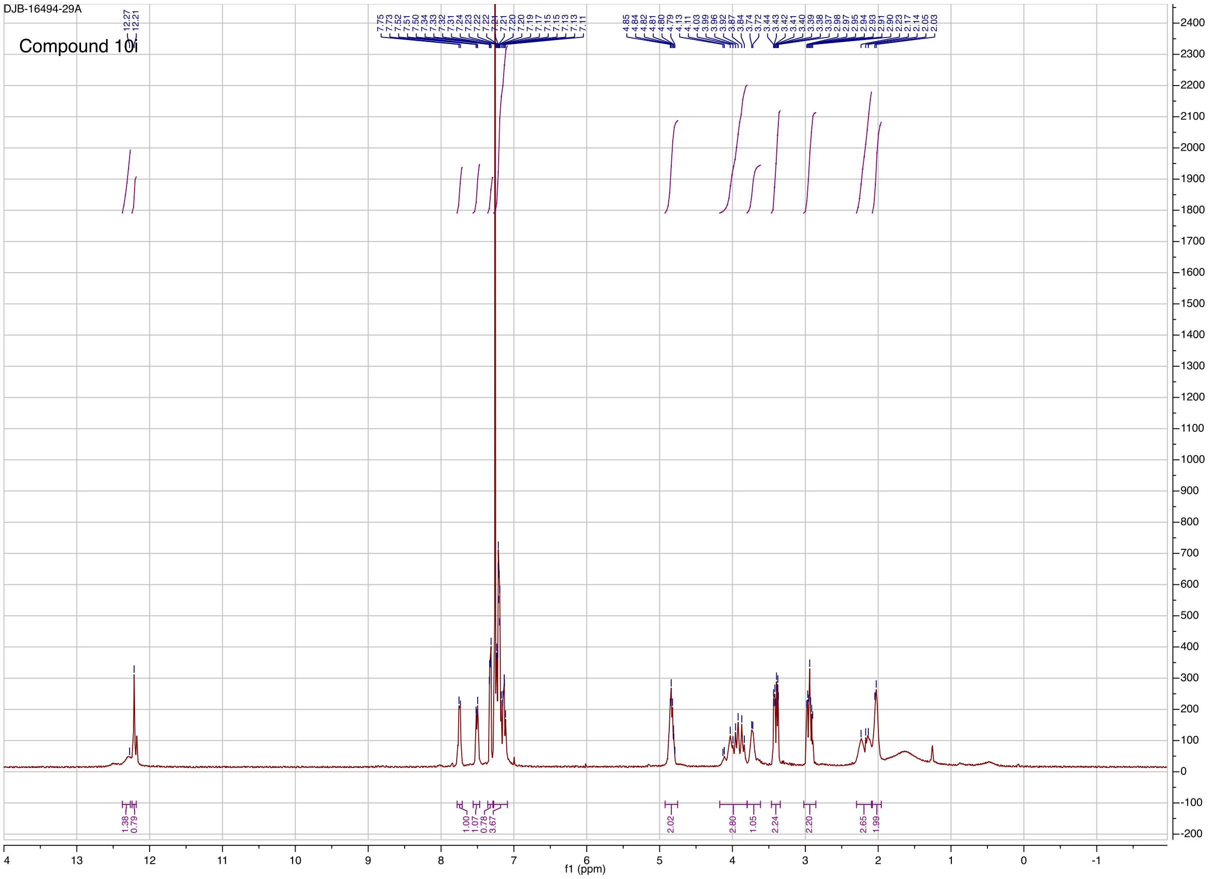

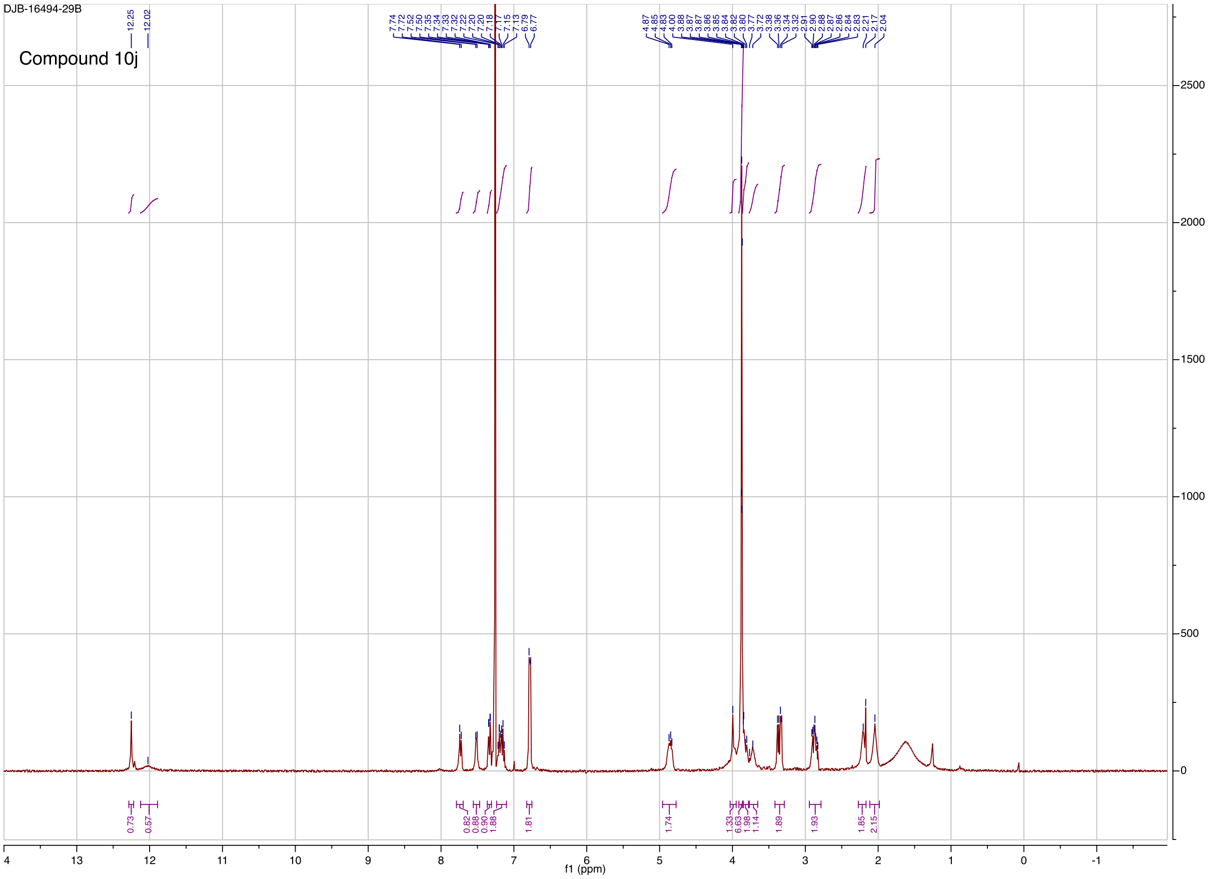

DJB-16494-29B
Compound 10j
f1 (ppm)

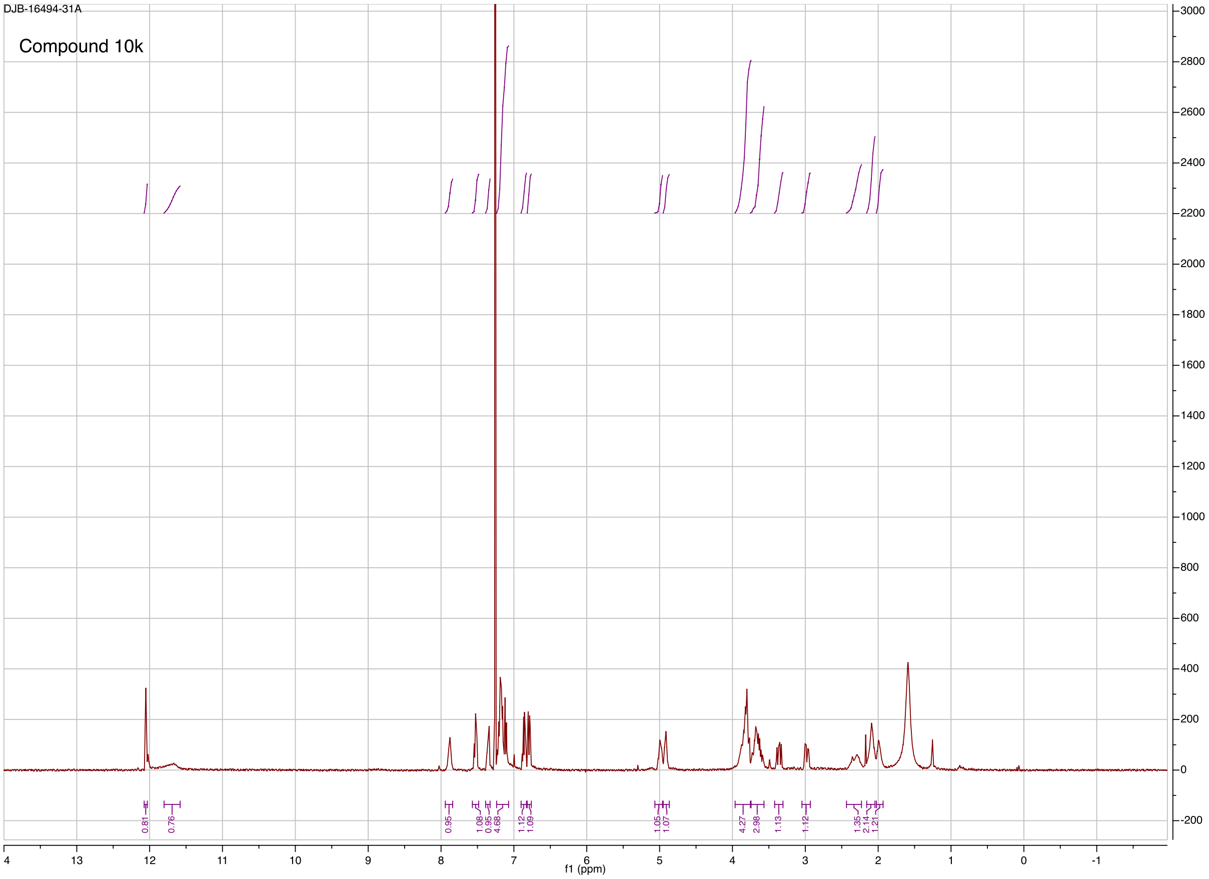
DJB-16494-31A
Compound 10k
f1 (ppm)

DJB-16494-31C

Compound 10l

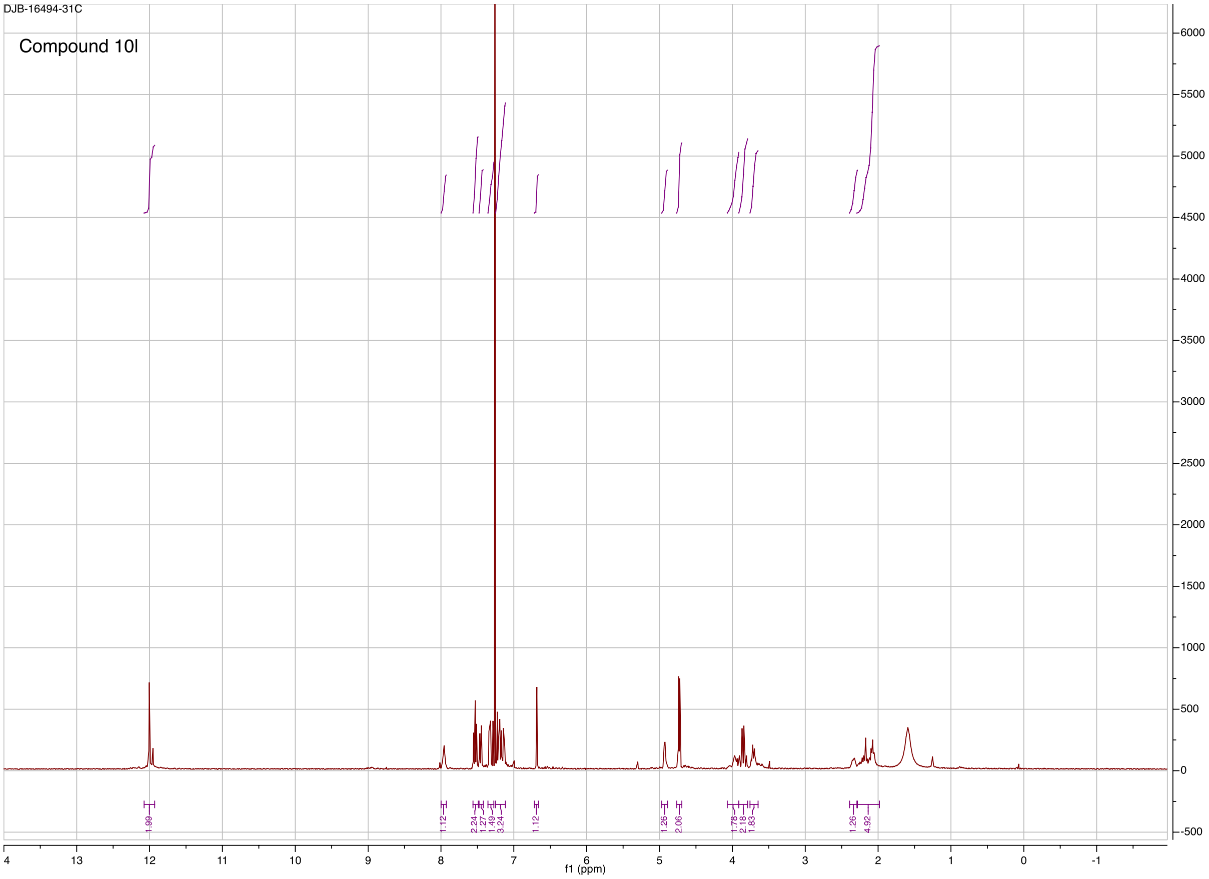

DJB-16494-18A-1

Compound 10m

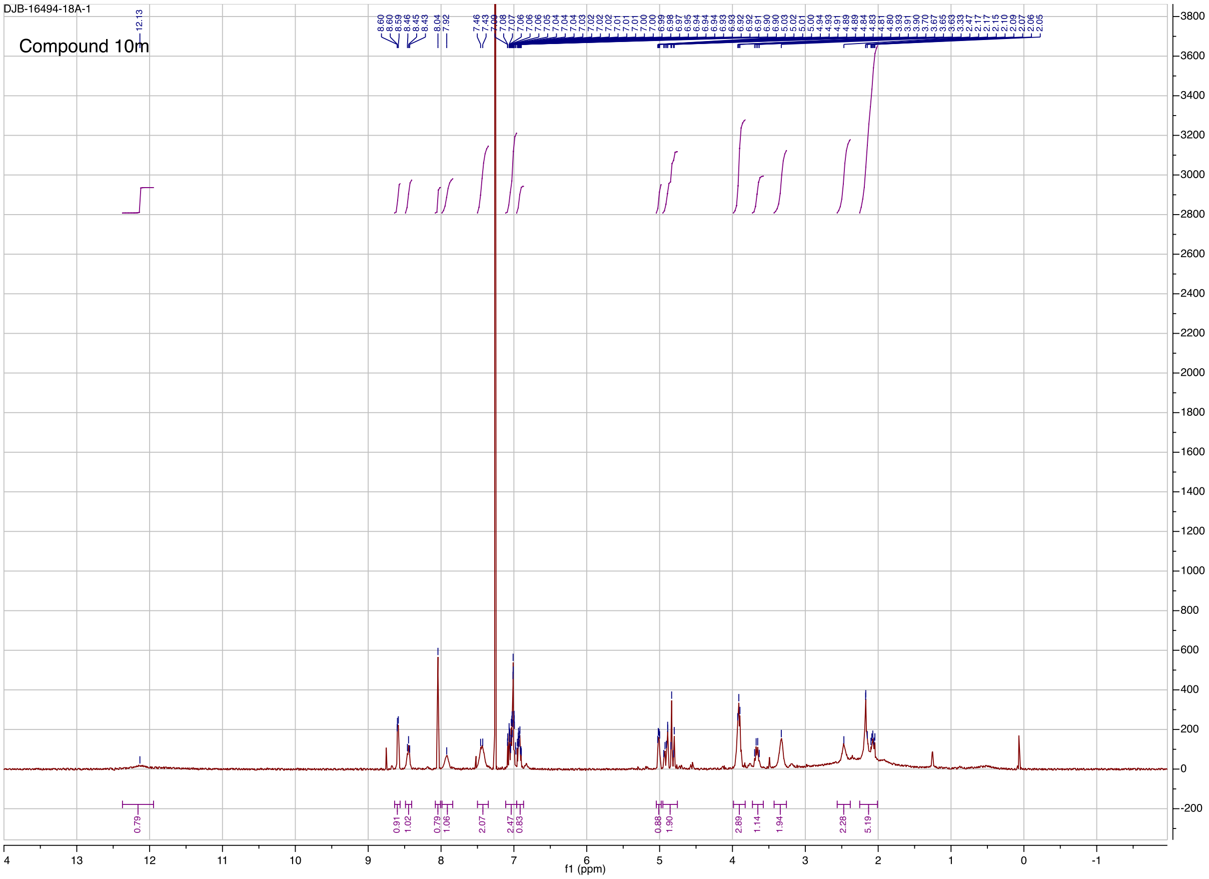

DJB-16494-18B-1

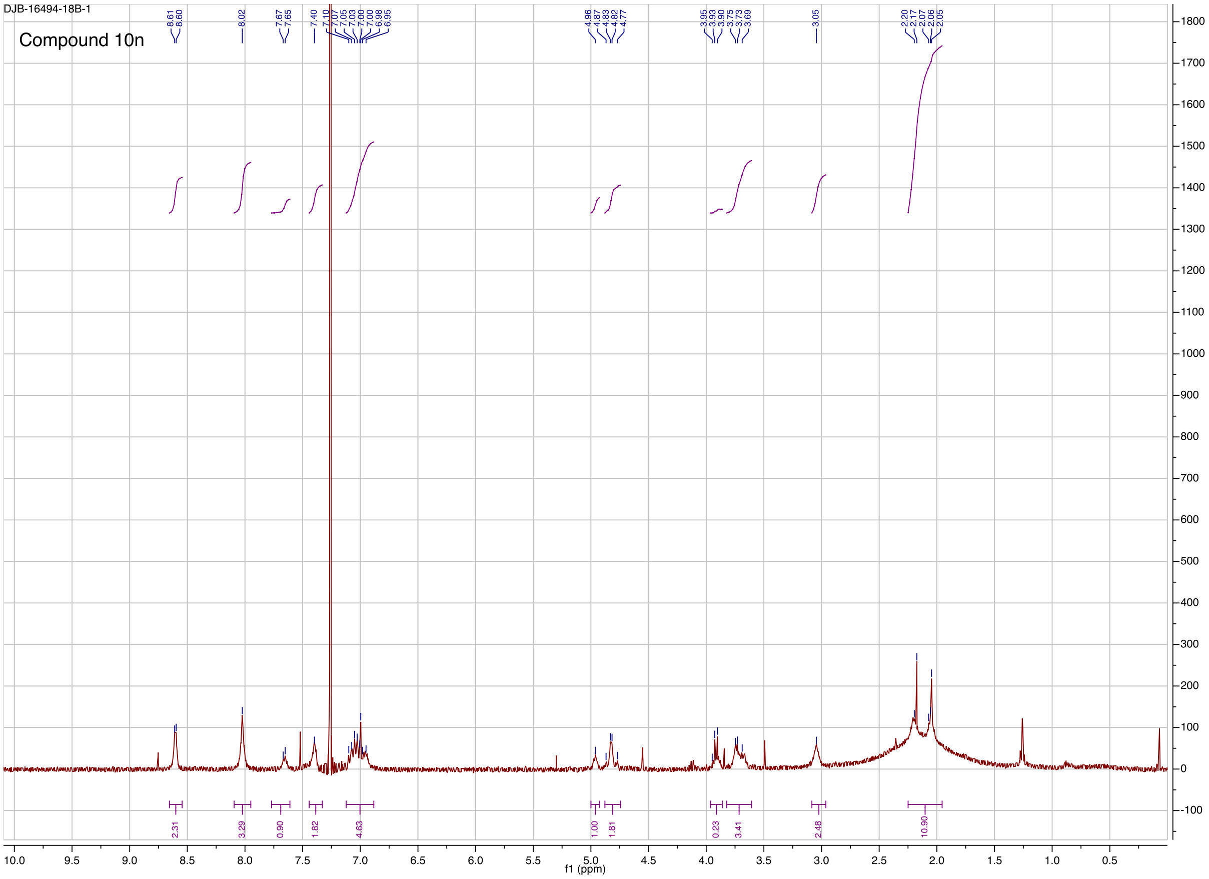

Supplement: Supplementary file 1 — Supplementary Information [file 41598_2017_17419_MOESM1_ESM.pdf]
